# Targeting a tolerogenic HLA-G genotype to tackle immune evasion and adaptive resistance in HBV-driven HCC

**DOI:** 10.1016/j.jhepr.2026.101891

**Published:** 2026-05-08

**Authors:** Janine Kah, Lisa Staffeldt, Svenja Stefanski, Natalie Herzog, Gregor Mattert, Tassilo Volz, Maximilian Voß, Kornelius Schulze, Asmus Heumann, Werner Dammermann, Sarah Kammerer, Jan-Heiner Küpper, Pablo Villavicencio-Lorini, Maura Dandri, Stefan Lüth

**Affiliations:** 1 Department of Medicine, University Medical Center Hamburg-Eppendorf, Hamburg, Germany; 2 Faculty of Health Sciences Brandenburg, Brandenburg Medical School Theodor Fontane, Brandenburg, Germany; 3 Department of Gastroenterology, Diabetology and Hepatology, University Hospital Brandenburg, Brandenburg Medical School Theodor Fontane, Brandenburg, Germany; 4 Center for Translational Medicine, Brandenburg Medical School Theodor Fontane, Brandenburg, Germany; 5 Brandenburg University of Technology Cottbus-Senftenberg, Institute of Biotechnology, Senftenberg, Germany; 6 German Center for Infection Research, Hamburg-Lübeck-Borstel Partner Site, Germany; 7 Department of General, Visceral and Thoracic Surgery, University Medical Center Hamburg-Eppendorf, Hamburg, Germany; 8 Institute of Clinical Genetics, Faculty of Health Sciences Brandenburg, Brandenburg Medical School Theodor Fontane, Brandenburg, Germany

**Keywords:** Viral hepatocarcinogenesis, Tumor–immune interaction, Immune tolerance, Patient-derived cell line, Orthotopic xenograft, Translational cancer model

## Abstract

**Background & Aims:**

HBV-associated hepatocellular carcinoma (HCC) is characterized by immune evasion and heterogeneous responses to immunotherapy. However, the mechanisms driving tumor–immune tolerance and their impact on checkpoint inhibitor-based therapies remain poorly understood. To address this gap, we generated a patient-derived HBV-induced HCC cell line that preserves a clinically relevant immune evasion mechanism mediated by human leukocyte antigen G (HLA-G) expression.

**Methods:**

We performed whole-exome sequencing on two spatially separated tumor regions to define genomic signatures with emphasis on the HLA-G locus. Patient-derived tumor cells were modified using upcyte® technology, generating the up-LC14A1 cell line. Molecular stratification included an in-house HCC cohort (n = 13), healthy donors (n = 4), serum samples from early-stage liver disease (n = 4), diagnosed HCC (n = 10), and global datasets (TCGA biopsies, n = 24; HCC, n = 120). Functional characterization included NK92 co-culture assays (1:1, 1:5, 1:10) and orthotopic transplantation in mice (HUH7, n = 3; Hep3B, n = 4; HepG2H1.3, n = 4; up-LC14A1, n = 14).

**Results:**

Whole-exome sequencing revealed a clonally coherent HCC carrying an HLA-G 3′UTR haplotype associated with immune tolerance. Cohort stratification confirmed elevated soluble HLA-G in patient #14. The derived up-LC14A1 line retained hepatobiliary and viral features and showed stable proliferation with low-level HBV DNA production. Transcriptomic profiling positioned up-LC14A1 within the HCC landscape. *In vivo*, orthotopic transplantation generated structured liver tumors and prolonged survival compared with immortalized HCC lines. The up-LC14A1 cell line resisted NK92 cytotoxicity through dynamic *HLA-G* induction, while *HLA-G* silencing restored immune killing and induced compensatory PD-L1 expression.

**Conclusions:**

The up-LC14A1 model represents a patient-derived HBV-HCC system capturing a clonally stable but dynamically regulated HLA-G-mediated immune-tolerant state. This platform enables mechanistic investigation of immune escape and provides a translational model for testing targeted immunotherapies in HBV-associated HCC.

**Impact and implications:**

HBV-associated HCC exhibits a profound immune evasion capacity and an insufficient response to immunotherapy. Nevertheless, preclinical models that mimic viral persistence and tumor resistance are underrepresented. The immune modulatory molecule HLA-G has emerged as a potent target for immunotherapy in HCCs owing to its direct suppression of T and NK cells. Here, we established a novel patient-derived HBV-HCC cell line (up-LC14A1) that mediates immune evasion via HLA-G upregulation.

## Highlights

• Patient-derived HCC represents a clinically and molecularly typical HBV-associated HCC.
• WES identifies an HLA-G-mediated immune-tolerogenic profile.
• Patient-derived up-LC14A1 cell line retains parental features.
• Immune pressure induces HLA-G expression, adaptive immune tolerance, and checkpoint switching.
• Orthotopic transplantation induces gradual *in vivo* tumor expansion, enabling evaluation of therapeutic strategies.

## Graphical abstract

**Figure:**
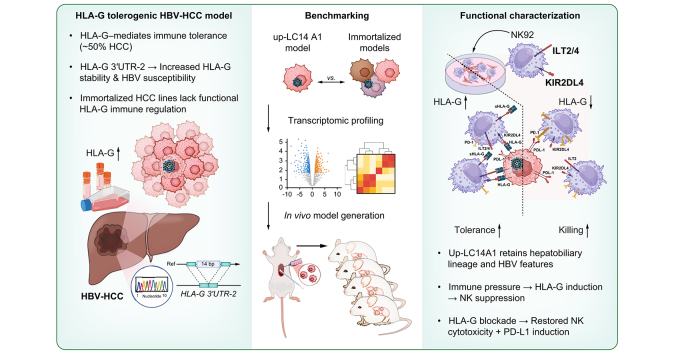

## Introduction

Hepatocellular carcinoma (HCC) accounts for up to 90% of the primary liver cancers and remains a global health burden, with chronic HBV infection being a predominant etiological factor.^1^ Despite progress in the molecular and immunological understanding of HCC, complete response rates to therapy are limited because of late-stage diagnosis, increased relapse rates, and resistance development after treatment.^2^ The difficulty lies in the heterogeneous immunosuppressive nature of HCCs, shaped by viral factors, chronic inflammation, and fibrosis, which complicates the identification of broadly effective therapeutic strategies.^3^ Remarkably, progress has been made in the treatment of HCC after the advent of sorafenib^4^ and the approval of several additional treatment regimens,^5^ including tyrosine kinase inhibitors (TKIs) and immune checkpoint inhibitors (ICIs). Nevertheless, the objective response rate following combined TKI-ICI or ICI–ICI therapy remains unsatisfactory.^3^ Moreover, patients who develop resistance may exhibit hyperprogressive relapses leading to a worse clinical setting. To improve therapy outcomes for these refractory patients, evaluating novel targets is necessary to manage resistance and prevent its development during therapy.

Among the pleotropic immune evasion mechanisms orchestrated in HCCs, the non-classical major histocompatibility complex (MHC) class I molecule human leukocyte antigen G (HLA-G), found in 50% of primary HCCs,^6^ has emerged as a potent immune checkpoint.^7^ Unlike programmed death-ligand 1 (PD-L1), HLA-G mediates immune suppression via distinct pathways, inhibiting cytotoxic T cells and NK cells, promoting tolerogenic dendritic cells, and facilitating the expansion of regulatory T cells.^7^ Elevated expression of membrane-bound (mHLA-G) and soluble HLA-G (sHLA-G) has been correlated with tumor progression, high recurrence rates, and resistance to ICI therapy in HCCs.^6^^,^^8^^,^^9^ In the setting of HBV infection, miR-152, a functional suppressor of HLA-G stability, is reduced, leading to an overexpression of the immune tolerance molecule during active infection.^10^ Moreover, genetic polymorphisms in the *HLA-G* gene have reportedly correlate with increased susceptibility to HBV infection.^11^ Its overall immunomodulatory properties make HLA-G an interesting target after immunotherapies, especially in virus-induced HCCs, where it can induce resistance mechanisms.

Commonly used immortalized HCC cell lines fail to process the functional immunomodulatory protein,^12^ even when they synthesize *HLA-G* mRNA;^6^ therefore, genetically modified cell lines remain the gold standard for elucidating HLA-G-mediated immune tolerance. However, suitable models remain lacking for robust investigation of the dynamic immune-tolerogenic pathways and the resulting phenotype adaptations.

Here, we address this unmet need and establish an HBV-HCC patient-derived model that displays the dynamics of HLA-G-mediated immune tolerance. The advantage of this model lies in its low *in vivo* progression rate, which enables orthotopic transplantation, and its retention of original tumor features. This model was generated from an HBV-positive individual exhibiting mixed hepatocellular (70%) and cholangiocellular (30%) carcinoma, carrying HLA-A∗03:01P and ∗30:01P alleles. The patient was initially treated with a nucleotide analog (tenofovir) and subsequently with atezolizumab plus bevacizumab upon relapse; however, the patient experienced rapid disease progression and succumbed 18 months after resection. From the tumor center material, we extracted proliferative competent primary liver cancer cells (LC14) and subsequently modified them genetically using upcyte® technology to enable long-term culture and ongoing proliferation (designated up-LC14A1). The predictive transcriptomic classification clustered the individual cell line up-LC14A1 with HBV-replicating HepG2H1.3 cells, indicating the preservation of virus-associated molecular signatures. Although pathway enrichment and disease association analyses confirmed a strong alignment with liver cancer profiles, we demonstrate that up-LC14A1 cells maintain patient characteristics and dynamic HLA-G expression, both *in vitro* and *in vivo*.

## Material and methods

### Study approval, human material, and animal experiments

Human tissue and blood were collected, processed, preserved, and ethically approved as described previously^13^^,^^14^ and listed in Table S1. HLA-A typing of patient- and healthy donor-derived tissue and blood cells was performed as described previously.^13^ The study was approved by the Ethical Review Committee of the Ärztekammer Hamburg (PV-3578) and conducted in accordance with national guidelines and the 1975 Declaration of Helsinki. Animal experiments were performed in accordance with the European Communities Council Directive (86/EEC) and approved by the City of Hamburg, Germany (N056/2020). Mouse generation, breeding, housing, and surgical procedures were conducted as described previously.^14^

### Generation of up-LC14 cells

Patient-derived HCC cells were isolated as described previously^14^ and genetically modified to generate proliferation-competent cultures following established protocols for primary hepatocytes.^15^^,^^16^ Cells were expanded under standard HCC culture conditions and cryopreserved for long-term storage. Detailed transduction, culture, and freezing procedures are provided in the Supplementary Methods.

### Cell culture, genetic labeling, and spheroid formation

After thawing, up-LC14 cells were maintained in advanced DMEM/F-12-based culture conditions. Stable integration of the LeGO-iG2-Puro+-Luc2 construct by lentiviral transduction was performed as described previously.^14^ Immortalized HCC lines (HUH-7, Hep3B, and HepG2-H1.3) were maintained as described previously.^17^^,^^18^ For two-dimensional (2D) experiments, cells were seeded in 96- or 24-well plates and treated after 24 h. For three-dimensional (3D) assays, luciferase (LUC)-transduced up-LC14 cells were cultured in ultra-low attachment conditions to monitor spheroid formation over time. Full media compositions, seeding densities, siRNA transfection description, and imaging settings are provided in the Supplementary Methods.

### NK92 co-culture experiments and real-time viability monitoring

NK92 cells were expanded in IL-2-supplemented culture conditions and applied to target cells at effector-to-target ratios of 1:1, 1:5, and 1:10. Supernatants and/or cells were collected at indicated time points for downstream analyses. Target-cell viability was monitored in real time using xCELLigence-based impedance measurements (ACEA Biosciences, 92121 San Diego, USA) . Detailed NK92 culture conditions and downstream readouts are provided in the Supplementary Methods.

### Molecular analyses

We isolated RNA and DNA from cell lines and tissue using column-based kits.^13^^,^^14^^,^^19^ Gene expression was quantified by TaqMan-based qPCR (Table S2) following cDNA synthesis. Whole-exome sequencing (WES) and RNA sequencing (RNA-seq) were performed on Illumina platforms (Illumina, San Diego, CA, USA) and analyzed using established pipelines; downstream analyses were conducted in R Studio version 4.5.3 (R Foundation for Statistical Computing, Vienna, Austria). Detailed library preparation, sequencing, and bioinformatic workflows are provided in the Supplementary Methods.

### Flow cytometry, immunofluorescence, and protein measurements

Flow cytometry was performed using antibody panels listed in Tables S3 and S4.^14^ Immunofluorescence staining of cultured cells and tissue cryosections was performed using primary antibodies listed in Table S5 and fluorescent secondary antibodies. We quantified sHLA-G (Thermo Fisher, Waltham, MA 02451, USA), human alpha-1 antitrypsin (AAT, abcam, Cambridge, MA 02139, USA), and perforin by ELISA according to manufacturers’ instructions . Full assay conditions and reagent details are provided in the Supplementary Methods.

### Viral DNA and cfDNA isolation and analysis

HBV DNA was quantified by TaqMan qPCR using cloned standards.^19^ Cell-free DNA (cfDNA) was isolated from supernatants; fragment analyses were performed by Alu-based qPCR (Table S6) and locus-specific HLA-G assay (Table S2). Full assay conditions and reagent details are provided in the Supplementary Methods.

### Statistical analysis

Graphs and statistical analyses were performed in GraphPad Prism version 10 (GraphPad Software Inc., La Jolla, CA, USA). Statistical tests and replicate numbers are detailed in the Supplementary Methods and/or figure legends. Significance thresholds were defined as ∗*p* <0.05, ∗∗*p* ≤0.01, ∗∗∗*p* ≤0.001, and ∗∗∗∗*p* ≤0.0001.

## Results

### Identification of the HLA-G genetic footprint reveals an immune-modulatory genomic profile regulated by HLA-G

**Fig. 1:**
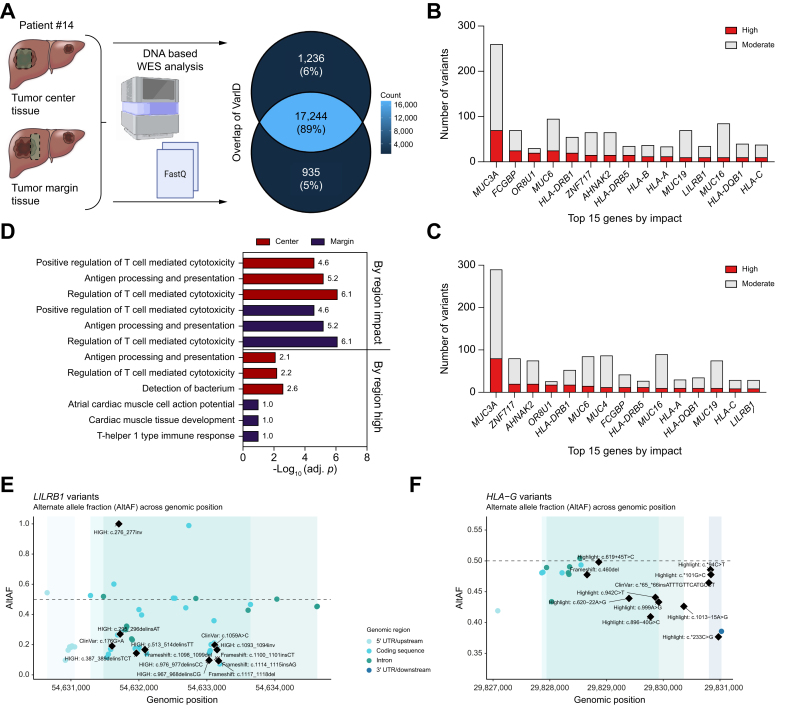
Spatial whole-exome sequencing (WES) of patient’s tumor center and margin tissue. (A) Spatial WES was performed on tumor center and margin from an HBV-associated hepatocellular carcinoma (HCC) resection (patient #14; n = 2 technical replicates/sample). Shared and region-specific variants are summarized by Venn diagram. (B) Top 15 variant-enriched genes are shown for (C) center and (D) margin, stratified by predicted impact (high, red; moderate, green). (D) Gene ontology biological process enrichment was conducted on genes harboring high/moderate variants; bars indicate -log10 adjusted *p* values. Variant positions and alternate allele fraction (AltAF) are shown for (E) *LILRB1* and (F) *HLA-G*, annotated by genomic context; dashed line denotes median AltAF.

To classify the patient material, we performed WES on two spatially separated regions of the resected tumor—the center and margin (Fig. 1A). Both regions showed highly overlapping variant profiles, indicating a shared clonal origin and enabling patient-level classification based on stable genomic features rather than spatial heterogeneity (Fig. 1A and Fig. S1). After filtering for putative functional variants (non-synonymous, high/moderate impact, variant allele frequency [VAF] ≥0.05), variant numbers, impact distributions, and VAF profiles were comparable between regions, consistent with malignant tissue of similar purity.

Genes with the highest variant burden were largely shared across regions (Fig. 1B,C). The top 15 variant-enriched genes included mucin-associated and structural genes (*e.g. MUC3A, MUC6, ZNF717,* and *AHNAK2*) and several immune-interaction genes within the HLA locus (*HLA-DQB1, HLA-DRB1/5,* and *HLA-C*) as well as the HLA-G receptor *LILRB1*. Despite minor ranking differences, the overall gene composition and the predominance of moderate-impact variants remained consistent, indicating that immune-interaction pathways represent a core genomic feature rather than a region-specific adaptation. Pathway enrichment of region-specific high-impact variants confirmed significant enrichment in antigen processing and presentation, and regulation of T-cell-mediated cytotoxicity (Fig. 1D).

We next examined variants in the *LILRB1* and *HLA-G* loci (Fig. 1E,F). The HLA-G locus contained the heterozygous 14-bp insertion (rs371194629), rs1063320 G, and rs1049033, variants associated with HBV susceptibility and HCC risk.^11^^,^^20^ Additional polymorphisms (rs1130363, rs915667, rs1707, rs1710, and rs1610696) localize to the regulatory 3′UTR and form extended haplotypic blocks that affect transcript stability, microRNA binding, and the balance between sHLA-G and mHLA-G isoforms.^21^ Together, the 14-bp insertion, rs1063320 G, and rs1610696 G alleles are most consistent with the canonical *HLA-G* 3′UTR UTR-2 haplotype—a regulatory configuration associated with immune tolerance and persistent viral infection.^22^^,^^23^ This defines a patient-specific *HLA-G* haplotype compatible with enhanced immune-tolerogenic signaling.

### Generation of a patient-derived HBV-HCC model with high HLA-G expression

**Fig. 2:**
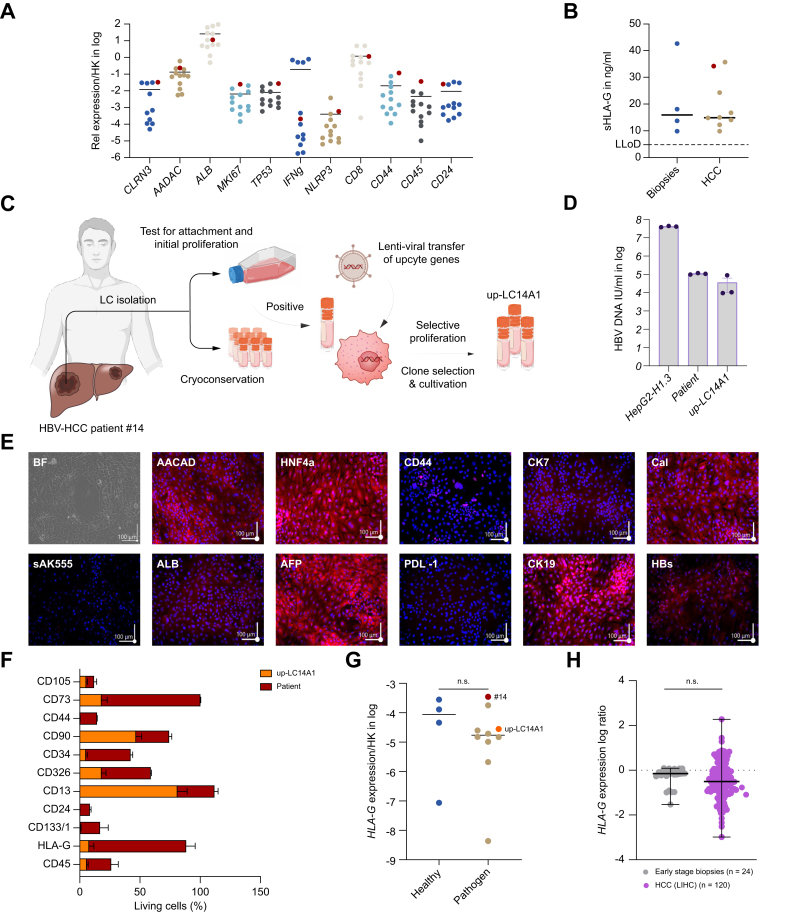
Generation and characterization of the patient-derived HBV-associated hepatocellular carcinoma (HCC) cell line up-LC14A1. (A) Generation and characterization of the patient-derived HBV-associated HCC cell line up-LC14A1. Gene expression of selected classifier genes in patient #14 relative to n = 13 patient-derived HCC samples. (B) Serum soluble HLA-G (sHLA-G) levels in early-stage biopsies (n = 4) and diagnosed HCC (n = 9; lower limit of detection 4.9 ng/ml); patient #14 highlighted in red. (C) Workflow for LC14 isolation and upcyte® cassette transfer, clonal selection, and establishment of up-LC14A1. (D) HBV DNA in supernatants and patient serum (day of resection) including HepG2-H1.3 control (n = 3). (E) Immunofluorescence markers; Hoechst nuclei; scale bar: 100 μm. (F) Flow cytometry marker frequencies (n = 3). (G) *HLA-G* expression (healthy n = 4; patient material n = 9; up-LC14A1). (H) TCGA *HLA-G* (biopsies n = 24; HCC n = 120).

We stratified the malignant material from patient #14 using diagnosed HCC tissue samples (in-house n13HCC cohort; n = 13; Table S1) for gene expression analysis. Across the plotted genes (Fig. 2A), patient #14 (red dot) aligned with the central distribution of the cohort, supporting its classification as representative HCC and providing a suitable basis for remodeling the immune-tolerogenic *HLA-G* haplotype. Next we analyzed serum samples from an overlapping cohort (n = 4 early-stage biopsies; n = 10 HCC; Table S1) for sHLA-G (Fig. 2B). Elevated sHLA-G levels were detected in patient #14 (red dot) compared with the dataset, consistent with the genetic phenotype.

To establish a functional patient-derived HCC cell line, parental liver cancer cells (p-LC14) were subjected to lentiviral transfer of the patented upcyte® gene cassette^15^ (Fig. 2C). This enabled sustained *in vitro* proliferation that would otherwise be lost after short-term culture. After selective expansion, up-LC14A1 cells demonstrated stable proliferative capacity under standard conditions with consistent doubling times (Fig. S2A). In line with previously generated primary-like cell lines, up-LC14A1 cells showed a higher median doubling time (43 h) than immortalized cell lines (20 h).^14^ In 2D culture, up-LC14A1 cells produced low levels of HBV DNA comparable to the patient’s serum viral load at resection (Fig. 2D), in contrast to the high-producing HepG2H1.3 cells.

To enhance its utility as an *in vitro* and *in vivo* model, up-LC14A1 cells were retrovirally transduced with a LeGoVector expressing green fluorescent protein (GFP) and LUC (Fig. S2B). After puromycin selection, 67.7% of cells expressed GFP (up-LC14A1_LUC; Fig. S2C), as determined by flow cytometry. Following passage without further puromycin selection, GFP expression approached 100% as confirmed by fluorescence microscopy (Fig. S2D). Subsequently, up-LC14A1_LUC cells were used to assess spheroid formation (Fig. S2E). After 4 days in spheroid-supporting U-bottom plates, cells organized into 3D networks that increased in size over time. This spheroid-forming capacity enables modeling of tumor progression and therapeutic responses in a physiologically relevant *in vitro* setting.

To position up-LC14A1 cells relative to commonly used immortalized HCC cell lines, we applied our standard immunofluorescence characterization panel (Fig. 2E and Fig. S2F).^14^ The up-LC14A1 cells showed high expression of CK19, calnexin, AFP, HNF4A, ACAD, vimentin, and actin. Low cytoplasmic expression was observed for hepatitis B surface protein, ALB, MICA/B, and CK7, with CD44 expression localized to the nucleus. The up-LC14A1 cells were negative for NTCP, EGFR, CD68, CD31, and PD-L1. Overall, up-LC14A1 cells retained the protein expression characteristics of p-LC14 cells as confirmed by flow cytometry (Fig. 2F), including surface presentation of HLA-G.

High *HLA-G* expression clinically correlates with reduced overall survival (Fig. S3G);^24^^,^^25^ therefore, we analyzed *HLA-G* expression in parental tissue and cells derived from patient #14 using the n13HCC cohort supplemented with healthy donor samples (Fig. 2G and Table S1). Overall, no significant differences were observed between healthy and diseased samples, consistent with global datasets showing similar *HLA-G* expression distributions across early and advanced liver diseases (Fig. 2H). However, patient #14 tissue and up-LC14A1 cells exhibited elevated *HLA-G* expression above the median of the pathogen group (Fig. 2G). These expression levels correspond to the previously observed sHLA-G pattern (Fig. 2B) and indicate stable HLA-G gene and protein expression. The highly progressive and migratory characteristics of up-LC14A1 cells, combined with their consistent expression of HLA-G, potentially mimic the immune-tolerogenic nature of HBV-related liver cancer.

### Transcriptomic profiling positions up-LC14A1 cells within the HCC landscape and reveals conserved hepatic and tumor-associated pathway activity

**Fig. 3:**
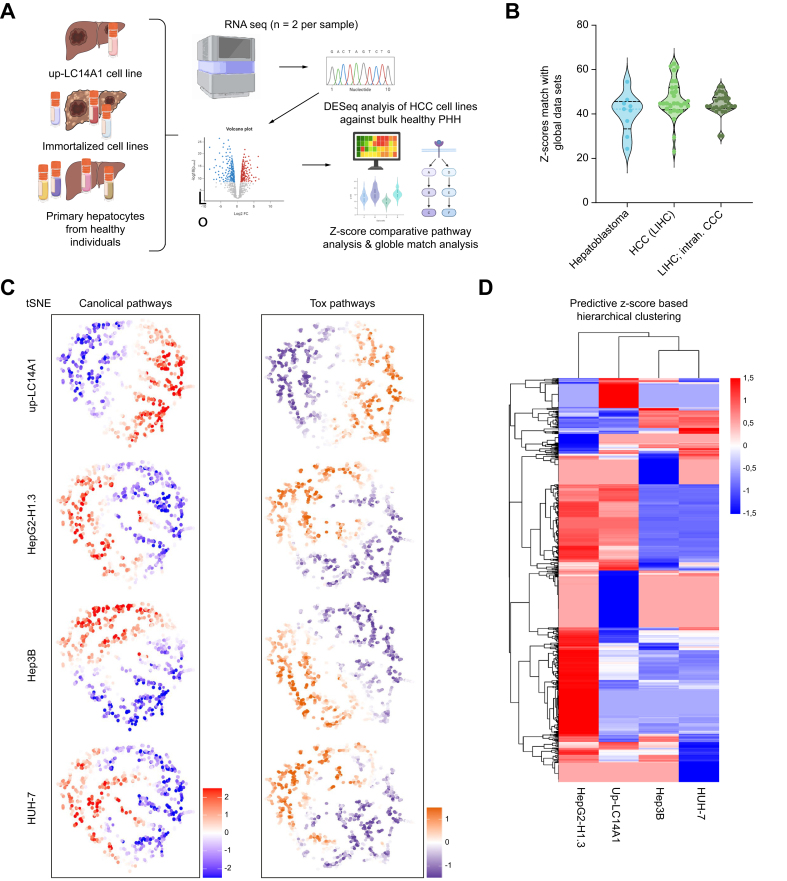
Transcriptomic classification of up-LC14A1 cells normalized against primary hepatocytes in a global context. (A) Transcriptomic classification of up-LC14A1 relative to primary human hepatocytes (PHH). RNA sequencing (RNA-seq) was performed for up-LC14A1, established HCC lines, and PHH (n = 2 technical replicates/sample). (B) Differential expression *vs*. PHH was followed by pathway and global similarity analyses using z-scores. Ingenuity Pathway Analysis (IPA)-based similarity of canonical/toxic pathways is shown as z-scores (values <10 excluded); violin plots summarize match scores across reference diagnoses. (C) Pathway activity was visualized by t-distributed stochastic neighbor embedding (t-SNE) for up-LC14A1, HepG2-H1.3, Hep3B, and HUH-7; colors indicate z-scored activity. (D) Hierarchical heatmap shows canonical pathway z-scores (red up, blue down; range -1.5 to 1.5).

We analyzed the transcriptomic profile of up-LC14A1 cells to assess their clinical relevance and similarity to commonly used immortalized HCC cell lines. RNA-seq analysis included up-LC14A1, HepG2H1.3, Hep3B, HUH7, and primary human hepatocytes (PHH) (Fig. 3A). Across global differentially expressed gene (DEG) datasets, up-LC14A1 cells showed a high z-score activation match with cholangiocarcinoma, HCC, and hepatoblastoma, indicating preservation of the underlying clinical disease (Fig. 3B). Variance-stabilized datasets from HCC cell lines and up-LC14A1 cells were subjected to DEG analysis using t-distributed stochastic neighbor embedding (Fig. 3C). Analyzed genes were associated with canonical signaling pathways as well as stress- and toxicity-related programs. Dimensionality reduction generated shared gene-level coordinates across cell lines, onto which relative expression differences were projected. Across canonical pathways, up-LC14A1 cells displayed activation patterns overlapping with established HCC lines, indicating preservation of core tumor-associated signaling programs. A similar distribution was observed for toxicity- and stress-related pathways, with up-LC14A1 mapping within the transcriptional space of reference HCC models rather than forming a distinct cluster. These findings indicate that up-LC14A1 recapitulates the global pathway architecture of HCC while retaining patient-specific transcriptional features.

For predictive pathway analysis, we evaluated normalized datasets using PHH as reference. Hierarchical clustering of canonical pathway activation (positive z-scores) and inhibition (negative z-scores) is shown in Fig. 3D. HUH7 and Hep3B exhibited high similarity in pathway regulation, whereas virus-producing HepG2H1.3 showed a more distinct profile. The pathway regulation pattern of up-LC14A1 partially overlapped with both HepG2H1.3 and Hep3B, suggesting infection-related pathway adaptations and similarities to HBV-associated HCC models. Principal component analysis (Fig. S3A and B) confirmed these relationships. HUH7 (FC43) and Hep3B (FC45) clustered closely, whereas HepG2H1.3 (FC44) displayed a distinct pathway regulation pattern. However, up-LC14A1 cells (FC33) occupied an intermediate position between non-HBV-producing lines (HUH7, Hep3B) and the HBV-producing HepG2H1.3 line. DEG analysis showed reduced *HLA-G* expression across all investigated cell lines; however, among the pathogenic lines, HepG2H1.3 exhibited the highest levels compared with PHH (Fig. S3C).

The individual gene regulation landscape of up-LC14A1 is illustrated in a volcano plot (Fig. S4A). Upregulated top genes (-log10 adjusted *p* value >200) were associated with biological regulation (six genes), cellular processes (15 genes), metabolic processes (eight genes), and responses to stimuli (six genes). Elevated expression of *HSPB1* and *EPAS1* suggested increased stress resistance, whereas *TIMP1, SERPINA1, GPNMB,* and *IL1R1* indicated an immune-modulatory phenotype. Notably, strong induction of *SERPINA1* supports the use of human AAT as an *in vivo* serum marker. Regulation of *ALAS1, COX4, and SCARB1* further indicated increased metabolic activity. The most prominently downregulated genes were associated with biological regulation (19 genes), cellular processes (34 genes), and metabolic processes (22 genes), including impaired mTOR and MAP kinase signaling. Detailed canonical pathway analysis (Fig. S4B), compared with PHH, showed downregulation of complement cascade, interferon (IFN) signaling, and mitochondrial translation pathways. In contrast, pathways related to protein ubiquitination, mitotic metaphase, neddylation, L1CAM interactions, and cell cycle checkpoints were upregulated.

### The up-LC14A1 cells better mimic HCC progression in xenograft mice than commonly used immortalized HCC cell lines

**Fig. 4:**
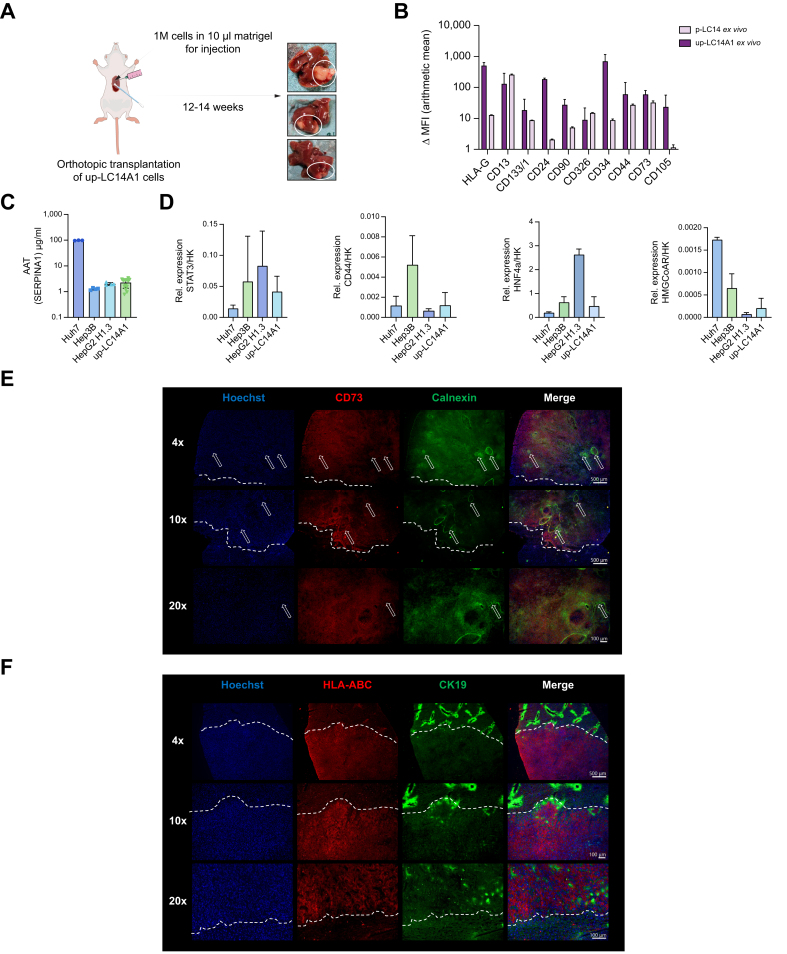
Serological and intrahepatic analysis of up-LC14A1 orthotopically transplanted immune-deficient mice. (A) Experimental design and representative chimeric livers after orthotopic transplantation of up-LC14A1 into immunodeficient mice. (B) The up-LC14A1 cells were re-isolated from repopulated livers (n = 3 mice) and analyzed by flow cytometry. Mean fluorescence intensity difference (ΔMFI) of indicated markers is shown for parental cells (p-LC14 *ex vivo*; n = 3 technical replicates) and engrafted up-LC14A1 cells (n = 3 biological replicates). (C) Serum alpha-1 antitrypsin (AAT) levels (μg/ml). (D) Intrahepatic gene expression of indicated genes (log_10_, normalized to housekeeping genes). (E, F) Immunofluorescence images showing tumor infiltration fronts (arrows) and tumor–host borders (lines) at different magnifications; scale bars indicated.

We evaluated the *in vivo* suitability of up-LC14A1 cells using orthotopic liver transplantation into immunodeficient mice (Fig. 4A). Representative images of chimeric livers are shown in the right panel. For comparison, HUH7, Hep3B, and HepG2H1.3 cells were transplanted under identical conditions. All three immortalized lines demonstrated strong tumorigenic persistence (Fig. S5A), with HUH7 showing pronounced necrotic liver areas. In contrast, although Hep3B and HepG2H1.3 induced fewer necrotic structures, animals reached ethical endpoints within 4 weeks owing to rapid deterioration of body condition. Mice transplanted with up-LC14A1 cells did not meet stop criteria before Week 12 (Fig. S5B), indicating slower progression without excessive health burden. This pattern supports a stemless tumorigenic growth leading to structured chimeric livers (Fig. S5C). Accordingly, up-LC14A1-transplanted mice were sacrificed after 12–14 weeks, and livers were analyzed by flow cytometry in comparison with p-LC14 cells (Fig. 4B). The livers repopulated with up-LC14A1 cells displayed a minor portion of ki67-and CD44-positive cancer cells (Fig. S6). After *in vivo* repopulation, up-LC14A1 cells express higher levels of HLA-G compared with the p-LC14 cells (Fig. 4B). Notably, markers that were absent in up-LC14A1 cells in conventional 2D culture, such as CD13, CD44, and CD24, were detected after expansion in the liver of immunodeficient mice. Taken together, we detected a higher cancer stem cell-like population in the up-LC14A1 cell line when compared with the p-LC14 cells isolated after resection and pretreatment. Serum analysis demonstrated highest levels of AAT (*SERPINA1*) in HUH7-transplanted mice, but low AAT levels in Hep3B-transplanted mice (Fig. 4C). Furthermore, AAT levels in HepG2H1.3- and up-LC14A1-transplanted mice were comparable. Considering tumor progression in the liver, up-LC14A1 induced moderate AAT secretion relative to immortalized HCC lines, whereas HUH7 produced markedly elevated levels consistent with aggressive, metastatic growth and the necrotic liver structures observed histologically (Fig. S5A). Intrahepatic RNA expression of *STAT3*, *CD44, HNF4A*, and *HMG-CoA reductase* (*HMG-CoAR*) showed pronounced heterogeneity (Fig. 4D). In line with others, we found high *STAT3* expression levels in the HBV-related cell lines Hep3B, HepG2H1.3, and up-LC14A1.^26^ Hep3B-transplanted livers displayed the highest *CD44* expression, whereas HepG2H1.3- and HUH7-transplanted livers predominantly displayed *HNF4A* and *HMG-CoAR* expression, respectively. In contrast, up-LC14A1-transplanted livers displayed moderate expression across all human-specific markers. Histological analysis confirmed structured infiltration and integration of up-LC14A1 cells into murine liver tissue, forming human cancer cell-derived chimeric livers (Fig. 4D). The up-LC14A1 cells were CD73 positive, consistent with moderate engraftment kinetics. Distinct infiltration fronts and clear tumor–host borders were visible (Fig. 4E); moreover, HLA-ABC-positive up-LC14A1 cells migrated through murine biliary epithelial channels (Fig. 4F), indicating defined routes of repopulation and progression.

### The up-LC14A1 cells displayed immune-tolerogenic dynamics, whereas immortalized HepG2H1.3 cells exhibited a static phenotype

**Fig. 5:**
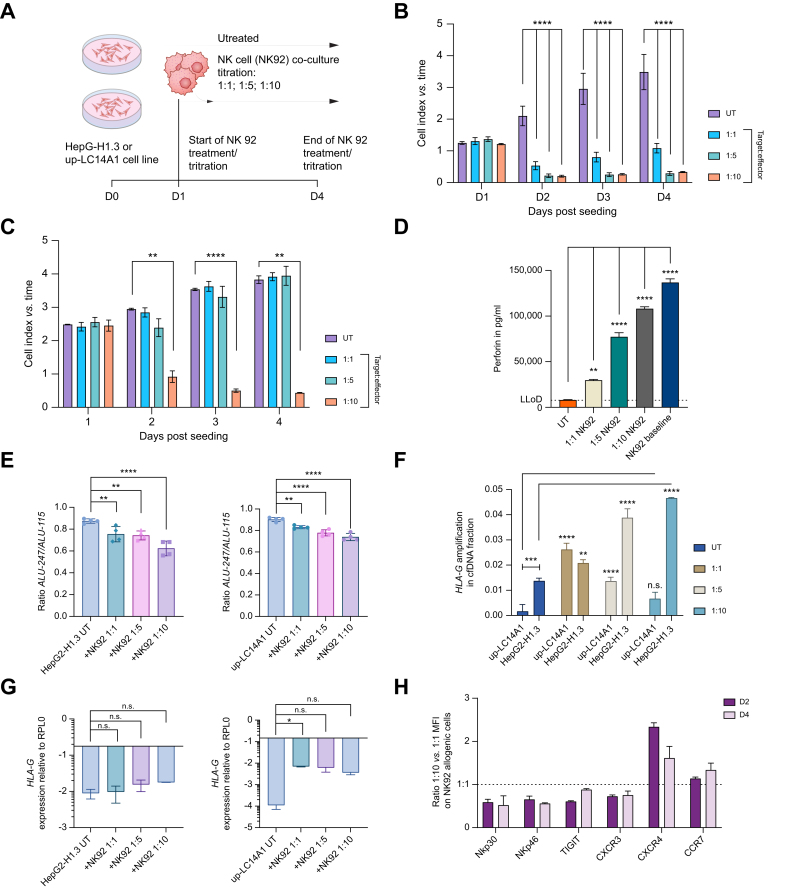
NK92 cell transfer reveals the adaptive immune-evasive phenotype of up-LC14A1 cells. (A) NK92 co-culture reveals adaptive immune evasion of up-LC14A1. Experimental scheme is shown. Longitudinal xCELLigence cell index is shown for (B) HepG2-H1.3 and (C) up-LC14A1 across indicated effector-to-target (E:T) ratios (n = 2 × 5 replicates/condition). (D) Perforin in supernatants is quantified (pg/ml, linear scale). (E) cfDNA fragmentation was assessed by *ALU-115/ALU-247* quantification and ratio analysis (n = 4), and (F) *HLA-G* gDNA in cell-free DNA (cfDNA) was quantified relative to RPL0 (n = 4). (G) Intracellular *HLA-G* mRNA after 24 h co-culture is shown (n = 4 biological replicates). (H) Flow cytometry compares marker fold-changes at high (1:10) *vs.* low (1:1) E:T on Days 2 and 4.

As determined by DEG analysis, HepG2H1.3 cells expressed HLA-G mRNA at higher levels than did HUH7, Hep3B, and up-LC14A1 cells. As demonstrated in a previous study,^14^ all three immortalized cell lines, as well as up-LC14A1 cells, present HLA-G protein on their surface (Fig. 2C). However, to determine immunotolerance induction, we used HepG2H1.3 cells. Their high HLA-G mRNA levels, consistent HBV production, DEG-based pathway clustering, and corresponding *HLA-G* 3′UTR UTR-1 haplotype make them a suitable model for a comparative investigation with up-LC14A1 cells.^27^ Therefore, immortalized NK92 cells were transferred into a 2D co-culture with different target-to-effector (T: E) ratios for 4 days (Fig. 5A). Consequently, HepG2H1.3 cells were significantly reduced in cell viability (Fig. 5B) regardless of the effector-to-target ratio, whereas up-LC14A1 cells displayed a clear adaption towards the immune NK92 cell treatment (Fig. 5C). On Day 2, 1:5 and 1:10 treatment regimens succeeded to reduce the proliferation rate and induced apoptosis; however, this effect was abrogated at 1:5 ratio on Day 4 totally. This finding contrasted with the cytotoxic active status of NK92 cells determined by the ratio, depending on linear detection of perforin in the supernatant (Fig. 5D). Moreover, in 1:1 T:E co-culture, NK92 cells were not sufficient to induce cytotoxicity. In contrast, NK92 cells effectively lysed HepG2H1.3 cells also in the 1:1 T:E setting. Nevertheless, over time in a 1:1 condition, HepG2H1.3 cells exhibited increased viability, likely because of the short lifespan of NK92 cells.

To elucidate the underlying cell death mechanism, we analyzed the occurrence of cfDNA-based long (247 bp) and short (115 bp) *ALU* fragments in the supernatant of indicated conditions (Fig. 5E). In both co-culture settings, we observed higher levels of short cfDNA fragments than long fragments, resulting in the low DNA integrity coefficients shown in Fig. 5E and providing further evidence that cell death was induced by apoptosis.^28^ In parallel, cfDNA was used for locus-specific analysis of *HLA-G* (Fig. 5F), revealing distinct release patterns between HepG2H1.3 and up-LC14A1. HepG2H1.3 displayed significantly higher basal *HLA-G*-positive cfDNA, whereas up-LC14A1 exerted low baseline levels, consistent with their distinct HLA-G regulatory states (*HLA-G* haplotypes). For HepG2H1.3 cultures, *HLA-G*-positive cfDNA levels correlated with overall cfDNA release and cytotoxicity, suggesting that locus-specific detection largely reflected passive DNA release associated with cell turnover (Fig. 5F). In contrast, under low cytotoxic pressure (1:1 effector-to-target ratio), up-LC14A1 cells exhibited a relative increase in *HLA-G* locus-positive cfDNA that could not be explained by apoptosis-associated DNA release alone. Notably, under conditions of stronger NK92-mediated cytotoxicity, *HLA-G*-positive cfDNA decreased despite increased apoptosis and reduced viability (Fig. 5C,F)*.* Taken together, our findings indicate that, for up-LC14A1 cells, the *HLA-G* locus cfDNA does not scale proportionally with cell death but instead reflects the release of *HLA-G*-containing DNA fragments from viable tumor cells under immune pressure, as part of tumor–immune communication.^29^

The intracellular gene expression pattern (Fig. 5G) confirmed this irregular pattern between HepG2H1.3 and the immune-tolerogenic up-LC14A1 cells. Interestingly, the low *HLA-G*-expressing up-LC14A1 cells increased expression by 20-fold within 24 h after the application of NK92 cells, whereas this dynamic was not observed in HepG2H1.3 cells. This finding of dynamic and static reactions to NK92 cell transfer was consistent with the expression patterns of both cell types and with the *HLA-G* haplotype of up-LC14A1 cells. Cytometric analysis of NK92 cells on Day 4 showed increased CXCR4 and CCR7 expression under 1:10 conditions, accompanied by downregulation of activation markers NKp30 and NKp46 (Fig. 5H). This pattern correlated with the enhanced killing observed at the higher effector-to-target ratio, indicating effective target cell elimination at 1:10 compared with 1:1 condition. Overall, NK92 cells shifted from a predominantly cytolytic toward a more migratory phenotype, demonstrating phenotypic adaptation of this immortalized NK cell line in response to dynamic, immune-tolerogenic target cell interactions.

### HLA-G knockdown reduces immune tolerance of up-LC14A1 cells and forces the switch to PDL-1 inhibition

**Fig. 6:**
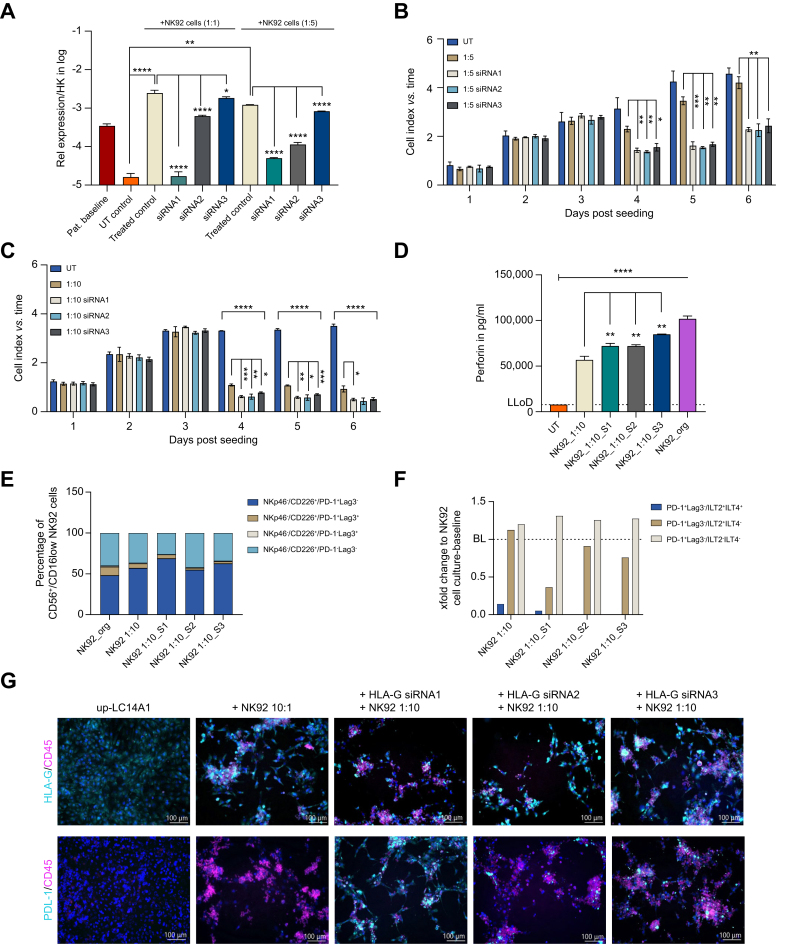
Functional effects of HLA-G silencing on NK92 cell responses toward up-LC14A1 cells. (A) Functional impact of HLA-G silencing on NK92 responses to up-LC14A1. HLA-G knockdown by siRNA in up-LC14A1 was quantified by normalized HLA-G mRNA under NK92 co-culture at indicated ratios (1:1, 1:5). (B, C) Longitudinal xCELLigence cell index of up-LC14A1 is shown for indicated conditions. (D) Perforin in supernatants after 72 h NK92 co-culture is shown (pg/ml; lower limit of detection indicated; percentages shown per condition). (E) Frequencies of CD16ˆdim and CD16low within CD56dim NK92 cells are shown. (F) PD-1/LAG-3 distribution is shown for CD56+/CD16low/NKp46−/CD226+ NK92 cells. (G) ILT2/ILT4 changes are shown as fold-change *vs.* baseline (BL) at 27 h (E:T 1:10). (H) Immunofluorescence at 48 h shows HLA-G or PD-L1 (aqua) with CD45 (purple) and Hoechst nuclei; scale 100 μm.

We assessed the immune regulatory role of HLA-G in up-LC14A1 cells by siRNA-mediated HLA-G silencing followed by NK92 co-culture (Fig. S7A). All three siRNAs significantly reduced *HLA-G* expression compared with control conditions, with siRNA1 producing the strongest knockdown (Fig. 6A). Longitudinal viability measurements confirmed NK92-mediated cytotoxic pressure and revealed reduced immune tolerance after HLA-G silencing (Fig. 6B,C). At effector-to-target ratios of 1:5 and 1:10, *HLA-G* knockdown consistently led to a stronger decline in cell index, accompanied by significantly increased perforin secretion, indicating enhanced cytolytic activation (Fig. 6D). Phenotypic profiling supported this effect. The CD56dim/CD16low compartment shifted toward a PD-1+/Lag3− composition across conditions, with the highest proportion of CD56dim/CD16low/NKp46−/CD226+/PD-1+/Lag3− cells observed after siRNA1 treatment, which also produced the strongest cytotoxic impact (Fig. 6B,C,E). Analysis of HLA-G receptors within this dominant NK92 subset showed that HLA-G reduction promoted a shift toward ILT2/ILT4 double-negative cells, whereas HLA-G-expressing conditions showed modest ILT2 induction (Fig. 6F). Effector–target interaction generally led to strong ILT4 downregulation, whereas siRNA1 caused the most pronounced decrease in ILT2 expression alongside the highest frequency of this activated subset. KIR2DL4 co-expression with ILT2 and/or ILT4 was detected under baseline conditions. Under siRNA1, 34% of ILT2/ILT4 double-positive and 8% of ILT2-positive cells retained KIR2DL4 (Fig. S7B). Immunofluorescence confirmed strong HLA-G protein induction after NK92 transfer, which was markedly reduced by siRNA treatment, most prominently with siRNA1 (Fig. 6G). Concomitantly, PD-L1 expression was strongly upregulated under HLA-G knockdown, with weaker induction in siRNA2 and siRNA3 conditions. Together, these data indicate that HLA-G suppression enhances NK92-mediated cytotoxicity while triggering compensatory PD-L1-mediated immune evasion, highlighting a dynamic switch between immune regulatory pathways.

### HLA-G is localized at target–effector contact sites, consistent with contact-dependent immune modulation

**Fig. 7:**
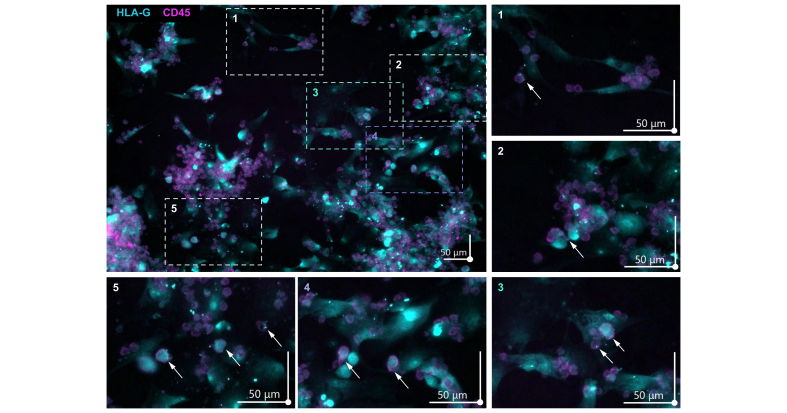
Membrane-bound HLA-G transferred on NK92 cells showing dynamic immune tolerance pathways of up-LC14A1 cells. Representative immunofluorescence images show HLA-G (cyan) on up-LC14A1 tumor cells and its redistribution to interacting NK92 cells identified by CD45 (magenta). Overview images indicate NK92 accumulation at HLA-G-expressing tumor surfaces; dashed boxes mark regions shown at higher magnification (panels 1–5). Arrows highlight tumor–NK contact sites and HLA-G signals on NK92 membranes after interaction, consistent with contact-dependent transfer. These findings support the formation of HLA-G-mediated immune regulatory interfaces compatible with local tolerance. Scale bars: 50 μm.

We analyzed the spatial relationship between HLA-G-expressing up-LC14A1 cells and NK92 cells by immunofluorescence microscopy (Fig. 7). Up-LC14A1 cells exhibited strong membranous and cytoplasmic HLA-G expression (cyan), whereas NK92 cells were identified by CD45 expression (purple). The co-culture resulted in the formation of dense cellular clusters in which NK92 cells accumulated around HLA-G-presenting up-LC14A1 cells, pointing to active immune–tumor cell interaction. In high-magnification views,[1], [2], [3], [4], [5] multiple direct contact zones between NK92 cells and up-LC14A1 cells are highlighted. At the interfaces, HLA-G signal was frequently concentrated at the contact sites and, in several instances, appeared at the surface of adjacent CD45^+^ NK92 cells (white arrows). These observations suggest that HLA-G is actively engaged at the immune synapse and redistributed during these target–effector interactions.

## Discussion

Chronic HBV infection is a major global driver of HCC, accounting for up to 50% of cases and representing a key factor in inflammation-associated hepatocarcinogenesis.[1], [2], [3]^,^^5^^,^^30^ In this setting, the non-classical MHC class I molecule HLA-G has emerged as a potent immune checkpoint that suppresses cytotoxic lymphocyte activity through inhibitory receptors ILT2 and ILT4 on NK cells, T cells, and myeloid cells.^6^^,^^7^^,^^9^ By attenuating cytotoxicity, cytokine release, and immune synapse formation, HLA-G promotes immune tolerance within the tumor microenvironment.^6^^,^^7^^,^^9^^,^^31^ In chronic HBV infection, miR-152 downregulation increases HLA-G expression, impairing NK-cell responses and linking viral persistence to HLA-G-mediated immune escape.^10^

HLA-G can be present either in mHLA-G or sHLA-G form.^7^ In the clinical setting, elevated sHLA-G levels predict poor overall survival in patients with virus-induced HCCs, alcohol-associated liver disease (ALD), and metabolic dysfunction-associated steatotic liver disease/steatohepatitis (MASLD/MASH) entities.^32^ Moreover, serum sHLA-G levels are associated with clinical phases of chronic HBV infection and correlate with viral persistence and immune tolerance, supporting the role of HLA-G in shaping the host-virus equilibrium.^6^^,^^8^^,^[33], [34], [35] More recently, combined analyses of HLA-G expression and regulatory polymorphisms, including 3′UTR variants, have linked high sHLA-G levels to adverse clinical outcomes across viral and metabolic HCC entities.^32^^,^^36^

In the present study, we established a patient-derived HBV-HCC model (up-LC14A1) that retains key features of the parental tumor. Stratification of parental tumor tissue and serum reveals typical HCC characteristics. Spatially resolved WES of the parental tumor tissue revealed a clonally coherent mutational landscape enriched for antigen presentation and immune-interaction genes. Notably, the malignant tissue carried a regulatory *HLA-G* 3′UTR-2 haplotype, which is reportedly associated with HBV susceptibility and immune-tolerogenic processes,^11^^,^[20], [21], [22] and therefore appears as driver of immune modulation in this patient. In this study, we showed that up-LC14A1 cells capture a stable, patient-specific immunotolerogenic configuration rather than a stochastic adaptation. Importantly, HLA-G expression is known to be influenced by both genetic variants and inflammatory cues, highlighting the interplay between clonally encoded predisposition and inducible immune regulation.^6^^,^^7^

Phenotypically, up-LC14A1 cells present hepatobiliary lineage markers, including CK19, AFP, and HNF4A, confirming their origin from HBV-associated liver cancer. They also express HLA-G and CD73, pointing to an immune-evasive and progenitor-like phenotype. CD73 is a key component of the adenosine pathway, generating extracellular adenosine that suppresses NK-cell activity and promotes tumor–immune escape.^7^^,^^37^ Expression of CD73 in HCC has been associated with stem-like properties, therapy resistance, and immunosuppression, supporting the relevance of this phenotype for disease progression and treatment resistance.^7^ By orthotopic transplantation, up-LC14A1 cells displayed gradual integration into the murine liver and non-necrotic tumor growth, in contrast to the rapid, often necrotic expansion of commonly used immortalized HCC cell lines. This slower progression resulted in the formation of chimeric livers and prolonged the survival of transplanted animals, indicating a more gradual tumor expansion within this experimental setting.

Transcriptomic profiling placed up-LC14A1 cells between HBV-producing (HepG2H1.3) and non-producing (Hep3B, HUH7) HCC lines, consistent with a hybrid, virus-associated tumor state. Functionally, the model showed dynamic immune tolerance: under NK92 pressure, HLA-G was rapidly induced and enriched at tumor–immune contact sites. Its presence at effector-cell interfaces aligns with reported trogocytosis-mediated transfer of HLA-G from tumor to NK92 cells, which can convert cytotoxic lymphocytes into regulatory cells and promote immune escape.[38], [39], [40] This spatial redistribution supports contact-dependent suppression within the tumor–immune synapse.^7^ We further detected *HLA-G*-containing DNA fragments released from viable tumor cells under immune pressure, suggesting a role in tumor–immune communication.^29^

Silencing HLA-G restored NK92 cell cytotoxicity and triggered compensatory PD-L1 upregulation, indicating that HLA-G is part of the hierarchical checkpoint network.^41^ Interconnected immune checkpoint pathways have been described across multiple tumor types, in which inhibition of one suppressive axis results in the activation of alternative pathways to maintain immune escape.^41^^,^^42^ Interferon-γ (IFN-γ) signaling from activated NK or T cells is a plausible mechanism for PD-L1 induction in this setting, as IFN-γ is a canonical driver of PD-L1 expression on tumor cells and contributes to adaptive immune resistance.^43^ These observations support a model in which HLA-G functions as a central but dynamic node within an adaptive immune regulatory circuit rather than as a static inhibitory molecule.^44^

In summary, our data place HLA-G within a clonally encoded yet dynamically regulated immune tolerance program in HBV-associated HCC. The up-LC14A1 model provides a platform to dissect how genomic background, viral context, and immune pressure jointly shape immune escape and therapy resistance—a key challenge for emerging HCC immunotherapies.^1^^,^^3^

A limitation is the use of upcyte® technology, which confers proliferative capacity while preserving primary-like features but may alter regulatory pathways.^15^^,^^16^ Although we confirm parental characteristics to a relevant extent, future studies using patient-derived NK and T cells are required to validate and extend HLA-G-mediated tolerance mechanisms.

Overall, our findings support a role of HLA-G in immune evasion through both genetically encoded predisposition and inducible regulation under immune pressure. The up-LC14A1 model provides an experimental platform to investigate how genomic background, viral context, and immune pressure contribute to immune escape in HBV-associated HCC.

## Abbreviations

2D, two-dimensional; 3D, three-dimensional; AAT, alpha-1 antitrypsin; AltAF, alternate allele fraction; cfDNA, cell-free DNA; DEG, differentially expressed gene; GFP, green fluorescent protein; HCC, hepatocellular carcinoma; HLA, human leukocyte antigen; ICI, immune checkpoint inhibitor; IPA, Ingenuity Pathway Analysis; LC14/(p-LC14), (parental) liver cancer cells 14; LeGO, LeGO vector system; LUC, luciferase; MHC, major histocompatibility complex; mHLA-G, membrane-bound HLA-G; NK cells, natural killer cells; PD-1, programmed cell death protein 1; PD-L1, programmed death-ligand 1; PHH, primary human hepatocytes; RNA-seq, RNA sequencing; sHLA-G, soluble HLA-G; SNV, single-nucleotide variant; t-SNE, t-distributed stochastic neighbor embedding; TKI, tyrosine kinase inhibitor; up-LC14A1, upcyte-modified LC14A1; VAF, variant allele frequency; WES, whole-exome sequencing.

## Authors’ contributions

JK initiated and supervised the research study; JK, LS, SK, PVL, and MD designed the experiments; AH and KS supervised the collection of human samples; JK, LS, GM, NH, MV, SS, and TV conducted experiments and acquired data; JK, LS, SS, PVL, WD analyzed data; JK, WD, JHK, SK, SL, and MD wrote the manuscript. All authors had accessed to the study data, discussed the data, and corrected the manuscript. All authors reviewed and approved the final manuscript.

## Data availability

The datasets generated and analyzed during this study, including transcriptomic data and functional assay results, are available from the corresponding author upon reasonable request. The up-LC14A1 cell line can be made available to academic researchers for non-commercial use under a material transfer agreement. Additional methodological details are provided in the supplementary materials, and custom protocols are available upon request.

## Financial support

The German Research Foundation (DFG) funded the study with a grant to JK (KA 5390/2-1) and to MD (CRC 1700; 530990199). All funding sources supporting the work are acknowledged, and authors have nothing to disclose.

## Conflicts of interest

The authors have no conflicts of interest to declare. All co-authors have seen and agree with the contents of the manuscript.

Please refer to the accompanying ICMJE disclosure forms for further details.

## Supplementary data

Multimedia compoennt 1

Multimedia compoennt 2

Multimedia compoennt 3
