# Supplementary material for: Targeting a tolerogenic HLA-G genotype to tackle immune evasion and adaptive resistance in HBV-driven HCC
Source: JHEP Rep. 2026 May 8;8(8):101891. doi: 10.1016/j.jhepr.2026.101891 (PMC13352049; doi:10.1016/j.jhepr.2026.101891)
Supplement: Multimedia compoennt 1 [file mmc1.pdf]

Targeting a tolerogenic HLA-G genotype to tackle immune evasion and adaptive resistance in HBV-driven HCC

Janine Kah, Lisa Staffeldt, Svenja Stefanski, Natalie Herzog, Gregor Mattert, Tassilo Volz, Maximilian Voß, Kornelius Schulze, Asmus Heumann, Werner Dammermann, Sarah Kammerer, Jan-Heiner Küpper, Pablo Villavicencio-Lorini, Maura Dandri, Stefan Lüth

Table of contents

Supplementary materials and methods……………………………………………………2

Supplementary figures………………………………………………………………………..13

Supplementary tables…………………………………………………………………………23

Supplementary references……………………………………………………………………27

1) Supplementary methods

Sample collection, processing and ethics. Human tissue and blood samples were collected, isolated, preserved, and ethically approved as described previously (1, 2). Clinical data was provided in **table S1**. HLA-A typing was performed for patient- and healthy donor-derived tissue and blood cells as described previously (1). The studies were approved by the Ethical Review Committee of the Ärztekammer Hamburg (PV-3578). Handling of human material was performed in accordance with national guidelines and the 1975 Declaration of Helsinki. All animal experiments were conducted in accordance with the European Communities Council Directive (86/EEC) and were approved by the City of Hamburg, Germany (N056/2020). Generation, breeding, and housing of the mice, as well as surgery, were conducted as described previously (2).

up-LC14 generation and long-term storage. Patient-derived HCC cells were isolated as described previously (2) and used to generate proliferation-competent cultures. Cells were genetically modified to obtain a prolonged lifespan as described previously for primary hepatocytes (3, 4). Briefly, cells were resuspended in HCC medium consisting of Dulbecco’s Modified Eagle Medium (DMEM; Bio & Sell, 90537 Feucht, Germany) supplemented with 10% fetal calf serum (FCS; Bio & Sell, 90537 Feucht, Germany), 2 mM L-glutamine (Bio & Sell, Germany), and 1% Pen/Strep (Biowest, 49340 Nuaillé, France), centrifuged at 60 × g for 5 min, and resuspended in fresh HCC medium. Per well, 0.24 × 10^6 cells were seeded in collagen-coated 24-well plates (Sarstedt AG & Co. KG, 51588 Nümbrecht, Germany). The following day, cells were transduced with viral particles containing proliferation-inducing genes in HCC medium without Pen/Strep. Recombinant lentiviruses were generated using the ViraPower™ Lentiviral Expression System (Thermo

Fisher Scientific, Waltham, MA 02451, USA) according to the manufacturer's protocol. Virus-containing supernatants were concentrated 20-fold using a filtration system (Vivaspin® 20; Sartorius) and applied directly for infection. Medium was changed every 2–3 days. After ~6 weeks, proliferating liver cancer cells were transferred into 6-well plates (Sarstedt AG & Co. KG, 51588 Nümbrecht, Germany) for expansion in optimized HCC medium consisting of DMEM/Ham's F12 (Bio & Sell, 90537 Feucht, Germany; 1:1) supplemented with 10% FCS, 2 mM L-glutamine, and 1% Pen/Strep. For cryopreservation, ≥1 × 10^6 cells/mL were frozen in 70% DMEM/Ham's F12 (Bio & Sell, 90537 Feucht, Germany), 20% FCS, and 10% DMSO (Sigma, St. Louis, MO 63118, USA) using a Corning™ CoolCell™ freezing container (Thermo Fisher Scientific, Waltham, MA 02451, USA) prior to storage in liquid nitrogen.

HCC cell cultivation, genetic modification and spheroid formation. After thawing, up-LC14 cells were passaged upon reaching 70–80% confluence in Advanced DMEM/F-12 containing L-glutamine and glucose supplemented with 1% P/S and 10% Gibco fetal bovine serum (FBS; all from Thermo Fisher Scientific, Waltham, MA 02451, USA). Stable integration of the LeGO-iG2-Puro+-Luc2 construct by lentiviral transduction was performed as described previously (2). Immortalized HUH-7, Hep3B, and HepG2-H1.3 cells were maintained as described previously (5) in DMEM containing L-glutamine and glucose supplemented with 1% P/S and 10% Gibco FBS (Thermo Fisher Scientific, Waltham, MA 02451, USA). Cells were detached using Detachin™ Cell Detachment Solution (AMSBIO, Abingdon, UK) and cultured at 37 °C and 5% CO_2.

For 2D experiments, HCC and up-LC14 cell lines were seeded in 96- or 24-well plates at 10,000 or 50,000 cells/well using the corresponding passage medium and allowed to

adhere for 24 h prior to treatment. For 3D spheroid formation, 10,000–30,000 cells/well of luciferase-transduced up-LC14 cells were seeded on BIOFLOAT™ cell culture plates in Advanced DMEM/F-12 containing L-glutamine and glucose supplemented with 1% P/S and 10% Gibco FBS (Thermo Fisher Scientific, Waltham, MA 02451, USA) and maintained for 3 weeks to follow spheroid formation. The LeGO-iG2-Puro+-Luc2 construct (3rd generation HIV1-derived self-inactivating vector) enabled visualization. During spheroid formation, images were acquired automatically using the BX-780 microscope (Keyence, Osaka, 533-0033 Japan). HLA-G knock-down was performed using the human pre-designed siRNA Sets (15062) directed against the Gene ID: 3135 (cat#: HY-RS06217, MCE, NJ 08852, USA) following the manufacturer´s instructions.

NK92 culture and *in-vitro* treatment. For *in-vitro* experiments, enriched NK92 cells were thawed and cultured in MEM α medium (Gibco, Thermo Fisher, Massachusetts 02142, USA) supplemented with 12.5% horse serum (Gibco, Thermo Fisher, Massachusetts 02142, USA), 12.5% fetal bovine serum (Thermo Fisher Scientific, Massachusetts 02142, USA), 500 IU/mL rhIL-2 (Sartorius, Göttingen, Germany) and human IL-2 IS (Miltenyi Biotec, Bergisch Gladbach, Germany). After sufficient expansion, NK92 cells were counted, washed by centrifugation at 350 g, and transferred to target cells at effector-to-target ratios of 1:1, 1:5, and 1:10. Ratios have been selected based on preliminary titration experiments to ensure robust and reproducible detection of NK92 cell–mediated killing. Co-cultures were maintained for 72 hours to capture cumulative cytotoxic effects and functional adaptation of NK92 cells beyond the initial contact phase. Shorter incubation periods (e.g., 24 hours) primarily reflect early activation and transient killing events, whereas 72 hours enables assessment of sustained effector activity. At indicated time points, supernatants and/or NK92 cells were collected for downstream analyses. Viability

was monitored by xCELLigence analysis (see “Doubling time assay”). Supernatants were used for cfDNA isolation. NK92 cells and target cells were analyzed using TaqMan single assays (**table S2**) following oligonucleotide isolation or by flow cytometry (**table S3**).

Doubling time assay (xCELLigence). Cell doubling was determined using the xCELLigence RTCA SP device (ACEA Biosciences, 92121 San Diego, USA). up-LC14 cells were seeded at 10,000 cells/well in an E-Plate 16 (ACEA Biosciences) and cultured for 72 h in up-LC14 culture medium. Doubling time was calculated from n = 5 technical replicates using xCELLigence RTCA Software v2.0 during the exponential growth phase.

Isolation of oligonucleotides. For RNA isolation from cultured cells, HCC cell lines were seeded into 24-well plates at 50,000 cells/well and harvested at ≥80% confluence. For RNA isolation after immune cell transfer, HCC cell lines were seeded as described above and treated with the indicated immune cells for 24 h; four wells per condition were pooled in RNA lysis buffer and stored at −80 °C until extraction. For isolation of RNA and DNA from chimeric or human liver samples, representative tumorigenic tissue was selected and 10–30 mg processed as described previously (1). RNA was isolated using the RNeasy Micro™ (cell lines) and RNeasy Mini™ (tissue) RNA purification kits (Qiagen, 40724 Hilden, Germany) according to the manufacturer’s instructions (6).

TaqMan-based gene expression analysis. Gene expression was quantified by two-step PCR. cDNA synthesis was performed using the MMLV Reverse Transcriptase™ 1st-Strand cDNA Synthesis Kit (Lucigen, Middleton, Wisconsin 53562, USA) according to the manufacturer’s instructions. Human-specific primer/probe sets from the TaqMan Gene Expression Assay System (Life Technologies, 92008 Carlsbad, California, USA; Table S1) were used (**table S2**). Samples were analyzed on the QuantStudio 7™ Real-Time PCR

System (Life Technologies, 92008 Carlsbad, California, USA) using the Fast protocol for 96-well plates (95 °C for 20 s; 40 cycles of 95 °C for 15 s and 60 °C for 20 s; cooling at 20 °C for 2 min). TaqMan Fast Advanced Master Mix (5×) (Life Technologies, 92008 Carlsbad, California, USA) was used according to the manufacturer's instructions. Data were analyzed using Design & Analysis Software v2.8.0 (Thermo Fisher Scientific, Massachusetts, USA). Gene expression was normalized to human housekeeping genes ribosomal protein L0 (RPL0), RPL30, and GAPDH.

Whole-exome sequencing and variant analysis. Genomic DNA was isolated from spatially separated tumor regions as described previously (6). DNA quantity and integrity were assessed by Qubit fluorometric quantification and fragment analysis (Agilent TapeStation). For each sample, 50 ng genomic DNA was used for whole-exome library preparation with hybrid-capture enrichment (Twist Precision Exome DX kit), followed by amplification and quality control. Paired-end sequencing (2 × 150 bp) was performed on an Illumina platform to obtain a mean on-target coverage ≥100fold. Demultiplexing was performed using Illumina software, and data were exported in bcl2/fastq format.

Secondary analysis was performed using IVD-CE Varvis® software v2.7.3, including adapter trimming and quality filtering prior to alignment to GRCh38/hg38. Alignments were sorted, duplicates marked, and base quality recalibration and indel realignment performed; base calling reached Q30 > 90%. SNVs and small indels were identified using a somatic variant-calling workflow. Variant call files were filtered for quality parameters (including read depth, mapping quality, and strand bias), retaining variants with ≥10 supporting reads and AltAF ≥0.05. Analyses were restricted to coding and flanking intronic regions, 5′/3′UTRs within the captured exome, and spiked-in mitochondrial regions.

Tertiary analysis was performed to predict HIGH or MODERATE functional impact. Varvis® output included genomic coordinates, quality/coverage metrics, functional annotations, and database annotations (dbSNP, ClinVar, OMIM). Variant classification followed ACMG/AMP guidelines.

Downstream analyses were performed in R (v4.x) using RStudio. VCF files were imported and converted to tabular format for filtering and annotation-based analyses. Variants were filtered to retain putative functional events (non-synonymous, predicted HIGH or MODERATE impact, VAF ≥0.05, ≥10 supporting reads). Gene-level variant counts were calculated for tumor center and margin, and top variant-enriched genes were identified by total variant number per gene. Visualizations were generated using ggplot2, dplyr, and tidyr. VAF distributions were plotted as density plots. Variant overlaps were visualized using Venn diagrams (VennDiagram or ggVennDiagram). Annotation-based summaries (dbSNP, ClinVar) were calculated from annotated tables and visualized as stacked bar charts. Locus-specific variant distributions for immune-relevant genes were extracted and plotted in ggplot2.

RNA sequencing and differential gene expression analysis. Total RNA was extracted from primary hepatocytes, the up-LC14A1 primary-like cell line, and immortalized HCC lines (HepG2H1.3, Huh7, Hep3B) as described previously (1). RNA quality and quantity were assessed using a NanoDrop spectrophotometer (Thermo Fisher Scientific, 76185 Karlsruhe, Germany) and an Agilent Bioanalyzer 2100 (Agilent Technologies, 76337 Waldbronn, Germany) with High Sensitivity DNA Chips (Cat. 5067-4626). Samples with RIN > 7.0 were used. Libraries were prepared using the TruSeq Stranded mRNA Kit (Illumina, 81669 Munich, Germany) according to the manufacturer’s instructions (mRNA

enrichment, fragmentation, cDNA synthesis, adapter ligation, PCR amplification). Libraries were quantified using a Qubit fluorometer (Thermo Fisher Scientific, 76185 Karlsruhe, Germany) with the Qubit 1X dsDNA HS Assay Kit (Q33230) and assessed for size distribution using the Agilent Bioanalyzer 2100 (Agilent Technologies, 76337 Waldbronn, Germany). Sequencing was performed on an Illumina NextSeq 1000/2000 platform generating 200 bp paired-end reads. Technical replicates ($n = 2$) were included per sample. Reads were trimmed using Trimmomatic, aligned to GRCh38 using STAR, and quantified using featureCounts. Differential gene expression was analyzed using DESeq2; significance thresholds were adjusted $p < 0.05$ and $|log2FC| > 2$. DEGs were visualized using volcano plots, z-score-based hierarchical heatmaps, and match z-score plots using IPA, GraphPad, and R-based tools [19–21]. For t-SNE visualization, raw count matrices of up-LC14A1, HepG2-H1.3, Hep3B, and HUH-7 were processed in RStudio using DESeq2. Counts were variance-stabilized (VST), mapped from Ensembl IDs to gene symbols, and summarized at gene level. Canonical pathway genes were obtained from the MSigDB C2:CP collection, while toxicity- and stress-related genes were selected from MSigDB via keyword filtering (xenobiotic response, oxidative stress, hypoxia, DNA damage, inflammatory signaling). Expression matrices of selected pathway genes were z-score normalized per gene and subjected to t-SNE using the Rtsne package (perplexity = 15). A shared embedding was generated at gene level, and cell line–specific pathway activity was visualized by calculating gene-wise expression differences for each cell line versus the remaining lines, z-transforming these values, and overlaying them onto the shared t-SNE coordinates. Analyses and visualizations were performed in R using DESeq2, Rtsne, msigdbr, and ggplot2.

Immunofluorescence staining. For *in-vitro* histological characterization of up-LC14 cells, 10,000 cells were seeded into 96-well plates and fixed at ~80% confluence with 4% PFA for 10 min after washing with 1× PBS. Cryosections (5 µm) were prepared from mouse-derived tissues representing tumorigenic and non-tumorigenic areas, stored at −80 °C, and fixed with 4% PFA for 10 min prior to staining. After fixation, mouse tissue sections and up-LC14 cells were stained by immunofluorescence using primary antibodies (**table S5**) with overnight incubation. Signals were detected using Alexa 488- (green) or Alexa 555- (red) labeled secondary antibodies (Invitrogen, Darmstadt, Germany) after 1 h incubation. Nuclei were stained with Hoechst 33258 (Invitrogen, Eugene, OR 97402, USA) and mounting was performed using mounting medium (Dako Deutschland GmbH, 22769 Hamburg, Germany). Imaging was performed using fluorescence microscopy (BZ-9000 and BX-780, Keyence, Osaka, 533-0033 Japan). Tissue images were acquired manually using consistent exposure times for comparable material/antibody settings. up-LC14 cell images were acquired automatically using identical magnification and exposure for the same antibodies. Bright-field images were acquired at matching magnification to phase contrast.

Flow cytometry. Flow cytometry analyses of human- and mouse-derived HCC cells were performed as previously described (2); antibodies are listed in **table S4**. NK92 cells were analyzed at indicated time points after treatment using a SymphonyA3™ flow cytometer (Becton Dickinson, New Jersey 07417, USA) and stained using antibodies listed in Table S2. Antibodies were incubated for 30 min at room temperature protected from light. Prior to staining, cells were incubated with Human BD Fc Block™ (Becton Dickinson, New Jersey 07417, USA). Gates were defined using fluorescence minus one (FMO) controls.

ELISA-based protein measurements. Human sHLA-G levels in human serum and cell culture supernatants were measured using the Human Major Histocompatibility Complex Class I G ELISA Kit (Thermo Fisher, Waltham, MA 02451, USA) according to the manufacturer's instructions; samples were diluted 1:1–1:3. Human alpha-1 antitrypsin (AAT) levels in murine serum were quantified using the Human alpha 1 Anti-trypsin ELISA Kit (abcam, Cambridge, MA 02139, USA) according to the manufacturer's instructions; murine serum was diluted 1:2500. Human perforin levels in cell culture supernatants were measured using ELISA Flex: Human Perforin (HRP) (Mabtech AB, Nacka Strand, 13152, Sweden) according to the manufacturer's instructions. Assay Diluent (5×; Cat. 421203) and TMB Substrate Set (Cat. 421101) were purchased from BioLegend. Samples were diluted 1:100.

HBV DNA quantification. HBV DNA was extracted from serum and cell culture supernatants using the QIAamp MinElute™ Virus Spin Kit (Qiagen, Hilden, Germany). Quantification was performed by TaqMan PCR using an HBV-specific probe (**table S2**). Cloned HBV DNA references were amplified in parallel to generate a standard curve.

cfDNA isolation and fragment analysis. For cfDNA isolation, cell culture supernatants were centrifuged for 10 min at 1000 x g and stored at −20 °C. cfDNA was isolated using the MagMAX™ cfDNA Isolation Kit (Thermo Fisher, Waltham, MA 02451, USA) according to the manufacturer's instructions. Samples were diluted 1:2 with water before denaturation with protein kinase K and sodium dodecyl sulphate (SDS) for 20 min at 60 °C. Samples were incubated with MagMAX™ Cell Free DNA Lysis/Binding Solution and MagMAX™ Cell Free DNA Magnetic Beads for 20 min, chilled on ice for 5 min, and cfDNA was bead-bound. Bound fragments were washed with MagMAX™ Cell Free DNA Wash

Solution and 80% ethanol. After centrifugation, supernatants were removed and cfDNA was eluted using MagMAX™ Cell Free DNA Elution Solution into fresh microcentrifuge tubes. For ALU-based fragment analysis, cfDNA qPCR reactions were performed in 14 µl containing 1 µl template, 7.5 µl PowerUp™ SYBR™ Green Master Mix (Thermo Fisher, Waltham, MA 02451, USA), 4.5 µl RNase-free water, and 1 µl primer mix (**table S6**). Short and long ALU fragments (ALU-115 and ALU-247 (7)) were amplified using the QuantStudio™ 7 Pro Real-Time PCR System (Thermo Fisher Scientific, Massachusetts, USA) with cycling conditions of 95 °C for 2 min, followed by 40 cycles of 95 °C for 15 s and 60 °C for 1 min, and cooling at 20 °C for 2 min. Data were analyzed using Design & Analysis Software v2.8.0 (Thermo Fisher Scientific, Massachusetts, USA). For locus-specific analysis of HLA-G, the TaqMan HLA-G assay Hs03045108_m1 (amplicon ~109 bp; designed for transcript detection across the exon 5–6 boundary) was applied using the same workflow.

Statistics and sample size. Graph design and statistical analyses were performed using GraphPad Prism v10 (GraphPad Software, Inc., La Jolla, CA, USA). For cell viability measurements, n = 5 technical replicates were used per group and analyzed by two-way ANOVA. RNA-based DEG and WES analyses included n = 2 technical replicates from HUH-7, Hep3B, HepG2H1.3, up-LC14A1 cell lines, patient material and healthy hepatocytes. HBV DNA titers were measured in n = 3 technical replicates. For the cytometric analysis shown in Fig. 3B, cells from n = 3 chimeric mice were pooled after isolation and measured in n = 2 technical replicates; patient-derived isolated liver cancer cells and cell lines were analyzed accordingly. For AAT endpoint measurement, serum was collected from n = 3 (HUH7), n = 4 (Hep3B), n = 4 (HepG2.1.3), and n = 14 (up-LC14A1) mice. For sHLA-G detection, sample sizes are provided in the corresponding

figure legends. Perforin was measured from n = 2 technical replicates per condition. For cfDNA isolation, n = 4 technical replicates were used and analyzed using one-way ANOVA with multiple comparisons. For gene expression analyses of cell culture samples, n = 3 technical replicates were used. Delta MFI was calculated from n = 2 technical replicates at the indicated time points. For comparisons involving more than two conditions, one-way ANOVA was applied; two-condition comparisons used Student's t-test. Statistical outputs are shown where applicable and significance is indicated as $^{*}p < 0.05$; $^{**}p \leq 0.01$; $^{***}p \leq 0.001$; $^{****}p \leq 0.0001$.

2) Supplementary figures

Figure S1

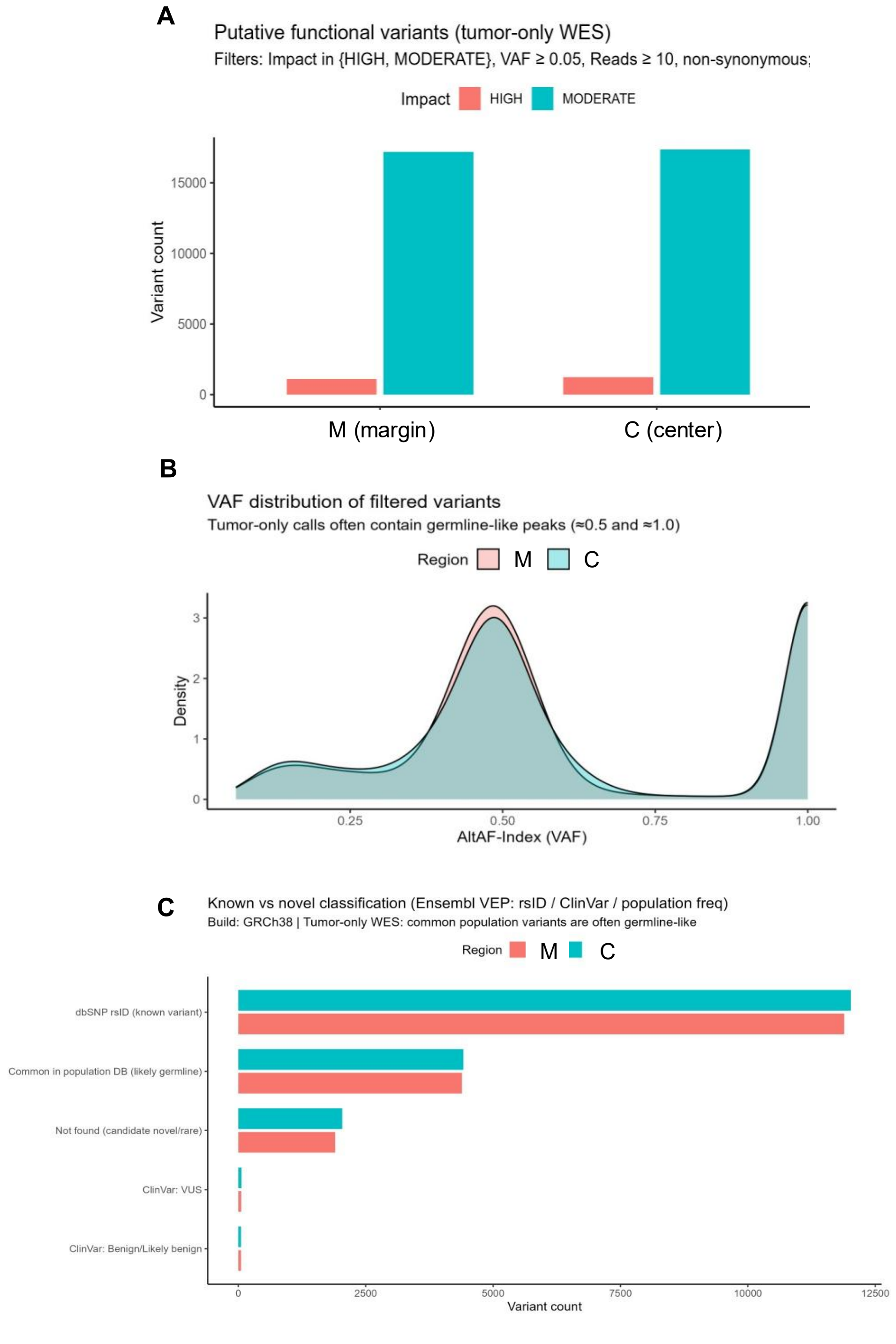

Fig. S1. Summary of patients WES-based variant characteristics. (**A**) Number of putative functional variants detected by whole-exome sequencing. Variants were filtered for predicted impact (HIGH or MODERATE), variant allele frequency (VAF) ≥0.05, read

depth ≥10, and non-synonymous annotation. Bar charts show total number of variants identified in tumor margin (M) and tumor center (C), stratified by predicted impact. (**B**) Distribution of variant allele frequencies of filtered variants. Density plots show VAF distributions for tumor margin (M) and tumor center (C). Tumor-only variant calling can include variants with germline-like VAF peaks at approximately 0.5 and 1.0. (**C**) Classification of variants based on annotation and population frequency. Variants were categorized using Ensembl Variant Effect Predictor annotations, dbSNP identifiers, ClinVar annotation, and population frequency databases (GRCh38 build). Bars show counts for tumor margin (M) and tumor center (C) across the indicated categories, including known variants, variants common in population databases, variants not found in reference databases, and ClinVar annotations.

Figure S2

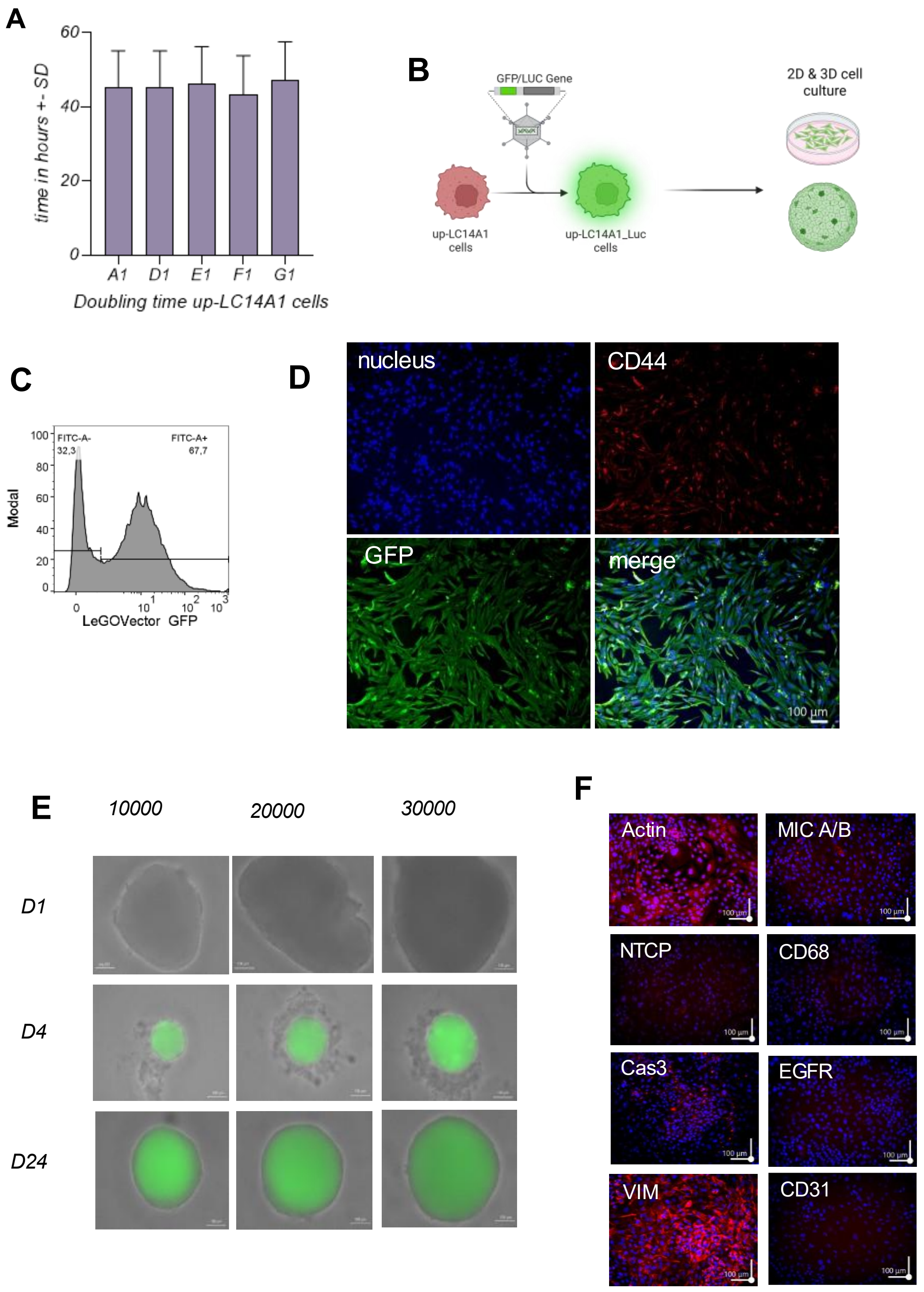

Fig. S2. Phenotypic characteristics, lentiviral modification by LeGoVector, spheroid forming capacities and Protein expression level of up-LC14A1 cells. (A) Impendence measurement based doubling time analysis of up-LC14A1 cell replicates (A1, D1, E1, F1,

G1). Bars represent mean ± SD. (**B**) Illustration of the LeGoVektor-based modification using up-LC14A1 cells to generate up-LC14A1luc cells (MOI10 and 100). Up-LC14A1luc cells were analyzed using flow cytometry (**C**), immunofluorescence staining (**D**), and spheroid forming (**E**). (**C**) Flow cytometric analysis 7days after of LegoVector (GFP/luc2) transduction. Histogram shows over 60% GFP-positive cells. (**D**) Representative fluorescence microscopy of reporter-labeled up-LC14A1 cells in 2D culture showing GFP expression together with CD44 staining and nuclear counterstaining. Scale bars, 100 µm. (**E**) Representative images of spheroid formation by up-LC14A1_luc cells. Spheroids were generated at the indicated seeding densities and imaged at the indicated time points. GFP signal marks reporter-expressing cells. Scale bars, 100µm. (**F**) Representative merged captures of immunofluorescence staining of up-LC14A1 cells grown in 2D culture. Cells were stained for the indicated markers, including structural, epithelial, immune-related, and signaling proteins. Nuclei were stained with Hoechst. Scale bars, 100 µm.

Figure S3

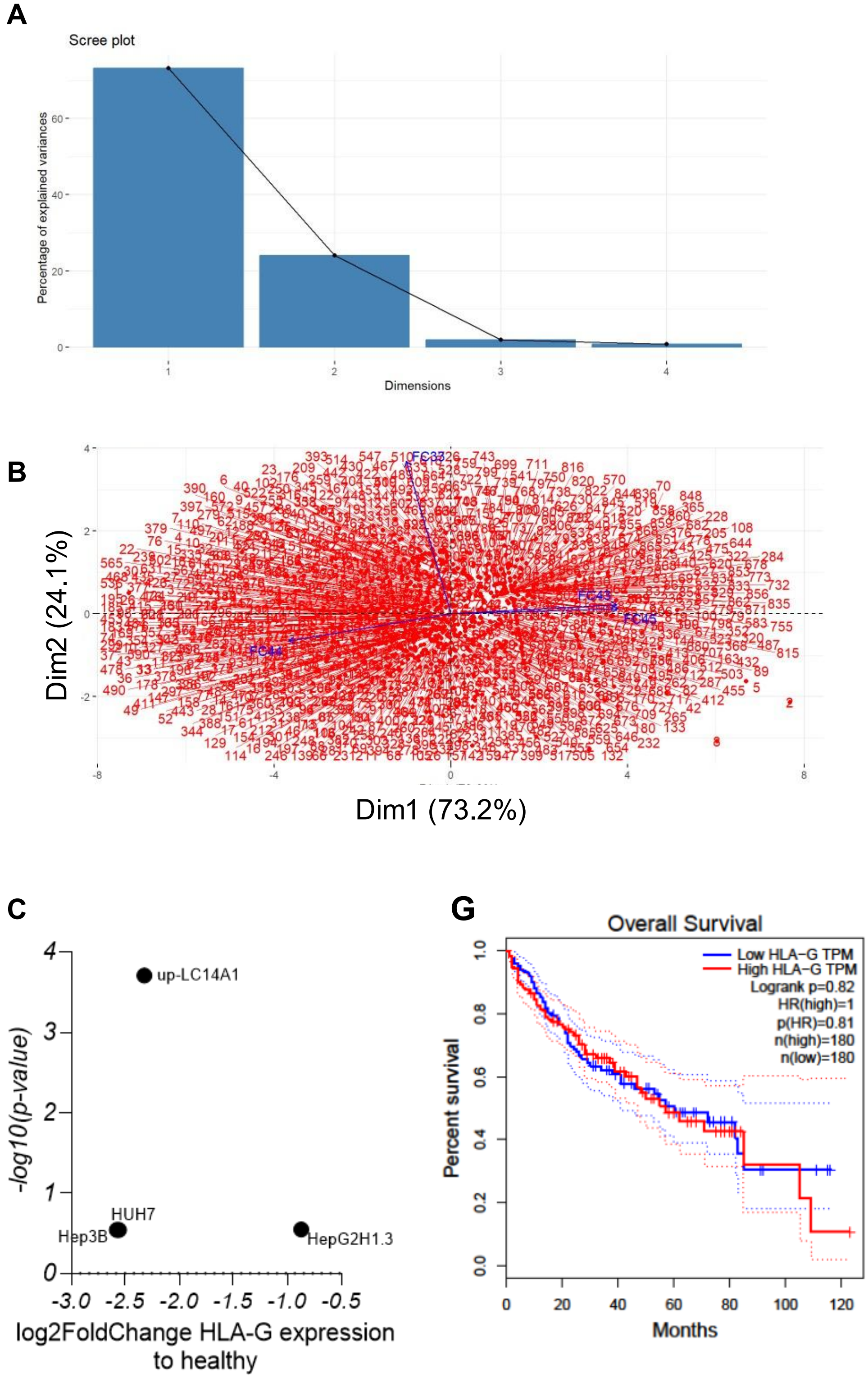

A
Scree plot
Percentage of explained variances
Dimensions
B
Dim2 (24.1%)
Dim1 (73.2%)
C
up-LC14A1
HUH7
Hep3B
HepG2H1.3
-log10(p-value)
log2FoldChange HLA-G expression to healthy
G
Overall Survival
Low HLA-G TPM
High HLA-G TPM
Logrank p=0.82
HR(high)=1
p(HR)=0.81
n(high)=180
n(low)=180
Percent survival
Months

Fig. S3 Principal Component Analysis (PCA) of up-LC14A1 cells and HLA-G expression. (**A**) Scree plot showing the percentage of variance explained by each principal component. The first component (Dim1) explains the majority of the variance (73.2%), followed by Dim2 (24.1%). (**B**) PCA plot of the first two principal components (Dim1 and Dim2) for HCC cell lines. Each point represents an individual sample, with the dimensions Dim1 (73.2%) and Dim2 (24.1%) labeled on the axes. The plot highlights the distribution and clustering of the samples based on their gene expression profiles. FC33 = up-LC14A1 vs PHH; FC43 = Hep3B vs PHH; FC44 = HepG2H1.3 vs PHH; FC45 = HUH7 vs PHH). (**C**) Vulcano plot showing data extracted from DEG data sets of the indicated cell lines for HLA-G. (**D**) Overall survival data, corresponding to HLA-G expression level, extracted from GEPIA (https://gepia3.bioinfoliu.com/genecard/#HLA-G).

Figure S4

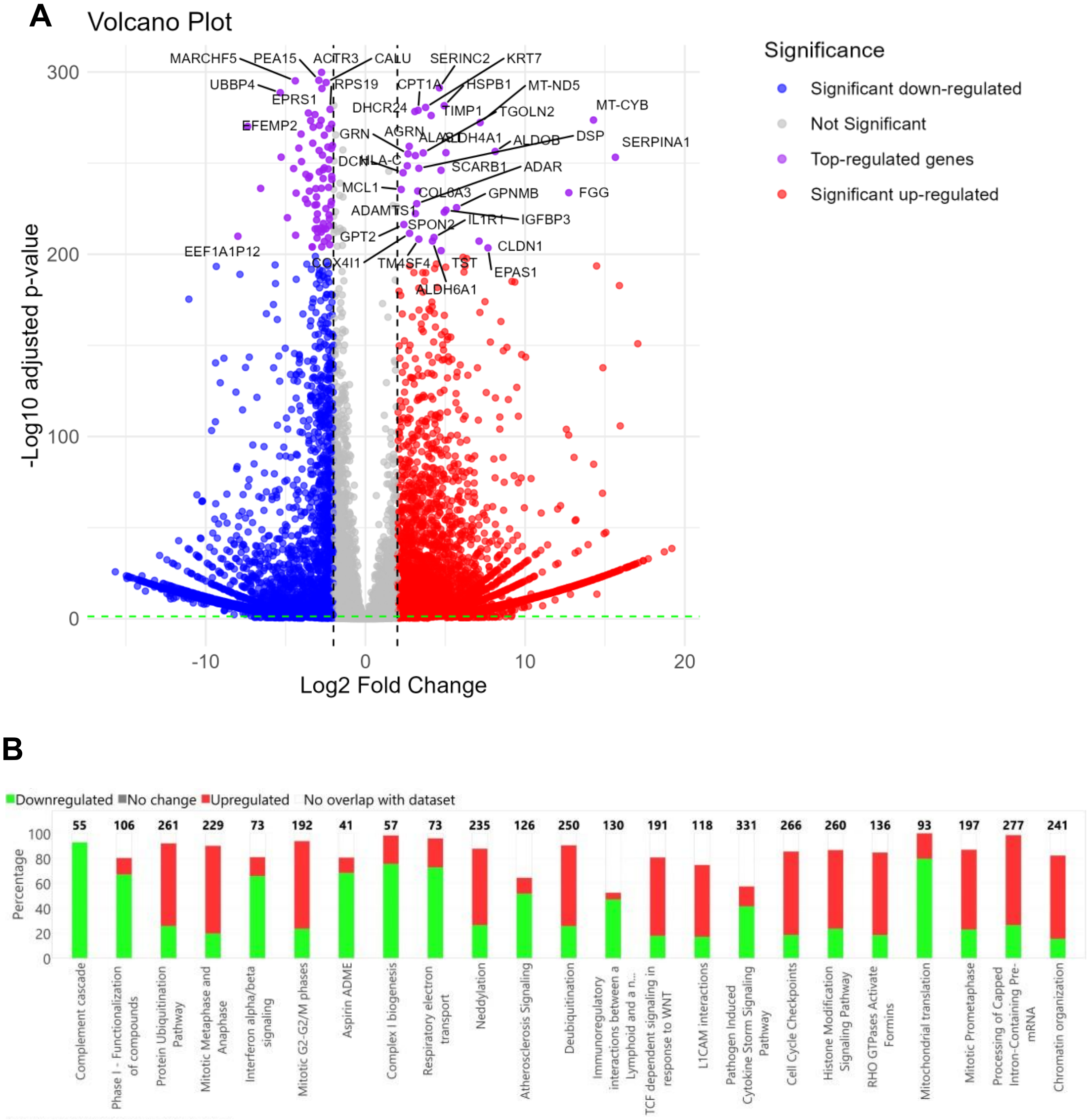

Fig. S4. Differential gene expression and pathway analysis of up-LC14A1 cells compared with primary human hepatocytes. (**A**) Volcano plot of the differential gene expression (DEG) analysis comparing up-LC14A1 cells with primary human hepatocytes (PHH). Genes are plotted according to log2 fold change and −log10 adjusted p value. Significantly upregulated genes are shown in red, significantly downregulated genes in blue, and non-significant genes in grey. Selected highly regulated genes are annotated.

(**B**) Expression profile of genes involved in selected canonical pathways. Bar plots generated using Ingenuity Pathway Analysis (IPA) show the proportion of genes within each pathway that were downregulated (green) or upregulated (red) in up-LC14A1 cells

compared with PHH. The number of genes from the dataset contributing to each pathway is indicated above the bars. PHH, primary human hepatocytes.

Figure S5

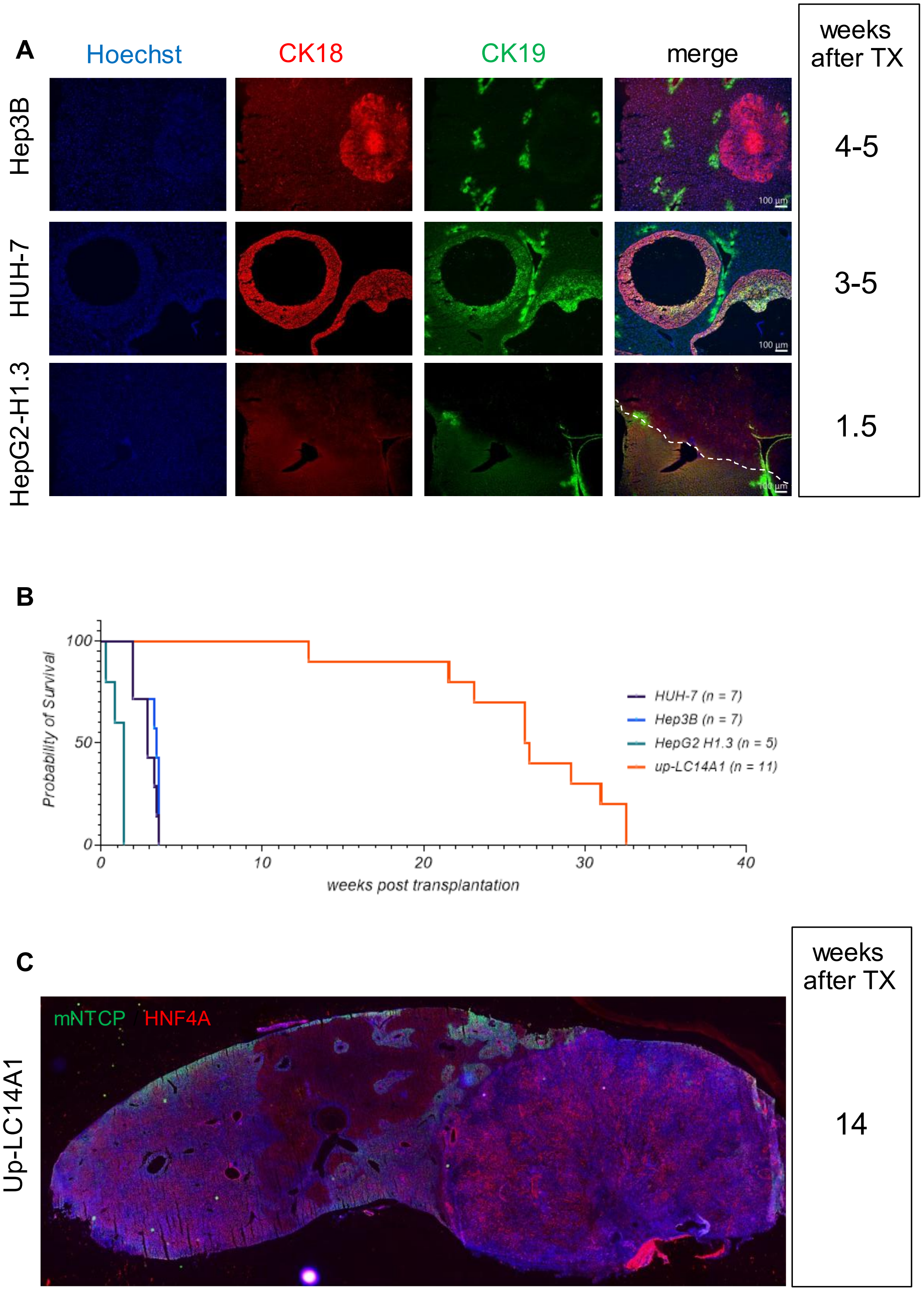

Fig. S5 Comparison of immortalized HCC cell lines and up-LC14A1 cells *in-vivo*

(**A**) Immunofluorescence staining of Hep3B, HUH-7, and HepG2.1.3 cells showing expression of cytokeratin (CK18 in red, CK19 in green). Nuclei were stained with Hoechst (blue). Representative images of epithelial marker expression across different cell lines are shown. Scale bar, 100 µm. (**B**) Kaplan-Meier Survival analysis of xenografted mice using the indicated HCC cell lines. Transplantation was performed with the standardized protocol, as described previously (2). (**C**) Representative immunofluorescence staining of up-LC14A1 xenograft sections showing co-expression of mNTCP (green) and HNF4A (red), indicating hepatocyte-like differentiation in the xenograft. Nuclei were stained with Hoechst (blue). On the right side of the figure, the weeks after transplantation and before scarification of the mice which were used for cryosection-based immunofluorescence staining, are mentioned.

Figure S6

A

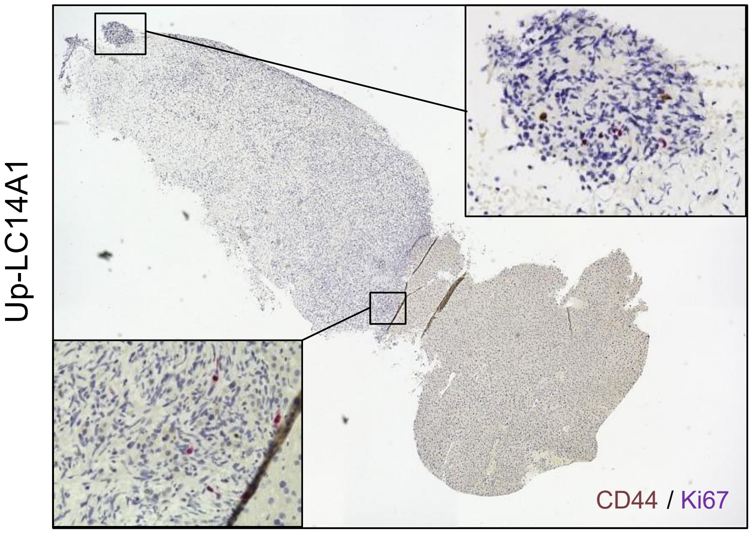

Fig. S6 Characterization up-LC14A1 cells on ki67 and CD44 expression after 12 weeks of progression in-vivo. Immunohistochemistry of up-LC14A1 xenograft sections showing co-expression of CD44 (left inset; brown) and Ki67 (right inset; red). The data demonstrate moderate proliferative activity and stem cell-like features in up-LC14A1 cells in-vivo. Scale bar, 500 µm (main image), 100 µm (insets).

Figure S7

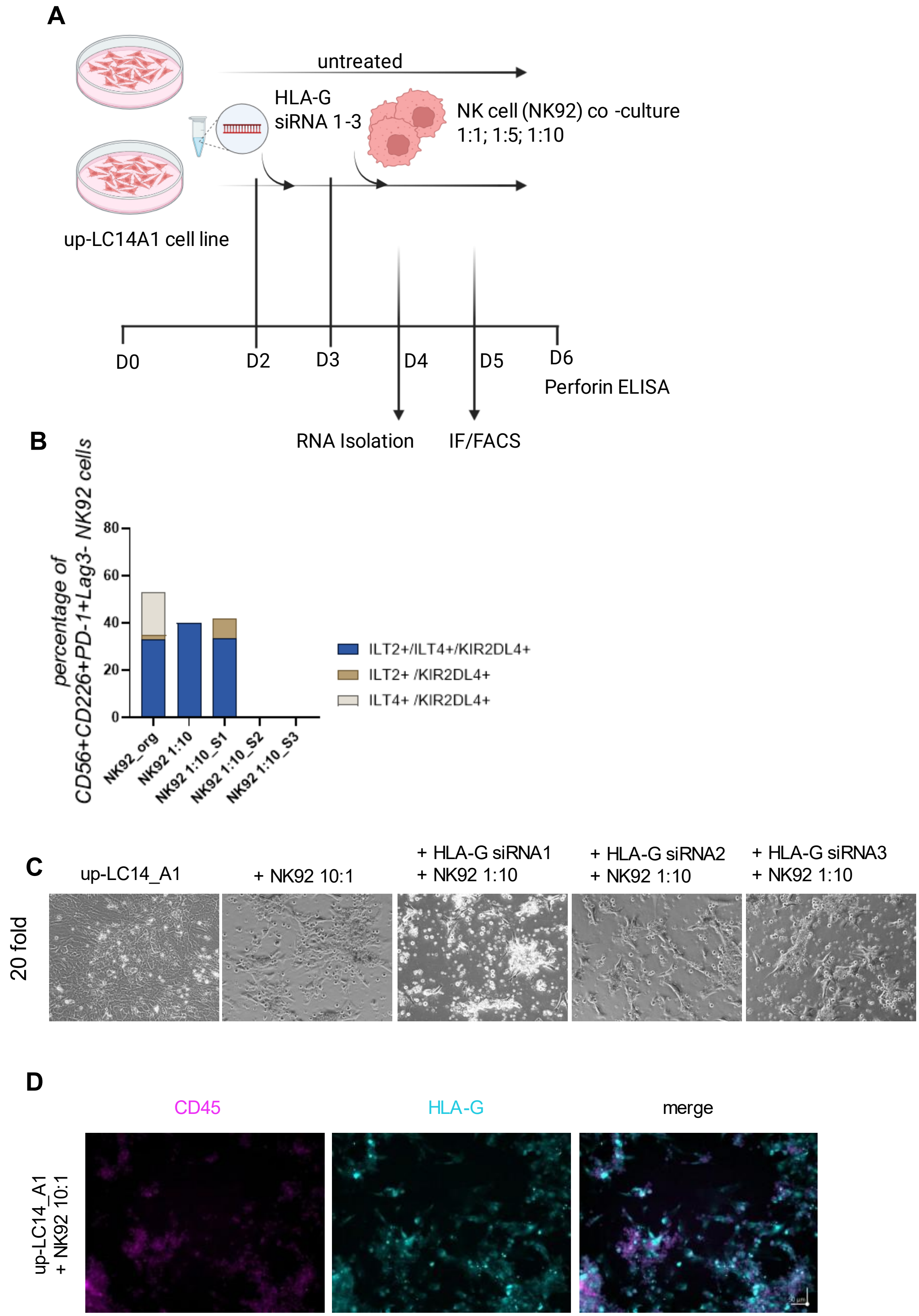
A
untreated
HLA-G
siRNA 1-3
NK cell (NK92) co -culture
1:1; 1:5; 1:10
up-LC14A1 cell line
D0
D2
D3
D4
D5
D6
Perforin ELISA
RNA Isolation
IF/FACS
B
percentage of
CD56+CD226+PD-1+Lag3- NK92 cells
0
20
40
60
80
NK92_org
NK92 1:10
NK92 1:10_S1
NK92 1:10_S2
NK92 1:10_S3
ILT2+/ILT4+/KIR2DL4+
ILT2+ /KIR2DL4+
ILT4+ /KIR2DL4+
C
up-LC14_A1
+ NK92 10:1
+ HLA-G siRNA1
+ NK92 1:10
+ HLA-G siRNA2
+ NK92 1:10
+ HLA-G siRNA3
+ NK92 1:10
20 fold
D
CD45
HLA-G
merge
up-LC14_A1
+ NK92 10:1

Fig. S7 HLA-G siRNA based knockdown experiments, KIR2DL4 expression and microscopy following treatment. (**A**) Visualisation of treatment settings, strategy of sample generation as well as acquisition for gene and protein expression after co-culture conditions. (**B**) Percentage of NK92 subpopulations expressing KIR2DL4 for the indicated treatment conditions. (**C**) 20fold phase contrast bright field cell culture captures, 48 hours after NK92 co-culture corresponding to immunofluorescence staining shown in Fig. 6 and 7. (**D**) Single channel captures of HLA-G and CD45, as well as the merged capture shown in Fig. 7.

3) Supplementary tables

Table S1:

a) N13HCC cohort clinical data

patient number	sex	age	clinical classification	entity	intratumoral HBV RNA/DNA	sHLA-G detection material
2	f	62	DD HCC	Steatohepatitis	NEG	no
3	m	64	HCC G0	ETOH	NEG	no
4	m	70	HCC	viral-induced (HCV-HIV)	NEG	yes
6	f	83	HCC	AIH/PBC	POS	yes
7	m	55	HCC G1	ETOH	NEG	yes
8	m	58	HCC G1	viral-induced (HCV-HBV)	NEG	yes
9	f	69	HCC G1	MASH	POS	yes
10	f	55	HCC	ETOH	NEG	no
11	m	58	HCC G1	viral-induced (HBV)	POS	yes

12	m	69	HCC G1	viral-induced (HBV/HEV)	POS	no
13	m	81	HCC G2	MASH	POS	yes
14	m	31	HCC G3	viral-induced (HBV)	POS	yes
15	m	82	HCC G1	Steatohepatitis	POS	yes

b) early-stage biopsy clinical data

patient number	sex	age in years / month	clinical classification	sHLA-G detection material
124-biopsy	m	65	viral hepatitis	yes
171 – biopsy	f	73	Autoimmune Hepatitis	yes
196 - biopsy	m	51	Liver cirrhosis	yes
193 - biopsy	f	50	Alcoholic fibrosis and sclerosis	yes

Table S2: Taqman assay for real-time PCR

gene name	Assay-ID	dye
RPL0	Hs00420895_gH	FAM-MGB
RPL30	Hs00265497_m1	VIC-MGB
GAPDH	Hs99999905_M1	FAM-MGB
HMGCoAR	Hs00168352_m1	FAM-MGB
HBV S	Pa03453405_s1	FAM-MGB
CD44	Hs01075864_m1	FAM-MGB
HNF4A	Hs00230853_m1	VIC-MGB
STAT3	Hs00374280_m1	VIC-MGB
HLA-G	Hs03045108_m1	VIC-MGB
CLRN3	Hs00153677_m1	FAM-MGB
AADAC	Hs00153677_m1	FAM-MGB

ALB	Hs00609411_m1	VIC-MGB
MIK67	Hs04260396_g1	FAM-MGB
TP53	HS01034249_m1	FAM-MGB
IFNg	Hs00989291_m1	FAM-MGB
NLRP3	Hs00918082_m1	VIC-MGB
CD8	Hs00233520_m1	FAM-MGB
CD45	Hs04189704_m1	FAM-MGB
CD24	Hs02379687_s1	FAM-MGB
Glut-1	Hs00892681_m1	FAM-MGB

Table S3: Antibodies for processed Flow cytometry of NK cells

antigen	fluorochrome	company
CD3	BUV395	BD Horizon
CD14	FITC	BD Pharmingen
CD45	BV480	BD Horizon
CD56	PE	BD Pharmingen
CD16	BV650	BD Horizon
CD19	BV786	BD Horizon
CD62L, L-Selectin	BUV496	BD OptiBuild
CD178, Fas-Ligand	BUV615	BD Optibuild
CD226, DNAM1	BUV737	BD Optibuild
CD335, NKp46	BV510	BD Horizon
CD162, PGSL-1	BV650	BD Optibuild
CD336, NKp44	RB780	BD Horizon
CD337, NKp30	R718	BD Horizon
CD253, TRAIL	BV421	BD Horizon
CD314, NKGD2	PerCP-Cy5.5	BD Pharmingen
CD279, PD-1	BV711	BD OptiBuild
CD11a, LFA-1 a-chain	BUV496	BD Optibuild
CD49d, VLA-4 a-chain, Integrin a	BUV615	BD Horizon
CD107a	BV510	BD Horizon
CD184, CXCR4	BV650	BD OptiBuild
TIGIT	RB780	BD Horizon
CD183, CXCR3	R718	BD Horizon
CD15s	BV421	BD Horizon
CD366, TIM-3	PerCP-Cy5.5	BD Pharmingen
CD197, CCR7	BV711	BD Horizon
ILT2, CD85j	BUV615	BD Optibuild
ILT4, CD85d	BV421	Miltenyi Bioscience
KIR2DL4, CD158d	PE-Cy5	Thermo Fischer
CD223, Lag-3	BV605	BD Horizon

Table S4: Antibodies for processed Flow cytometry for HCC cells- ex-vivo and in-vitro

antigen	fluorochrome	company
CD105	PE-Vio® 770	Miltenyi Biotec B.V. & Co. KG, D
CD13	APC	Miltenyi Biotec B.V. & Co. KG, D
CD133/1	PE-Vio 615	Miltenyi Biotec B.V. & Co. KG, D
CD24	PE-Vio 770	Miltenyi Biotec B.V. & Co. KG, D
CD279 (PD1)	PE	Miltenyi Biotec B.V. & Co. KG, D
CD3	PE-Vio® 770	Miltenyi Biotec B.V. & Co. KG, D
CD326	PE-Vio615	Miltenyi Biotec B.V. & Co. KG, D
CD326	VioBlue	Miltenyi Biotec B.V. & Co. KG, D
CD34	PE-Vio® 770	Miltenyi Biotec B.V. & Co. KG, D
CD366	APC	Miltenyi Biotec B.V. & Co. KG, D
CD44	APC	Miltenyi Biotec B.V. & Co. KG, D
CD45	APC-Vio® 770	Miltenyi Biotec B.V. & Co. KG, D
CD73	PE	Miltenyi Biotec B.V. & Co. KG, D
CD73	PE-Vio® 615	Miltenyi Biotec B.V. & Co. KG, D
CD90	VioBlue	Miltenyi Biotec B.V. & Co. KG, D
CD90	PE	Miltenyi Biotec B.V. & Co. KG, D
HLA-G	PE	Miltenyi Biotec B.V. & Co. KG, D

Table S5: antibodies for processed IF and IHC staining

Antigen	Company	Catalog number
Vimentin	Santa Cruz	SC-37317
CD31	Invitrogene	PA5-16301
CK18	ExBIO	11-107-C100
PD-L1	GeneTex	GTx57193
CD68	Dako	M0876
Calnexin	ThermoFischer	MA5-32332
CD44	ThermoFischer	PA5-114983
CK19	Avivasysbio	OABB00294/100ug
NTCP	Sigma	HPA042727
HNF4alpha	ThermoFischer	PA5-18363
Ki67	Origene	TA801156
Rb pAb Actin	Abcam	ab5694
EGFR	santa Cruz	sc-120
CK7	Dako Aglient	M7018
HBsAG	Dako Aglient	B0560
AFP	ThermoFischer	TA501788
Caspase3	Invitrogene	710431
AADAC	proteintech	26634-I-AP
MICA/B	eBioscience	5013153
HLA-G	ThermoFischer	PA5-98143

CD45-FITC	ThermoFischer	MHCD4520
HLA-ABC	Origene	SM1222LE

Table S6: PCR Primer pairs for cf-DNA measurement

Primer		Sequence (5'->3')
ALU_F_1	Forward primer	CCTGAGGTCAGGAGTTCGAG
ALU_R_1	Reverse primer	CCCGAGTAGCTGGGATTACA
ALU_F_2	Forward primer	GTGGCTCACGCCTGTAATC
ALU_R_2	Reverse primer	CAGGCTGGAGTGCAGTGG
MTCO_F_1	Forward primer	TAAACTTCAACCAACACCGT
MTCO_R_1	Reverse primer	TAGACTTCTGGGTGGCCAAAGA
MTCO_F_2	Forward primer	GACCTGATGCACTGAGGTTT
MTCO_R_2	Reverse primer	GTTTACGAGGCTTCTTCTG

Supplementary references

1. Kah J, Staffeldt L, Volz T, Schulze K, Heumann A, Rövenstrunk G, et al. Classification of the LC4 Primarily-like Cell Line—Recapitulating a CDK4 Overexpressing Immune Evasive HIV-HCV-Induced HCC. Viruses [Internet]. 2025; 17(5).
2. Staffeldt L, Mattert G, Riecken K, Rövenstrunk G, Volkmar A, Heumann A, et al. Generating Patient-Derived HCC Cell Lines Suitable for Predictive In Vitro and In Vivo Drug Screening by Orthotopic Transplantation. Cells. 2023;13(1).
3. Herzog N, Hansen M, Miethbauer S, Schmidtke KU, Anderer U, Lupp A, et al. Primary-like human hepatocytes genetically engineered to obtain proliferation competence display hepatic differentiation characteristics in monolayer and organotypical spheroid cultures. Cell Biol Int. 2016;40(3):341–53.
4. Burkard A, Dähn C, Heinz S, Zutavern A, Sonntag-Buck V, Maltman D, et al. Generation of proliferating human hepatocytes using Upcyte® technology: characterisation and applications in induction and cytotoxicity assays. Xenobiotica. 2012;42(10):939–56.
5. Wuestenberg A, Kah J, Singethan K, Sirma H, Keller AD, Rosal SR, et al. Matrix conditions and KLF2-dependent induction of heme oxygenase-1 modulate inhibition of HCV replication by fluvastatin. PLoS One. 2014;9(5):e96533.

6. Allweiss L, Volz T, Lutgehetmann M, Giersch K, Bornscheuer T, Lohse AW, et al. Immune cell responses are not required to induce substantial hepatitis B virus antigen decline during pegylated interferon-alpha administration. J Hepatol. 2014;60(3):500–7.
7. Linke C, von Hänisch T, Schröder J, Dammermann W, Deckert PM, Reinwald M, et al. Heterogeneous Formation of DNA Double-Strand Breaks and Cell-Free DNA in Leukemia T-Cell Line and Human Peripheral Blood Mononuclear Cells in Response to Topoisomerase II Inhibitors. Cancers (Basel). 2024;16(22).
