# Supplementary material for: Targeting a tolerogenic HLA-G genotype to tackle immune evasion and adaptive resistance in HBV-driven HCC
Source: JHEP Rep. 2026 May 8;8(8):101891. doi: 10.1016/j.jhepr.2026.101891 (PMC13352049; doi:10.1016/j.jhepr.2026.101891)
Supplement: Multimedia compoennt 2 [file mmc2.docx]

**JHEP Reports**

**CTAT methods**

Tables for a “Complete, Transparent, Accurate and Timely account” (CTAT) are now mandatory for all revised submissions. The aim is to enhance the reproducibility of methods.

- Only include the parts relevant to your study
- Refer to the CTAT in the main text as ‘Supplementary CTAT Table’
- Do not add subheadings
- Add as many rows as needed to include all information
- Only include one item per row

**If the CTAT form is not relevant to your study, please outline the reasons why:**

| Not applicable – CTAT table completed below. |
| --- |

- 1. **Antibodies**

| **Name** | **Citation** | **Supplier** | **Cat no.** | **Clone no.** |
| --- | --- | --- | --- | --- |
| CD3 (BUV395) | Table S3 | BD Horizon | 563914 | UCHT1 |
| CD14 (FITC) | Table S3 | BD Pharmingen | 555397 | MϕP9 |
| CD45 (BV480) | Table S3 | BD Horizon | 566432 | HI30 |
| CD56 (PE) | Table S3 | BD Pharmingen | 340395 | B159 |
| CD16 (BV650) | Table S3 | BD Horizon | 746188 | 3G8 |
| CD19 (BV786) | Table S3 | BD Horizon | 563333 | SJ25C1 |
| CD62L (BUV496) | Table S3 | BD OptiBuild | 749933 | SK11 |
| Fas-L (CD178, BUV615) | Table S3 | BD OptiBuild | 744729 | NOK-1 |
| DNAM1 (CD226, BUV737) | Table S3 | BD OptiBuild | 749774 | 11A8 |
| NKp46 (CD335, BV510) | Table S3 | BD Horizon | 745211 | 9E2 |
| NKp44 (CD336, RB780) | Table S3 | BD Horizon | 747681 | P44-8 |
| NKp30 (CD337, R718) | Table S3 | BD Horizon | 747548 | P30-15 |
| TRAIL (CD253, BV421) | Table S3 | BD Horizon | 747034 | RI-K2 |
| NKG2D (CD314, PerCP-Cy5.5) | Table S3 | BD Pharmingen | 557698 | 1D11 |
| PD-1 (CD279, BV711) | Table S3 | BD OptiBuild | 749268 | EH12.2H7 |
| CXCR4 (CD184, BV650) | Table S3 | BD OptiBuild | 747430 | 12G5 |
| TIGIT (RB780) | Table S3 | BD Horizon | 749573 | VSTM3 |
| TIM-3 (CD366, PerCP-Cy5.5) | Table S3 | BD Pharmingen | 565692 | 7D3 |
| CCR7 (CD197, BV711) | Table S3 | BD OptiBuild | 747810 | G043H7 |
| ILT2 (CD85j, BUV615) | Table S3 | BD OptiBuild | 751022 | HPF1 |
| ILT4 (CD85d, BV421) | Table S3 | Miltenyi Biotec | 130-110-692 | REA203 |
| KIR2DL4 (CD158d, PE-Cy5) | Table S3 | Thermo Fisher | 15-1589-42 | 33A1-4 |
| LAG-3 (CD223, BV605) | Table S3 | BD Horizon | 745286 | 17B4 |
| HLA-G (PE) | Table S4 | Miltenyi Biotec | 130-111-877 | REA176 |
| Vimentin | Table S5 | Santa Cruz | SC-37317 | V9 |
| CD31 | Table S5 | Invitrogene | PA5-16301 | JC70A |
| CK18 | Table S5 | ExBIO | 11-107-C100 | DC10 |
| PD-L1 | Table S5 | GeneTex | GTx57193 | 28-8 |
| CD68 | Table S5 | Dako | M0876 | KP1 |
| Calnexin | Table S5 | ThermoFischer | MA5-32332 | C5C9 |
| CD44 | Table S5 | ThermoFischer | PA5-114983 | IM7 |
| CK19 | Table S5 | Avivasysbio | OABB00294/100ug | A53-B/A2 |
| NTCP | Table S5 | Sigma | HPA042727 | Polyclonal |
| HNF4alpha | Table S5 | ThermoFischer | PA5-18363 | Polyclonal |
| Ki67 | Table S5 | Origene | TA801156 | UMAB107 |
| Rb pAb Actin | Table S5 | Abcam | ab5694 | AC-15 |
| EGFR | Table S5 | santa Cruz | sc-120 | 528 |
| CK7 | Table S5 | Dako Aglient | M7018 | OV-TL 12/30 |
| HBsAG | Table S5 | Dako Aglient | B0560 | Polyclonal |
| AFP | Table S5 | ThermoFischer | TA501788 | C3 |
| Caspase3 | Table S5 | Invitrogene | 710431 | Polyclonal |
| AADAC | Table S5 | proteintech | 26634-I-AP | Polyclonal |
| MICA/B | Table S5 | eBioscience | 5013153 | 6D4 |
| HLA-G | Table S5 | ThermoFischer | PA5-98143 | 87G |
| CD45-FITC | Table S5 | ThermoFischer | MHCD4520 | HI30 |
| HLA-ABC | Table S5 | Origene | SM1222LE | W6/32 |

- 1. **Cell lines**

| **Name** | **Citation** | **Supplier** | **Cat no.** | **Passage no.** | **Authentication test method** |
| --- | --- | --- | --- | --- | --- |
|  |  |  |  |  |  |
| up-LC14A1 | This study; Suppl. Methods | Patient-derived HBV-HCC (Upcyte® modified) |  |  | Not reported |
| up-LC14A1_luc (LeGO-iG2-Puro+-Luc2) | Suppl. Methods | Lentiviral transduction (LeGO-iG2-Puro+-Luc2) |  |  | Not reported |
| HUH-7 | Suppl. Methods [5] | Not specified |  |  | Not reported |
| Hep3B | Suppl. Methods [5] | Not specified |  |  | Not reported |
| HepG2-H1.3 | Suppl. Methods [5] | Not specified |  |  | Not reported |
| NK92 | Suppl. Methods | Not specified (internal stock) |  |  | Not reported |

- 1. **Organisms**

| **Name** | **Citation** | **Supplier** | **Strain** | **Sex** | **Age** | **Overall n number** |
| --- | --- | --- | --- | --- | --- | --- |
|  |  |  |  |  |  |  |
| Immunodeficient mice (orthotopic xenograft model) | Suppl. Methods [2] | https://www.criver.com/jax-nsg-mouse-variant-portfolio | NSG | Not specified | Not specified | AAT endpoint: HUH-7 n=3; Hep3B n=4; HepG2-H1.3 n=4; up-LC14A1 n=14 (see Suppl. Methods) |

- 1. **Sequence based reagents**

| **Name** | **Sequence** | **Supplier** |
| --- | --- | --- |
|  |  |  |
| HLA-G siRNA set (Gene ID 3135; Set 15062) | cat#: HY-RS06217 | MCE |
| TaqMan assay RPL0 | Hs00420895_gH | Life Technologies |
| TaqMan assay RPL30 | Hs00265497_m1 | Life Technologies |
| TaqMan assay GAPDH | Hs99999905_M1 | Life Technologies |
| TaqMan assay HBV S | Pa03453405_s1 | Life Technologies |
| TaqMan assay HLA-G | Hs03045108_m1 | Life Technologies |
| TaqMan assay NLRP3 | Hs00918082_m1 | Life Technologies |
| TaqMan assay CD8 | Hs00233520_m1 | Life Technologies |
| TaqMan assay CD45 | Hs04189704_m1 | Life Technologies |
| TaqMan assay CD24 | Hs02379687_s1 | Life Technologies |
| TaqMan assay GLUT1 | Hs00892681_m1 | Life Technologies |
| TaqMan assay HMGCoAR | Hs00168352_m1 | Life Technologies |
| TaqMan assay CD44 | Hs01075864_m1 | Life Technologies |
| TaqMan assay HNF4A | Hs00230853_m1 | Life Technologies |
| TaqMan assay STAT3 | Hs00374280_m1 | Life Technologies |
| TaqMan assay CLRN3 | Hs00153677_m1 | Life Technologies |
| TaqMan assay AADAC | Hs00153677_m1 | Life Technologies |
| TaqMan assay ALB | Hs00609411_m1 | Life Technologies |
| TaqMan assay MKI67 | Hs04260396_g1 | Life Technologies |
| TaqMan assay TP53 | HS01034249_m1 | Life Technologies |
| TaqMan assay IFNG | Hs00989291_m1 | Life Technologies |
| ALU-115 forward primer | CCTGAGGTCAGGAGTTCGAG | Custom (supplier not specified) |
| ALU-115 reverse primer | CCCGAGTAGCTGGGATTACA | Custom (supplier not specified) |
| ALU-247 forward primer | GTGGCTCACGCCTGTAATC | Custom (supplier not specified) |
| ALU-247 reverse primer | CAGGCTGGAGTGCAGTGG | Custom (supplier not specified) |
| MTCO_F_1 forward primer | TAAACTTCAACCAACACCGT | Custom (supplier not specified) |
| MTCO_R_1 reverse primer | TAGACTTCTGGGTGGCCAAAGA | Custom (supplier not specified) |
| MTCO_F_2 forward primer | GACCTGATGCACTGAGGTTT | Custom (supplier not specified) |
| MTCO_R_2 reverse primer | GTTTACGAGGCTTCTTCTG | Custom (supplier not specified) |

- 1. **Biological samples**

| **Description** | **Source** | **Identifier** |
| --- | --- | --- |
|  |  |  |
| HBV-positive HCC tumor tissue (tumor center) | University Medical Center Hamburg-Eppendorf | Ethics PV-3578 |
| Human blood/serum samples (HBV-HCC cohort) | University Medical Center Hamburg-Eppendorf | Ethics PV-3578 |
| Primary human hepatocytes (PHH) | As described previously [1] | Not specified |
| Mouse xenograft liver tissue (tumorigenic/non-tumorigenic areas) | Animal facility Hamburg | Animal approval N056/2020 |

- 1. **Deposited data**

| **Name of repository** | **Identifier** | **Link** |
| --- | --- | --- |
|  |  |  |
| Not deposited | Not applicable | Not applicable (available upon reasonable request) |

- 1. **Software**

| **Software name** | **Manufacturer** | **Version** |
| --- | --- | --- |
| GraphPad Prism | GraphPad Software | v10 |
| xCELLigence RTCA Software | ACEA Biosciences | v2.0 |
| Design & Analysis Software | Thermo Fisher Scientific | v2.8.0 |
| Varvis® | IVD-CE Varvis® | v2.7.3 |
| R | R Foundation | v4.x |
| RStudio | Posit | Not specified |
| DESeq2 | Bioconductor | Not specified |
| STAR | Not specified | Not specified |
| Trimmomatic | Not specified | Not specified |
| featureCounts | Subread package | Not specified |
| Rtsne | CRAN | Not specified |
| ggplot2 | CRAN | Not specified |

- 1. **Other (*e.g*. drugs, proteins, vectors etc.)**

| Item | Supplier | Identifier (Cat no.) |
| --- | --- | --- |
| ViraPower™ Lentiviral Expression System | Thermo Fisher Scientific | Not specified |
| Vivaspin® 20 concentrator | Sartorius | Not specified |
| Detachin™ Cell Detachment Solution | AMSBIO | Not specified |
| BIOFLOAT™ cell culture plates | Not specified | Not specified |
| xCELLigence RTCA SP device | ACEA Biosciences | Not specified |
| E-Plate 16 | ACEA Biosciences | Not specified |
| RNeasy Micro™ kit | Qiagen | Not specified |
| RNeasy Mini™ kit | Qiagen | Not specified |
| MMLV Reverse Transcriptase™ 1st-Strand cDNA kit | Lucigen | Not specified |
| QuantStudio 7™ Real-Time PCR System | Life Technologies | Not specified |
| Twist Precision Exome DX kit | Twist Bioscience | Not specified |
| Illumina NextSeq 1000/2000 sequencing platform | Illumina | Not specified |
| TruSeq Stranded mRNA Kit | Illumina | Not specified |
| Human MHC Class I G (HLA-G) ELISA Kit | Thermo Fisher Scientific | Not specified |
| Human alpha-1 antitrypsin (AAT) ELISA Kit | Abcam | Not specified |
| ELISA Flex: Human Perforin (HRP) | Mabtech | Not specified |
| QIAamp MinElute™ Virus Spin Kit | Qiagen | Not specified |
| MagMAX™ cfDNA Isolation Kit | Thermo Fisher Scientific | Not specified |
| PowerUp™ SYBR™ Green Master Mix | Thermo Fisher Scientific | Not specified |

- 1. **Please provide the details of the corresponding methods author for the manuscript:**

| Corresponding methods author: Dr. rer. nat. Janine Kah (University Hospital Brandenburg, Department of Gastroenterology; Centre for Translational Medicine). |
| --- |

**2.0 Please confirm for randomised controlled trials all versions of the clinical protocol are included in the submission. These will be published online as supplementary information.**

| Not applicable – this study does not include a randomized controlled clinical trial. |
| --- |
