# Supplementary material for: Targeting a tolerogenic HLA-G genotype to tackle immune evasion and adaptive resistance in HBV-driven HCC
Source: JHEP Rep. 2026 May 8;8(8):101891. doi: 10.1016/j.jhepr.2026.101891 (PMC13352049; doi:10.1016/j.jhepr.2026.101891)
Supplement: Multimedia component 4 [file mmc4.pdf]

Research article

JHEP|Reports

Targeting a tolerogenic HLA-G genotype to tackle immune evasion and adaptive resistance in HBV-driven HCC

Authors

Janine Kah, Lisa Staffeldt, Svenja Stefanski, …, Pablo Villavicencio-Lorini, Maura Dandri, Stefan Lüth

Correspondence

janine.kah@mhb-fontane.de (J. Kah).

Graphical abstract

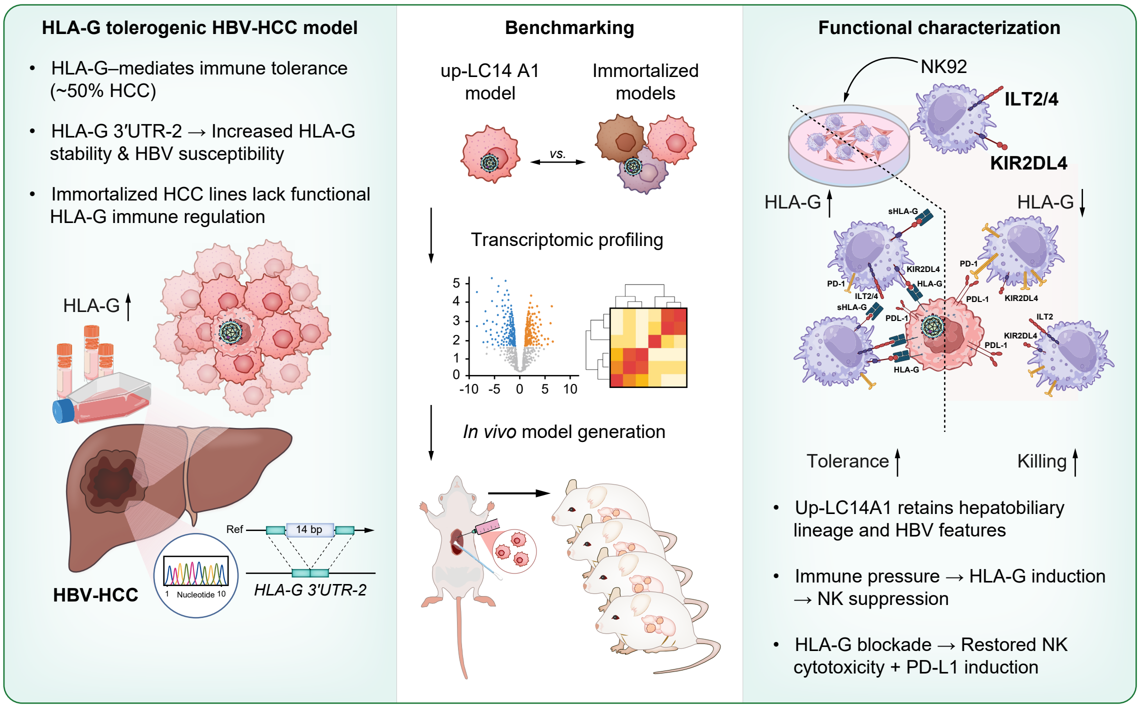

Highlights:

- Patient-derived HCC represents a clinically and molecularly typical HBV-associated HCC.
- WES identifies an HLA-G-mediated immune-tolerogenic profile.
- Patient-derived up-LC14A1 cell line retains parental features.
- Immune pressure induces HLA-G expression, adaptive immune tolerance, and checkpoint switching.
- Orthotopic transplantation induces gradual *in vivo* tumor expansion, enabling evaluation of therapeutic strategies.

Impact and implications:

HBV-associated HCC exhibits a profound immune evasion capacity and an insufficient response to immunotherapy. Nevertheless, preclinical models that mimic viral persistence and tumor resistance are underrepresented. The immune modulatory molecule HLA-G has emerged as a potent target for immunotherapy in HCCs owing to its direct suppression of T and NK cells. Here, we established a novel patient-derived HBV-HCC cell line (up-LC14A1) that mediates immune evasion via HLA-G upregulation.

https://doi.org/10.1016/j.jhepr.2026.101891
© 2026 The Authors. Published by Elsevier B.V. on behalf of European Association for the Study of the Liver (EASL). This is an open access article under the CC BY license (http://creativecommons.org/licenses/by/4.0/).

Targeting a tolerogenic HLA-G genotype to tackle immune evasion and adaptive resistance in HBV-driven HCC

Janine Kah[1,2,3,4,*], Lisa Staffeldt[1,2], Svenja Stefanski[2,4], Natalie Herzog[5], Gregor Mattert[2,4], Tassilo Volz[1,6], Maximilian Voß[2,4], Kornelius Schulze[1], Asmus Heumann[7], Werner Dammermann[2,3,4], Sarah Kammerer[5], Jan-Heiner Küpper[5], Pablo Villavicencio-Lorini[8], Maura Dandri[1,6], Stefan Lüth[2,3,4]

JHEP Reports **2026**. vol. 8 | 1–14

Check for updates

Background & Aims: HBV-associated hepatocellular carcinoma (HCC) is characterized by immune evasion and heterogeneous responses to immunotherapy. However, the mechanisms driving tumor–immune tolerance and their impact on checkpoint inhibitor-based therapies remain poorly understood. To address this gap, we generated a patient-derived HBV-induced HCC cell line that preserves a clinically relevant immune evasion mechanism mediated by human leukocyte antigen G (HLA-G) expression.

Methods: We performed whole-exome sequencing on two spatially separated tumor regions to define genomic signatures with emphasis on the HLA-G locus. Patient-derived tumor cells were modified using upcyte® technology, generating the up-LC14A1 cell line. Molecular stratification included an in-house HCC cohort (n = 13), healthy donors (n = 4), serum samples from early-stage liver disease (n = 4), diagnosed HCC (n = 10), and global datasets (TCGA biopsies, n = 24; HCC, n = 120). Functional characterization included NK92 co-culture assays (1:1, 1:5, 1:10) and orthotopic transplantation in mice (HUH7, n = 3; Hep3B, n = 4; HepG2H1.3, n = 4; up-LC14A1, n = 14).

Results: Whole-exome sequencing revealed a clonally coherent HCC carrying an HLA-G 3′UTR haplotype associated with immune tolerance. Cohort stratification confirmed elevated soluble HLA-G in patient #14. The derived up-LC14A1 line retained hepatobiliary and viral features and showed stable proliferation with low-level HBV DNA production. Transcriptomic profiling positioned up-LC14A1 within the HCC landscape. *In vivo*, orthotopic transplantation generated structured liver tumors and prolonged survival compared with immortalized HCC lines. The up-LC14A1 cell line resisted NK92 cytotoxicity through dynamic *HLA-G* induction, while *HLA-G* silencing restored immune killing and induced compensatory PD-L1 expression.

Conclusions: The up-LC14A1 model represents a patient-derived HBV-HCC system capturing a clonally stable but dynamically regulated HLA-G-mediated immune-tolerant state. This platform enables mechanistic investigation of immune escape and provides a translational model for testing targeted immunotherapies in HBV-associated HCC.

© 2026 The Authors. Published by Elsevier B.V. on behalf of European Association for the Study of the Liver (EASL). This is an open access article under the CC BY license (http://creativecommons.org/licenses/by/4.0/).

Introduction

Hepatocellular carcinoma (HCC) accounts for up to 90% of the primary liver cancers and remains a global health burden, with chronic HBV infection being a predominant etiological factor.[1] Despite progress in the molecular and immunological understanding of HCC, complete response rates to therapy are limited because of late-stage diagnosis, increased relapse rates, and resistance development after treatment.[2] The difficulty lies in the heterogeneous immunosuppressive nature of HCCs, shaped by viral factors, chronic inflammation, and fibrosis, which complicates the identification of broadly effective therapeutic strategies.[3] Remarkably, progress has been made in the treatment of HCC after the advent of sorafenib[4] and the approval of several additional treatment regimens,[5] including tyrosine kinase inhibitors (TKIs) and immune checkpoint inhibitors (ICIs). Nevertheless, the objective response rate following combined TKI-ICI or ICI–ICI therapy remains unsatisfactory.[3] Moreover, patients who develop resistance may exhibit hyperprogressive relapses leading to a worse clinical setting. To improve therapy outcomes for these refractory patients, evaluating novel targets is necessary to manage resistance and prevent its development during therapy.

Among the pleotropic immune evasion mechanisms orchestrated in HCCs, the non-classical major histocompatibility complex (MHC) class I molecule human leukocyte antigen G (HLA-G), found in 50% of primary HCCs,[6] has emerged as a potent immune checkpoint.[7] Unlike programmed death-ligand 1 (PD-L1), HLA-G mediates immune suppression via distinct pathways, inhibiting cytotoxic T cells and NK cells, promoting tolerogenic dendritic cells, and facilitating the expansion of regulatory T cells.[7] Elevated expression of membrane-bound (mHLA-G) and soluble HLA-G (sHLA-G) has been correlated with tumor progression, high recurrence rates,

* Corresponding author. Address: Principal Investigator at the Department of Gastroenterology, Center for Translational Medicine, University Hospital Brandenburg, Brandenburg Medical School Theodor Fontane, 14770 Brandenburg an der Havel, Germany.
E-mail address: janine.kah@mhb-fontane.de (J. Kah).
https://doi.org/10.1016/j.jhepr.2026.101891

and resistance to ICI therapy in HCCs.[6,8,9] In the setting of HBV infection, miR-152, a functional suppressor of HLA-G stability, is reduced, leading to an overexpression of the immune tolerance molecule during active infection.[10] Moreover, genetic polymorphisms in the *HLA-G* gene have reportedly correlate with increased susceptibility to HBV infection.[11] Its overall immunomodulatory properties make HLA-G an interesting target after immunotherapies, especially in virus-induced HCCs, where it can induce resistance mechanisms.

Commonly used immortalized HCC cell lines fail to process the functional immunomodulatory protein,[12] even when they synthesize *HLA-G* mRNA;[6] therefore, genetically modified cell lines remain the gold standard for elucidating HLA-G-mediated immune tolerance. However, suitable models remain lacking for robust investigation of the dynamic immune-tolerogenic pathways and the resulting phenotype adaptations.

Here, we address this unmet need and establish an HBV-HCC patient-derived model that displays the dynamics of HLA-G-mediated immune tolerance. The advantage of this model lies in its low *in vivo* progression rate, which enables orthotopic transplantation, and its retention of original tumor features. This model was generated from an HBV-positive individual exhibiting mixed hepatocellular (70%) and cholangiocellular (30%) carcinoma, carrying HLA-A*03:01P and *30:01P alleles. The patient was initially treated with a nucleotide analog (tenofovir) and subsequently with atezolizumab plus bevacizumab upon relapse; however, the patient experienced rapid disease progression and succumbed 18 months after resection. From the tumor center material, we extracted proliferative competent primary liver cancer cells (LC14) and subsequently modified them genetically using upcyte® technology to enable long-term culture and ongoing proliferation (designated up-LC14A1). The predictive transcriptomic classification clustered the individual cell line up-LC14A1 with HBV-replicating HepG2H1.3 cells, indicating the preservation of virus-associated molecular signatures. Although pathway enrichment and disease association analyses confirmed a strong alignment with liver cancer profiles, we demonstrate that up-LC14A1 cells maintain patient characteristics and dynamic HLA-G expression, both *in vitro* and *in vivo*.

Material and methods

Study approval, human material, and animal experiments

Human tissue and blood were collected, processed, preserved, and ethically approved as described previously[13,14] and listed in Table S1. HLA-A typing of patient- and healthy donor-derived tissue and blood cells was performed as described previously.[13] The study was approved by the Ethical Review Committee of the Ärztekammer Hamburg (PV-3578) and conducted in accordance with national guidelines and the 1975 Declaration of Helsinki. Animal experiments were performed in accordance with the European Communities Council Directive (86/EEC) and approved by the City of Hamburg, Germany (N056/2020). Mouse generation, breeding, housing, and surgical procedures were conducted as described previously.[14]

Generation of up-LC14 cells

Patient-derived HCC cells were isolated as described previously[14] and genetically modified to generate proliferation-competent cultures following established protocols for primary hepatocytes.[15,16] Cells were expanded under standard HCC culture conditions and cryopreserved for long-term storage. Detailed transduction, culture, and freezing procedures are provided in the Supplementary Methods.

Cell culture, genetic labeling, and spheroid formation

After thawing, up-LC14 cells were maintained in advanced DMEM/F-12-based culture conditions. Stable integration of the LeGO-iG2-Puro+-Luc2 construct by lentiviral transduction was performed as described previously.[14] Immortalized HCC lines (HUH-7, Hep3B, and HepG2-H1.3) were maintained as described previously.[17,18] For two-dimensional (2D) experiments, cells were seeded in 96- or 24-well plates and treated after 24 h. For three-dimensional (3D) assays, luciferase (LUC)-transduced up-LC14 cells were cultured in ultra-low attachment conditions to monitor spheroid formation over time. Full media compositions, seeding densities, siRNA transfection description, and imaging settings are provided in the Supplementary Methods.

NK92 co-culture experiments and real-time viability monitoring

NK92 cells were expanded in IL-2-supplemented culture conditions and applied to target cells at effector-to-target ratios of 1:1, 1:5, and 1:10. Supernatants and/or cells were collected at indicated time points for downstream analyses. Target-cell viability was monitored in real time using xCELLigence-based impedance measurements (ACEA Biosciences, 92121 San Diego, USA) . Detailed NK92 culture conditions and downstream readouts are provided in the Supplementary Methods.

Molecular analyses

We isolated RNA and DNA from cell lines and tissue using column-based kits.[13,14,19] Gene expression was quantified by TaqMan-based qPCR (Table S2) following cDNA synthesis. Whole-exome sequencing (WES) and RNA sequencing (RNA-seq) were performed on Illumina platforms (Illumina, San Diego, CA, USA) and analyzed using established pipelines; downstream analyses were conducted in R Studio version 4.5.3 (R Foundation for Statistical Computing, Vienna, Austria). Detailed library preparation, sequencing, and bioinformatic workflows are provided in the Supplementary Methods.

Flow cytometry, immunofluorescence, and protein measurements

Flow cytometry was performed using antibody panels listed in Tables S3 and S4.[14] Immunofluorescence staining of cultured cells and tissue cryosections was performed using primary antibodies listed in Table S5 and fluorescent secondary antibodies. We quantified sHLA-G (Thermo Fisher, Waltham, MA 02451, USA), human alpha-1 antitrypsin (AAT, abcam, Cambridge, MA 02139, USA), and perforin by ELISA according to manufacturers' instructions . Full assay conditions and reagent details are provided in the Supplementary Methods.

Viral DNA and cfDNA isolation and analysis

HBV DNA was quantified by TaqMan qPCR using cloned standards.[19] Cell-free DNA (cfDNA) was isolated from supernatants; fragment analyses were performed by Alu-based qPCR (Table S6) and locus-specific HLA-G assay (Table S2). Full assay conditions and reagent details are provided in the Supplementary Methods.

Statistical analysis

Graphs and statistical analyses were performed in GraphPad Prism version 10 (GraphPad Software Inc., La Jolla, CA, USA). Statistical tests and replicate numbers are detailed in the Supplementary Methods and/or figure legends. Significance thresholds were defined as *p <0.05, **p ≤0.01, ***p ≤0.001, and ****p ≤0.0001.

Results

Identification of the HLA-G genetic footprint reveals an immune-modulatory genomic profile regulated by HLA-G

To classify the patient material, we performed WES on two spatially separated regions of the resected tumor—the center and margin (Fig. 1A). Both regions showed highly overlapping

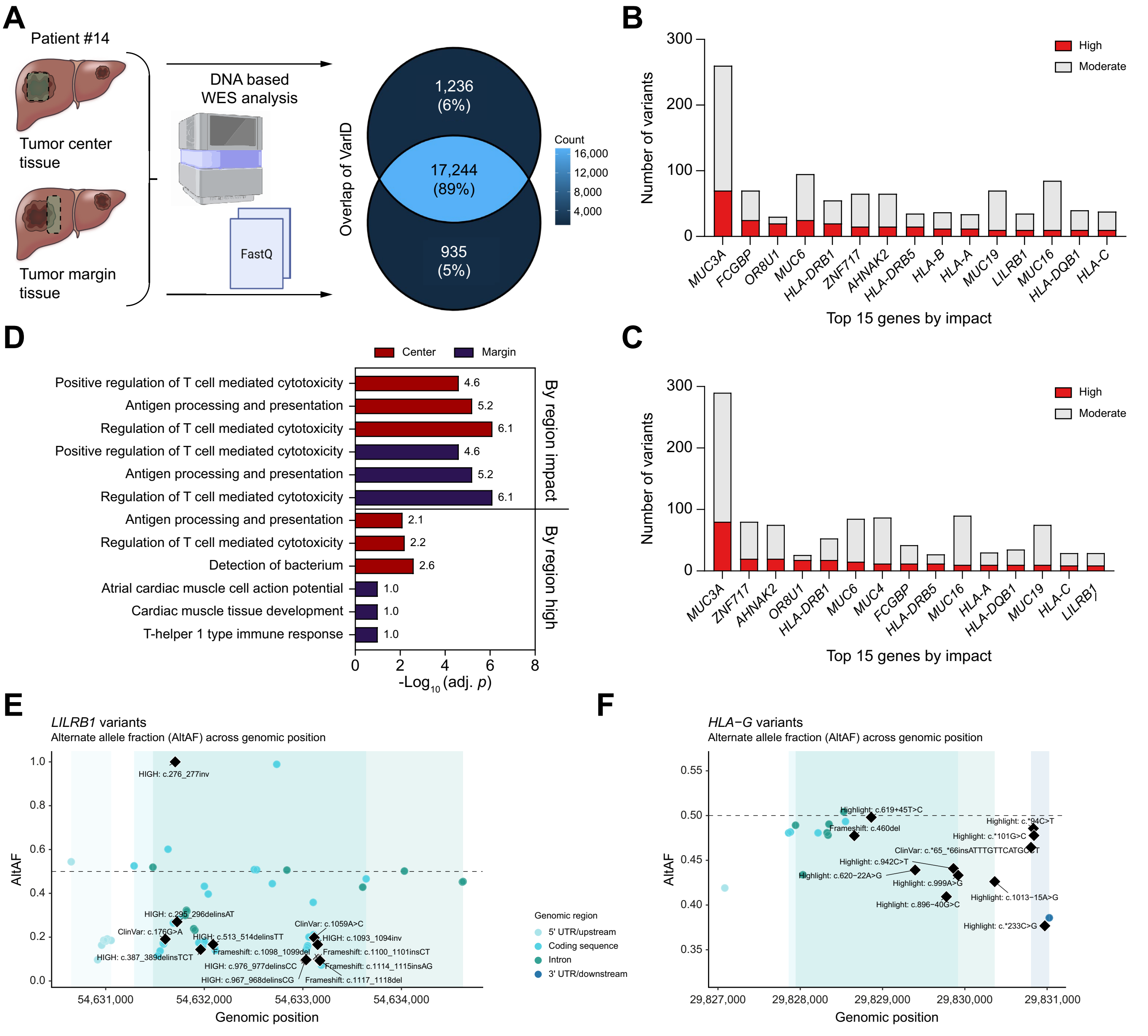

Fig. 1. Spatial whole-exome sequencing (WES) of patient's tumor center and margin tissue. (A) Spatial WES was performed on tumor center and margin from an HBV-associated hepatocellular carcinoma (HCC) resection (patient #14; n = 2 technical replicates/sample). Shared and region-specific variants are summarized by Venn diagram. (B) Top 15 variant-enriched genes are shown for (C) center and (D) margin, stratified by predicted impact (high, red; moderate, green). (D) Gene ontology biological process enrichment was conducted on genes harboring high/moderate variants; bars indicate -log10 adjusted p values. Variant positions and alternate allele fraction (AltAF) are shown for (E) *LILRB1* and (F) *HLA-G*, annotated by genomic context; dashed line denotes median AltAF.

variant profiles, indicating a shared clonal origin and enabling patient-level classification based on stable genomic features rather than spatial heterogeneity (Fig. 1A and Fig. S1). After filtering for putative functional variants (non-synonymous, high/moderate impact, variant allele frequency [VAF] ≥0.05), variant numbers, impact distributions, and VAF profiles were comparable between regions, consistent with malignant tissue of similar purity.

Genes with the highest variant burden were largely shared across regions (Fig. 1B,C). The top 15 variant-enriched genes included mucin-associated and structural genes (*e.g. MUC3A, MUC6, ZNF717,* and *AHNAK2*) and several immune-interaction genes within the HLA locus (*HLA-DQB1, HLA-DRB1/5,* and *HLA-C*) as well as the HLA-G receptor *LILRB1*. Despite minor ranking differences, the overall gene composition and the predominance of moderate-impact variants remained consistent, indicating that immune-interaction pathways represent a core genomic feature rather than a region-specific adaptation. Pathway enrichment of region-specific high-impact variants confirmed significant enrichment in antigen processing and presentation, and regulation of T-cell-mediated cytotoxicity (Fig. 1D).

We next examined variants in the *LILRB1* and *HLA-G* loci (Fig. 1E,F). The HLA-G locus contained the heterozygous 14-bp insertion (rs371194629), rs1063320 G, and rs1049033, variants associated with HBV susceptibility and HCC risk.[11,20] Additional polymorphisms (rs1130363, rs915667, rs1707, rs1710, and rs1610696) localize to the regulatory 3′UTR and form extended haplotypic blocks that affect transcript stability, microRNA binding, and the balance between sHLA-G and mHLA-G isoforms.[21] Together, the 14-bp insertion, rs1063320 G, and rs1610696 G alleles are most consistent with the canonical *HLA-G* 3′UTR UTR-2 haplotype—a regulatory configuration associated with immune tolerance and persistent viral infection.[22,23] This defines a patient-specific *HLA-G* haplotype compatible with enhanced immune-tolerogenic signaling.

Generation of a patient-derived HBV-HCC model with high HLA-G expression

We stratified the malignant material from patient #14 using diagnosed HCC tissue samples (in-house n13HCC cohort; n = 13; Table S1) for gene expression analysis. Across the plotted genes (Fig. 2A), patient #14 (red dot) aligned with the central distribution of the cohort, supporting its classification as representative HCC and providing a suitable basis for remodeling the immune-tolerogenic *HLA-G* haplotype. Next we analyzed serum samples from an overlapping cohort (n = 4 early-stage biopsies; n = 10 HCC; Table S1) for sHLA-G (Fig. 2B). Elevated sHLA-G levels were detected in patient #14 (red dot) compared with the dataset, consistent with the genetic phenotype.

To establish a functional patient-derived HCC cell line, parental liver cancer cells (p-LC14) were subjected to lentiviral transfer of the patented upcyte® gene cassette[15] (Fig. 2C). This enabled sustained *in vitro* proliferation that would otherwise be lost after short-term culture. After selective expansion, up-LC14A1 cells demonstrated stable proliferative capacity under standard conditions with consistent doubling times (Fig. S2A). In line with previously generated primary-like cell lines, up-LC14A1 cells showed a higher median doubling time (43 h) than immortalized cell lines (20 h).[14] In 2D culture, up-LC14A1 cells produced low levels of HBV DNA comparable to the patient's serum viral load at resection (Fig. 2D), in contrast to the high-producing HepG2H1.3 cells.

To enhance its utility as an *in vitro* and *in vivo* model, up-LC14A1 cells were retrovirally transduced with a LeGoVector expressing green fluorescent protein (GFP) and LUC (Fig. S2B). After puromycin selection, 67.7% of cells expressed GFP (up-LC14A1_LUC; Fig. S2C), as determined by flow cytometry. Following passage without further puromycin selection, GFP expression approached 100% as confirmed by fluorescence microscopy (Fig. S2D). Subsequently, up-LC14A1_LUC cells were used to assess spheroid formation (Fig. S2E). After 4 days in spheroid-supporting U-bottom plates, cells organized into 3D networks that increased in size over time. This spheroid-forming capacity enables modeling of tumor progression and therapeutic responses in a physiologically relevant *in vitro* setting.

To position up-LC14A1 cells relative to commonly used immortalized HCC cell lines, we applied our standard immunofluorescence characterization panel (Fig. 2E and Fig. S2F).[14] The up-LC14A1 cells showed high expression of CK19, calnexin, AFP, HNF4A, ACAD, vimentin, and actin. Low cytoplasmic expression was observed for hepatitis B surface protein, ALB, MICA/B, and CK7, with CD44 expression localized to the nucleus. The up-LC14A1 cells were negative for NTCP, EGFR, CD68, CD31, and PD-L1. Overall, up-LC14A1 cells retained the protein expression characteristics of p-LC14 cells as confirmed by flow cytometry (Fig. 2F), including surface presentation of HLA-G.

High *HLA-G* expression clinically correlates with reduced overall survival (Fig. S3G);[24,25] therefore, we analyzed *HLA-G* expression in parental tissue and cells derived from patient #14 using the n13HCC cohort supplemented with healthy donor samples (Fig. 2G and Table S1). Overall, no significant differences were observed between healthy and diseased samples, consistent with global datasets showing similar *HLA-G* expression distributions across early and advanced liver diseases (Fig. 2H). However, patient #14 tissue and up-LC14A1 cells exhibited elevated *HLA-G* expression above the median of the pathogen group (Fig. 2G). These expression levels correspond to the previously observed sHLA-G pattern (Fig. 2B) and indicate stable HLA-G gene and protein expression. The highly progressive and migratory characteristics of up-LC14A1 cells, combined with their consistent expression of HLA-G, potentially mimic the immune-tolerogenic nature of HBV-related liver cancer.

Transcriptomic profiling positions up-LC14A1 cells within the HCC landscape and reveals conserved hepatic and tumor-associated pathway activity

We analyzed the transcriptomic profile of up-LC14A1 cells to assess their clinical relevance and similarity to commonly used immortalized HCC cell lines. RNA-seq analysis included up-LC14A1, HepG2H1.3, Hep3B, HUH7, and primary human hepatocytes (PHH) (Fig. 3A). Across global differentially expressed gene (DEG) datasets, up-LC14A1 cells showed a high z-score activation match with cholangiocarcinoma, HCC, and hepatoblastoma, indicating preservation of the underlying clinical disease (Fig. 3B). Variance-stabilized datasets from HCC cell lines and up-LC14A1 cells were subjected to DEG

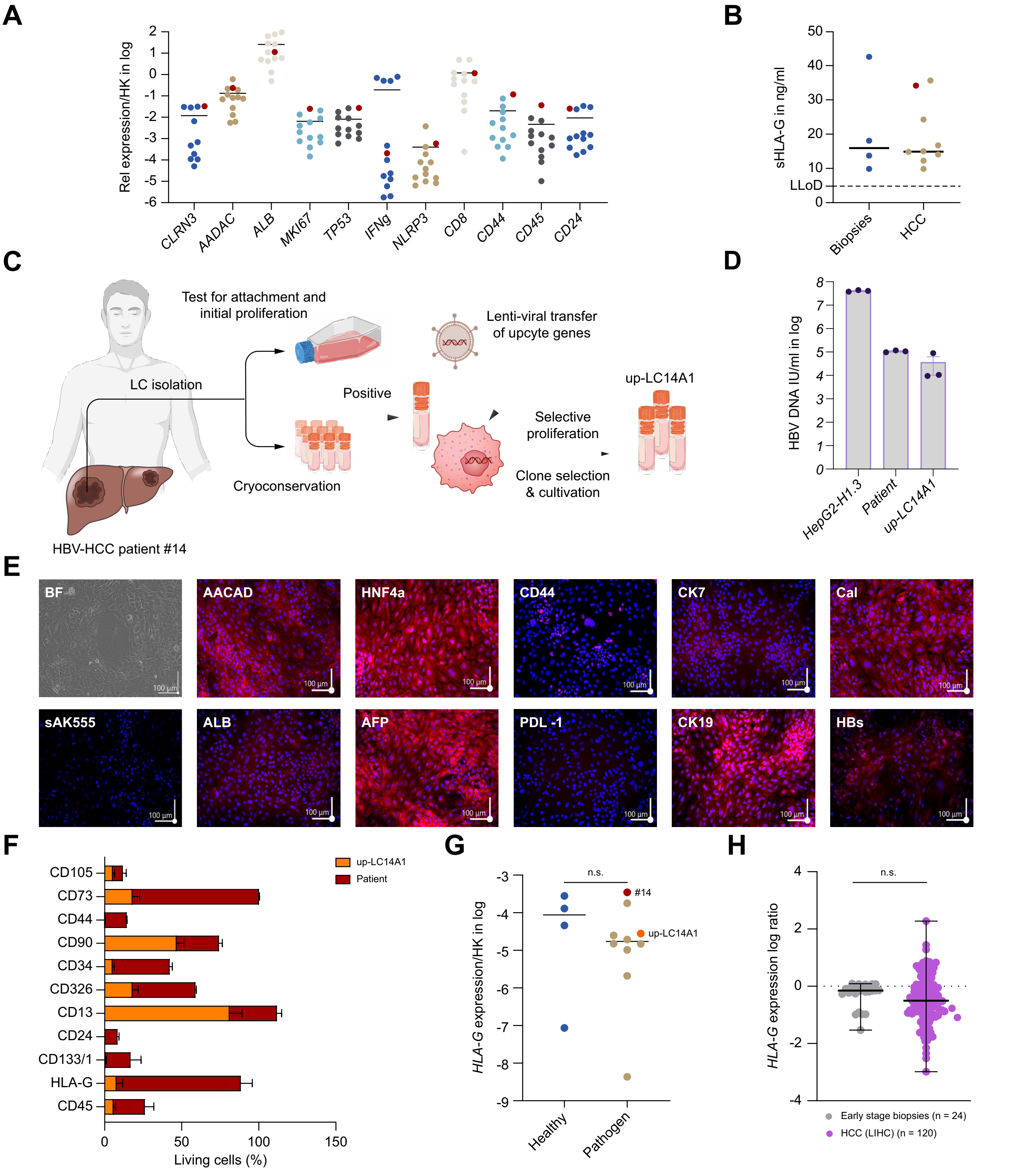

Fig. 2. Generation and characterization of the patient-derived HBV-associated hepatocellular carcinoma (HCC) cell line up-LC14A1. (A) Generation and characterization of the patient-derived HBV-associated HCC cell line up-LC14A1. Gene expression of selected classifier genes in patient #14 relative to n = 13 patient-derived HCC samples. (B) Serum soluble HLA-G (sHLA-G) levels in early-stage biopsies (n = 4) and diagnosed HCC (n = 9; lower limit of detection 4.9 ng/ml); patient #14 highlighted in red. (C) Workflow for LC14 isolation and upcyte® cassette transfer, clonal selection, and establishment of up-LC14A1. (D) HBV DNA in supernatants and patient serum (day of resection) including HepG2-H1.3 control (n = 3). (E) Immunofluorescence markers; Hoechst nuclei; scale bar: 100 µm. (F) Flow cytometry marker frequencies (n = 3). (G) *HLA-G* expression (healthy n = 4; patient material n = 9; up-LC14A1). (H) TCGA *HLA-G* (biopsies n = 24; HCC n = 120).

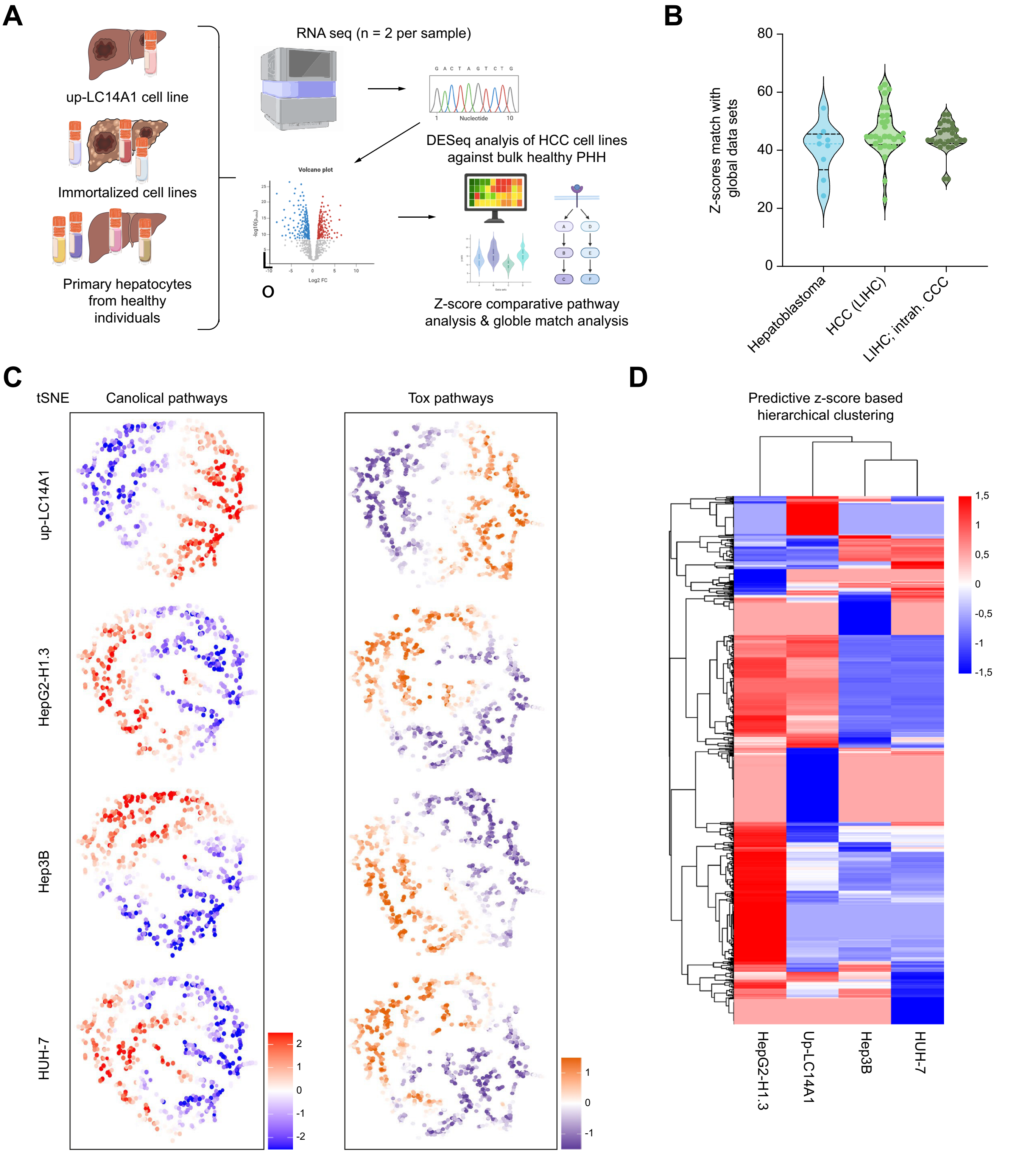

Fig. 3. Transcriptomic classification of up-LC14A1 cells normalized against primary hepatocytes in a global context. (A) Transcriptomic classification of up-LC14A1 relative to primary human hepatocytes (PHH). RNA sequencing (RNA-seq) was performed for up-LC14A1, established HCC lines, and PHH (n = 2 technical replicates/sample). (B) Differential expression *vs*. PHH was followed by pathway and global similarity analyses using z-scores. Ingenuity Pathway Analysis (IPA)-based similarity of canonical/toxic pathways is shown as z-scores (values <10 excluded); violin plots summarize match scores across reference diagnoses. (C) Pathway activity was visualized by t-distributed stochastic neighbor embedding (t-SNE) for up-LC14A1, HepG2-H1.3, Hep3B, and HUH-7; colors indicate z-scored activity. (D) Hierarchical heatmap shows canonical pathway z-scores (red up, blue down; range -1.5 to 1.5).

analysis using t-distributed stochastic neighbor embedding (Fig. 3C). Analyzed genes were associated with canonical signaling pathways as well as stress- and toxicity-related programs. Dimensionality reduction generated shared gene-level coordinates across cell lines, onto which relative expression differences were projected. Across canonical pathways, up-LC14A1 cells displayed activation patterns overlapping with established HCC lines, indicating preservation of core tumor-associated signaling programs. A similar distribution was observed for toxicity- and stress-related pathways, with up-LC14A1 mapping within the transcriptional space of reference HCC models rather than forming a distinct cluster. These findings indicate that up-LC14A1 recapitulates the global pathway architecture of HCC while retaining patient-specific transcriptional features.

For predictive pathway analysis, we evaluated normalized datasets using PHH as reference. Hierarchical clustering of canonical pathway activation (positive z-scores) and inhibition (negative z-scores) is shown in Fig. 3D. HUH7 and Hep3B exhibited high similarity in pathway regulation, whereas virus-producing HepG2H1.3 showed a more distinct profile. The pathway regulation pattern of up-LC14A1 partially overlapped with both HepG2H1.3 and Hep3B, suggesting infection-related pathway adaptations and similarities to HBV-associated HCC models. Principal component analysis (Fig. S3A and B) confirmed these relationships. HUH7 (FC43) and Hep3B (FC45) clustered closely, whereas HepG2H1.3 (FC44) displayed a distinct pathway regulation pattern. However, up-LC14A1 cells (FC33) occupied an intermediate position between non-HBV-producing lines (HUH7, Hep3B) and the HBV-producing HepG2H1.3 line. DEG analysis showed reduced *HLA-G* expression across all investigated cell lines; however, among the pathogenic lines, HepG2H1.3 exhibited the highest levels compared with PHH (Fig. S3C).

The individual gene regulation landscape of up-LC14A1 is illustrated in a volcano plot (Fig. S4A). Upregulated top genes (-log10 adjusted p value >200) were associated with biological regulation (six genes), cellular processes (15 genes), metabolic processes (eight genes), and responses to stimuli (six genes). Elevated expression of *HSPB1* and *EPAS1* suggested increased stress resistance, whereas *TIMP1, SERPINA1, GPNMB,* and *IL1R1* indicated an immune-modulatory phenotype. Notably, strong induction of *SERPINA1* supports the use of human AAT as an *in vivo* serum marker. Regulation of *ALAS1, COX4, and SCARB1* further indicated increased metabolic activity. The most prominently downregulated genes were associated with biological regulation (19 genes), cellular processes (34 genes), and metabolic processes (22 genes), including impaired mTOR and MAP kinase signaling. Detailed canonical pathway analysis (Fig. S4B), compared with PHH, showed downregulation of complement cascade, interferon (IFN) signaling, and mitochondrial translation pathways. In contrast, pathways related to protein ubiquitination, mitotic metaphase, neddylation, L1CAM interactions, and cell cycle checkpoints were upregulated.

The up-LC14A1 cells better mimic HCC progression in xenograft mice than commonly used immortalized HCC cell lines

We evaluated the *in vivo* suitability of up-LC14A1 cells using orthotopic liver transplantation into immunodeficient mice (Fig. 4A). Representative images of chimeric livers are shown in the right panel. For comparison, HUH7, Hep3B, and HepG2H1.3 cells were transplanted under identical conditions. All three immortalized lines demonstrated strong tumorigenic persistence (Fig. S5A), with HUH7 showing pronounced necrotic liver areas. In contrast, although Hep3B and HepG2H1.3 induced fewer necrotic structures, animals reached ethical endpoints within 4 weeks owing to rapid deterioration of body condition. Mice transplanted with up-LC14A1 cells did not meet stop criteria before Week 12 (Fig. S5B), indicating slower progression without excessive health burden. This pattern supports a stemless tumorigenic growth leading to structured chimeric livers (Fig. S5C). Accordingly, up-LC14A1-transplanted mice were sacrificed after 12–14 weeks, and livers were analyzed by flow cytometry in comparison with p-LC14 cells (Fig. 4B). The livers repopulated with up-LC14A1 cells displayed a minor portion of ki67- and CD44-positive cancer cells (Fig. S6). After *in vivo* repopulation, up-LC14A1 cells express higher levels of HLA-G compared with the p-LC14 cells (Fig. 4B). Notably, markers that were absent in up-LC14A1 cells in conventional 2D culture, such as CD13, CD44, and CD24, were detected after expansion in the liver of immunodeficient mice. Taken together, we detected a higher cancer stem cell-like population in the up-LC14A1 cell line when compared with the p-LC14 cells isolated after resection and pretreatment. Serum analysis demonstrated highest levels of AAT (*SERPINA1*) in HUH7-transplanted mice, but low AAT levels in Hep3B-transplanted mice (Fig. 4C). Furthermore, AAT levels in HepG2H1.3- and up-LC14A1-transplanted mice were comparable. Considering tumor progression in the liver, up-LC14A1 induced moderate AAT secretion relative to immortalized HCC lines, whereas HUH7 produced markedly elevated levels consistent with aggressive, metastatic growth and the necrotic liver structures observed histologically (Fig. S5A). Intrahepatic RNA expression of *STAT3*, *CD44, HNF4A*, and *HMG-CoA reductase* (*HMG-CoAR*) showed pronounced heterogeneity (Fig. 4D). In line with others, we found high *STAT3* expression levels in the HBV-related cell lines Hep3B, HepG2H1.3, and up-LC14A1.[26] Hep3B-transplanted livers displayed the highest *CD44* expression, whereas HepG2H1.3- and HUH7-transplanted livers predominantly displayed *HNF4A* and *HMG-CoAR* expression, respectively. In contrast, up-LC14A1-transplanted livers displayed moderate expression across all human-specific markers. Histological analysis confirmed structured infiltration and integration of up-LC14A1 cells into murine liver tissue, forming human cancer cell-derived chimeric livers (Fig. 4D). The up-LC14A1 cells were CD73 positive, consistent with moderate engraftment kinetics. Distinct infiltration fronts and clear tumor–host borders were visible (Fig. 4E); moreover, HLA-ABC-positive up-LC14A1 cells migrated through murine biliary epithelial channels (Fig. 4F), indicating defined routes of repopulation and progression.

The up-LC14A1 cells displayed immune-tolerogenic dynamics, whereas immortalized HepG2H1.3 cells exhibited a static phenotype

As determined by DEG analysis, HepG2H1.3 cells expressed HLA-G mRNA at higher levels than did HUH7, Hep3B, and up-LC14A1 cells. As demonstrated in a previous study,[14] all three

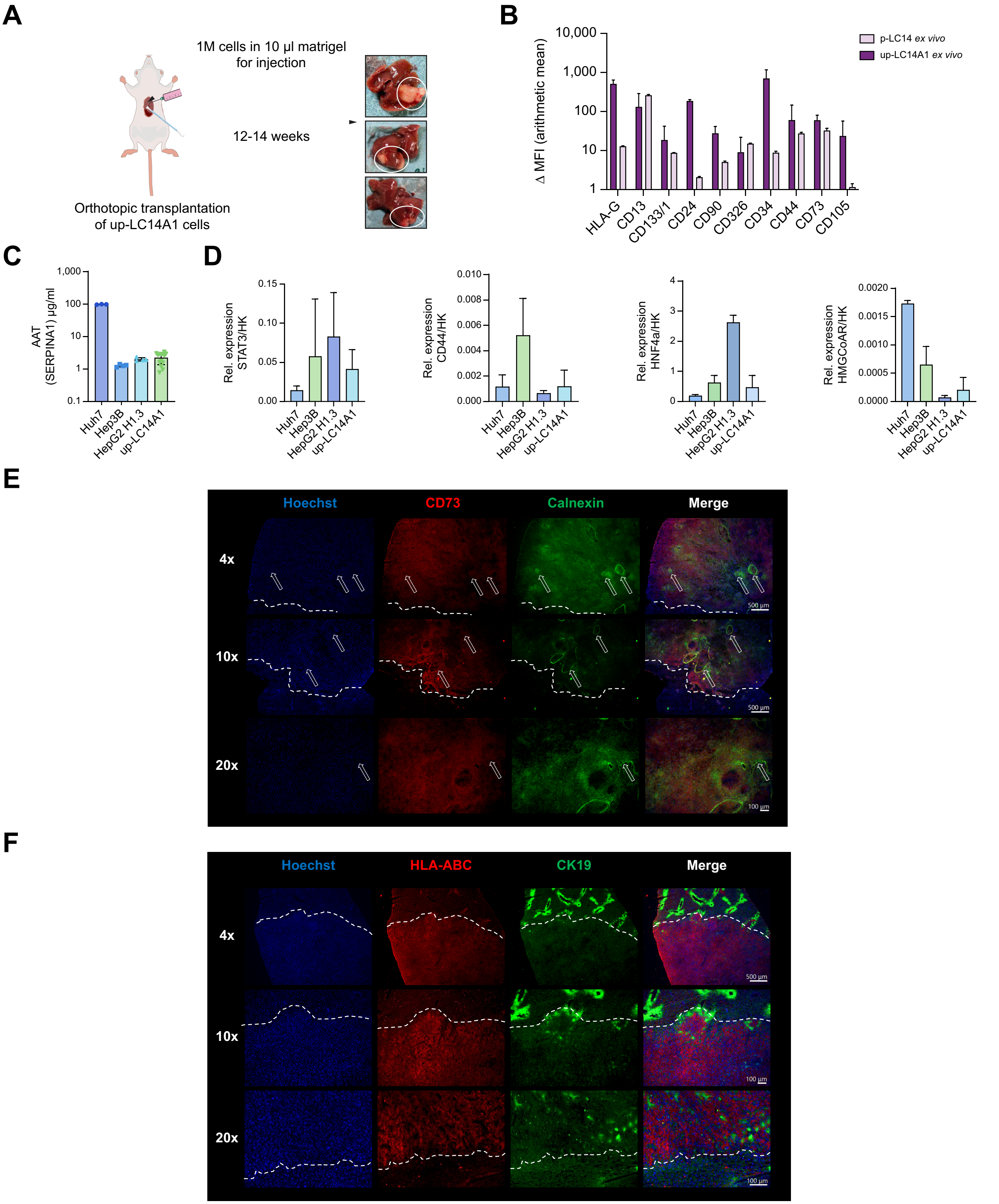

Fig. 4. Serological and intrahepatic analysis of up-LC14A1 orthotopically transplanted immune-deficient mice. (A) Experimental design and representative chimeric livers after orthotopic transplantation of up-LC14A1 into immunodeficient mice. (B) The up-LC14A1 cells were re-isolated from repopulated livers (n = 3 mice) and analyzed by flow cytometry. Mean fluorescence intensity difference (ΔMFI) of indicated markers is shown for parental cells (p-LC14 *ex vivo*; n = 3 technical replicates) and engrafted up-LC14A1 cells (n = 3 biological replicates). (C) Serum alpha-1 antitrypsin (AAT) levels (µg/ml). (D) Intrahepatic gene expression of indicated genes ($\log_{10}$, normalized to housekeeping genes). (E, F) Immunofluorescence images showing tumor infiltration fronts (arrows) and tumor–host borders (lines) at different magnifications; scale bars indicated.

immortalized cell lines, as well as up-LC14A1 cells, present HLA-G protein on their surface (Fig. 2C). However, to determine immunotolerance induction, we used HepG2H1.3 cells. Their high HLA-G mRNA levels, consistent HBV production, DEG-based pathway clustering, and corresponding *HLA-G* 3′UTR UTR-1 haplotype make them a suitable model for a comparative investigation with up-LC14A1 cells.[27] Therefore, immortalized NK92 cells were transferred into a 2D co-culture with different target-to-effector (T: E) ratios for 4 days (Fig. 5A). Consequently, HepG2H1.3 cells were significantly reduced in cell viability (Fig. 5B) regardless of the effector-to-target ratio, whereas up-LC14A1 cells displayed a clear adaption towards the immune NK92 cell treatment (Fig. 5C). On Day 2, 1:5 and 1:10 treatment regimens succeeded to reduce the proliferation rate and induced apoptosis; however, this effect was abrogated at 1:5 ratio on Day 4 totally. This finding contrasted with the cytotoxic active status of NK92 cells determined by the ratio, depending on linear detection of perforin in the supernatant (Fig. 5D). Moreover, in 1:1 T:E co-culture, NK92 cells were not sufficient to induce cytotoxicity. In contrast, NK92 cells effectively lysed HepG2H1.3 cells also in the 1:1 T:E setting. Nevertheless, over time in a 1:1 condition, HepG2H1.3 cells exhibited increased viability, likely because of the short lifespan of NK92 cells.

To elucidate the underlying cell death mechanism, we analyzed the occurrence of cfDNA-based long (247 bp) and short (115 bp) *ALU* fragments in the supernatant of indicated conditions (Fig. 5E). In both co-culture settings, we observed higher levels of short cfDNA fragments than long fragments, resulting in the low DNA integrity coefficients shown in Fig. 5E and providing further evidence that cell death was induced by apoptosis.[28] In parallel, cfDNA was used for locus-specific analysis of *HLA-G* (Fig. 5F), revealing distinct release patterns between HepG2H1.3 and up-LC14A1. HepG2H1.3 displayed significantly higher basal *HLA-G*-positive cfDNA, whereas up-LC14A1 exerted low baseline levels, consistent with their distinct HLA-G regulatory states (*HLA-G* haplotypes). For HepG2H1.3 cultures, *HLA-G*-positive cfDNA levels correlated with overall cfDNA release and cytotoxicity, suggesting that locus-specific detection largely reflected passive DNA release associated with cell turnover (Fig. 5F). In contrast, under low cytotoxic pressure (1:1 effector-to-target ratio), up-LC14A1 cells exhibited a relative increase in *HLA-G* locus-positive cfDNA that could not be explained by apoptosis-associated DNA release alone. Notably, under conditions of stronger NK92-mediated cytotoxicity, *HLA-G*-positive cfDNA decreased despite increased apoptosis and reduced viability (Fig. 5C,F). Taken together, our findings indicate that, for up-LC14A1 cells, the *HLA-G* locus cfDNA does not scale proportionally with cell death but instead reflects the release of *HLA-G*-containing DNA fragments from viable tumor cells under immune pressure, as part of tumor–immune communication.[29]

The intracellular gene expression pattern (Fig. 5G) confirmed this irregular pattern between HepG2H1.3 and the immune-tolerogenic up-LC14A1 cells. Interestingly, the low *HLA-G*-expressing up-LC14A1 cells increased expression by 20-fold within 24 h after the application of NK92 cells, whereas this dynamic was not observed in HepG2H1.3 cells. This finding of dynamic and static reactions to NK92 cell transfer was consistent with the expression patterns of both cell types and with the *HLA-G* haplotype of up-LC14A1 cells. Cytometric analysis of NK92 cells on Day 4 showed increased CXCR4 and CCR7 expression under 1:10 conditions, accompanied by downregulation of activation markers NKp30 and NKp46 (Fig. 5H). This pattern correlated with the enhanced killing observed at the higher effector-to-target ratio, indicating effective target cell elimination at 1:10 compared with 1:1 condition. Overall, NK92 cells shifted from a predominantly cytolytic toward a more migratory phenotype, demonstrating phenotypic adaptation of this immortalized NK cell line in response to dynamic, immune-tolerogenic target cell interactions.

HLA-G knockdown reduces immune tolerance of up-LC14A1 cells and forces the switch to PDL-1 inhibition

We assessed the immune regulatory role of HLA-G in up-LC14A1 cells by siRNA-mediated HLA-G silencing followed by NK92 co-culture (Fig. S7A). All three siRNAs significantly reduced *HLA-G* expression compared with control conditions, with siRNA1 producing the strongest knockdown (Fig. 6A). Longitudinal viability measurements confirmed NK92-mediated cytotoxic pressure and revealed reduced immune tolerance after HLA-G silencing (Fig. 6B,C). At effector-to-target ratios of 1:5 and 1:10, *HLA-G* knockdown consistently led to a stronger decline in cell index, accompanied by significantly increased perforin secretion, indicating enhanced cytolytic activation (Fig. 6D). Phenotypic profiling supported this effect. The CD56dim/CD16low compartment shifted toward a PD-1+/Lag3– composition across conditions, with the highest proportion of CD56dim/CD16low/NKp46–/CD226+/PD-1+/Lag3– cells observed after siRNA1 treatment, which also produced the strongest cytotoxic impact (Fig. 6B,C,E). Analysis of HLA-G receptors within this dominant NK92 subset showed that HLA-G reduction promoted a shift toward ILT2/ILT4 double-negative cells, whereas HLA-G-expressing conditions showed modest ILT2 induction (Fig. 6F). Effector–target interaction generally led to strong ILT4 downregulation, whereas siRNA1 caused the most pronounced decrease in ILT2 expression alongside the highest frequency of this activated subset. KIR2DL4 co-expression with ILT2 and/or ILT4 was detected under baseline conditions. Under siRNA1, 34% of ILT2/ILT4 double-positive and 8% of ILT2-positive cells retained KIR2DL4 (Fig. S7B). Immunofluorescence confirmed strong HLA-G protein induction after NK92 transfer, which was markedly reduced by siRNA treatment, most prominently with siRNA1 (Fig. 6G). Concomitantly, PD-L1 expression was strongly upregulated under HLA-G knockdown, with weaker induction in siRNA2 and siRNA3 conditions. Together, these data indicate that HLA-G suppression enhances NK92-mediated cytotoxicity while triggering compensatory PD-L1-mediated immune evasion, highlighting a dynamic switch between immune regulatory pathways.

HLA-G is localized at target–effector contact sites, consistent with contact-dependent immune modulation

We analyzed the spatial relationship between HLA-G-expressing up-LC14A1 cells and NK92 cells by immunofluorescence microscopy (Fig. 7). Up-LC14A1 cells exhibited strong membranous and cytoplasmic HLA-G expression (cyan), whereas NK92 cells were identified by CD45 expression (purple). The co-culture resulted in the formation of dense cellular clusters in which NK92 cells accumulated around HLA-G-presenting up-LC14A1 cells, pointing to active immune–tumor cell interaction. In high-magnification views,[1–5] multiple

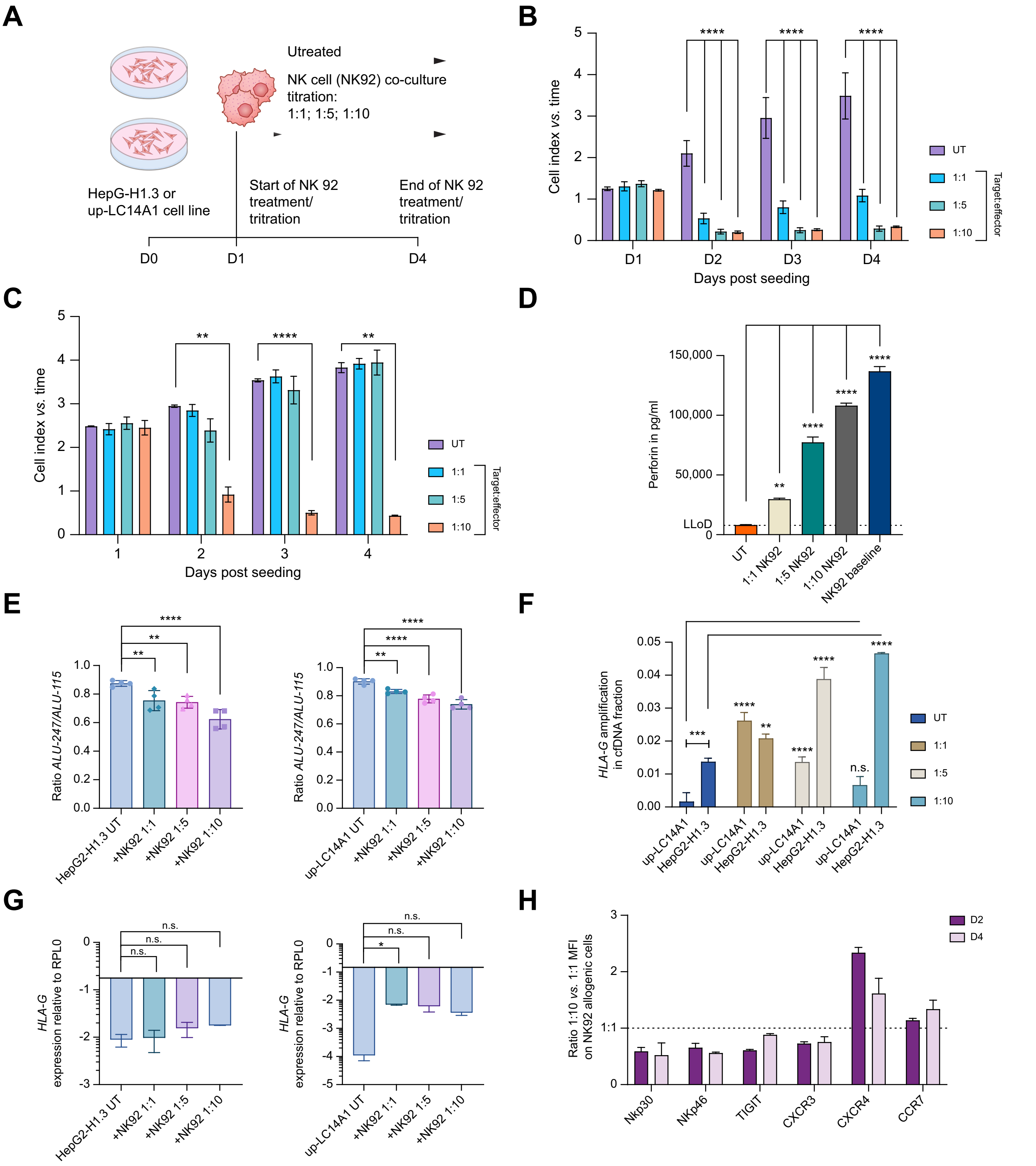

Fig. 5. NK92 cell transfer reveals the adaptive immune-evasive phenotype of up-LC14A1 cells. (A) NK92 co-culture reveals adaptive immune evasion of up-LC14A1. Experimental scheme is shown. Longitudinal xCELLigence cell index is shown for (B) HepG2-H1.3 and (C) up-LC14A1 across indicated effector-to-target (E:T) ratios (n = 2 × 5 replicates/condition). (D) Perforin in supernatants is quantified (pg/ml, linear scale). (E) cfDNA fragmentation was assessed by *ALU-115/ALU-247* quantification and ratio analysis (n = 4), and (F) *HLA-G* gDNA in cell-free DNA (cfDNA) was quantified relative to RPL0 (n = 4). (G) Intracellular *HLA-G* mRNA after 24 h co-culture is shown (n = 4 biological replicates). (H) Flow cytometry compares marker fold-changes at high (1:10) *vs.* low (1:1) E:T on Days 2 and 4.

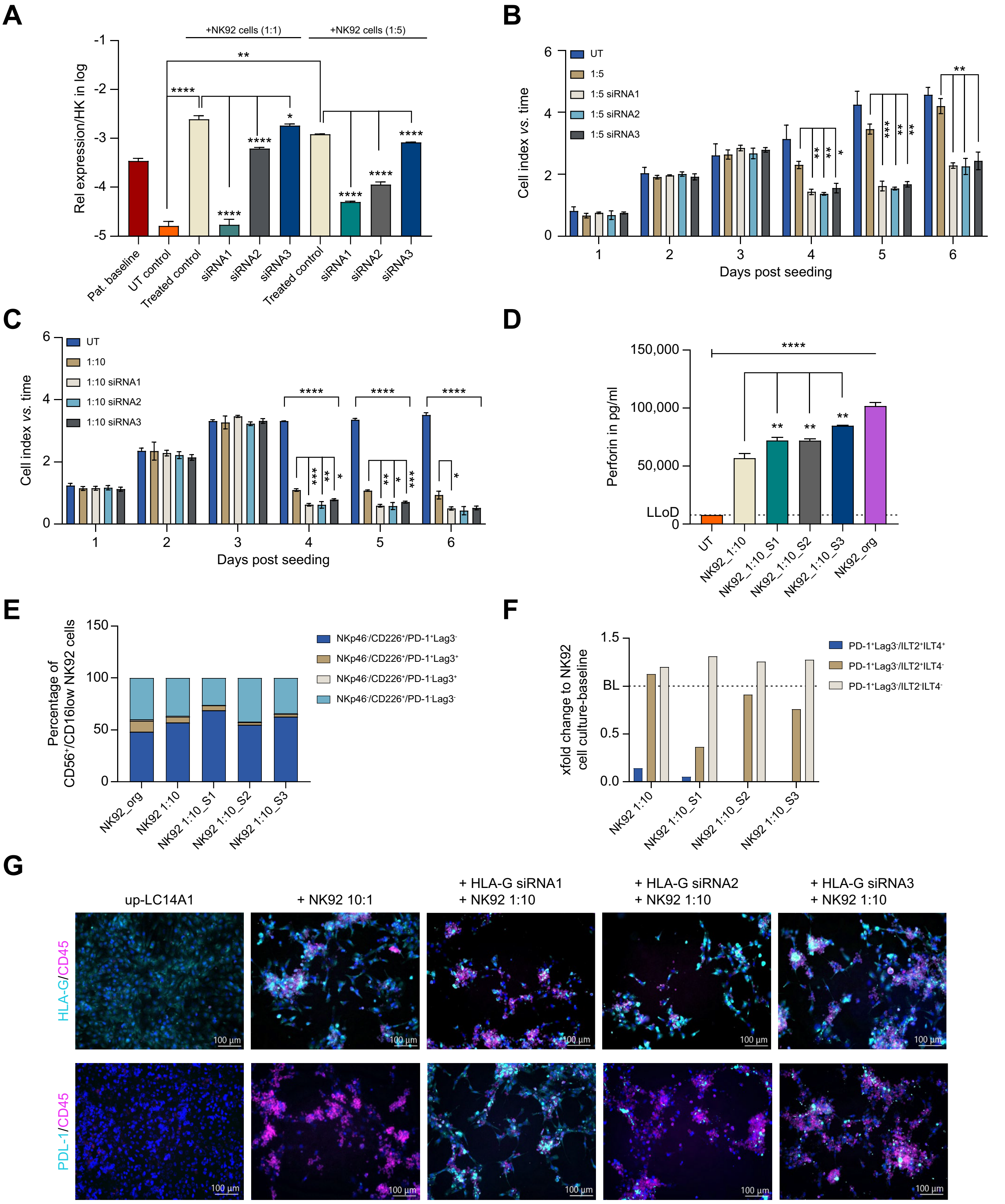

Fig. 6. Functional effects of HLA-G silencing on NK92 cell responses toward up-LC14A1 cells. (A) Functional impact of HLA-G silencing on NK92 responses to up-LC14A1. HLA-G knockdown by siRNA in up-LC14A1 was quantified by normalized HLA-G mRNA under NK92 co-culture at indicated ratios (1:1, 1:5). (B, C) Longitudinal xCELLigence cell index of up-LC14A1 is shown for indicated conditions. (D) Perforin in supernatants after 72 h NK92 co-culture is shown (pg/ml; lower limit of detection indicated; percentages shown per condition). (E) Frequencies of CD16-dim and CD16low within CD56dim NK92 cells are shown. (F) PD-1/LAG-3 distribution is shown for CD56+/CD16low/NKp46–/CD226+ NK92 cells. (G) ILT2/ILT4 changes are shown as fold-change *vs.* baseline (BL) at 27 h (E:T 1:10). (H) Immunofluorescence at 48 h shows HLA-G or PD-L1 (aqua) with CD45 (purple) and Hoechst nuclei; scale 100 µm.

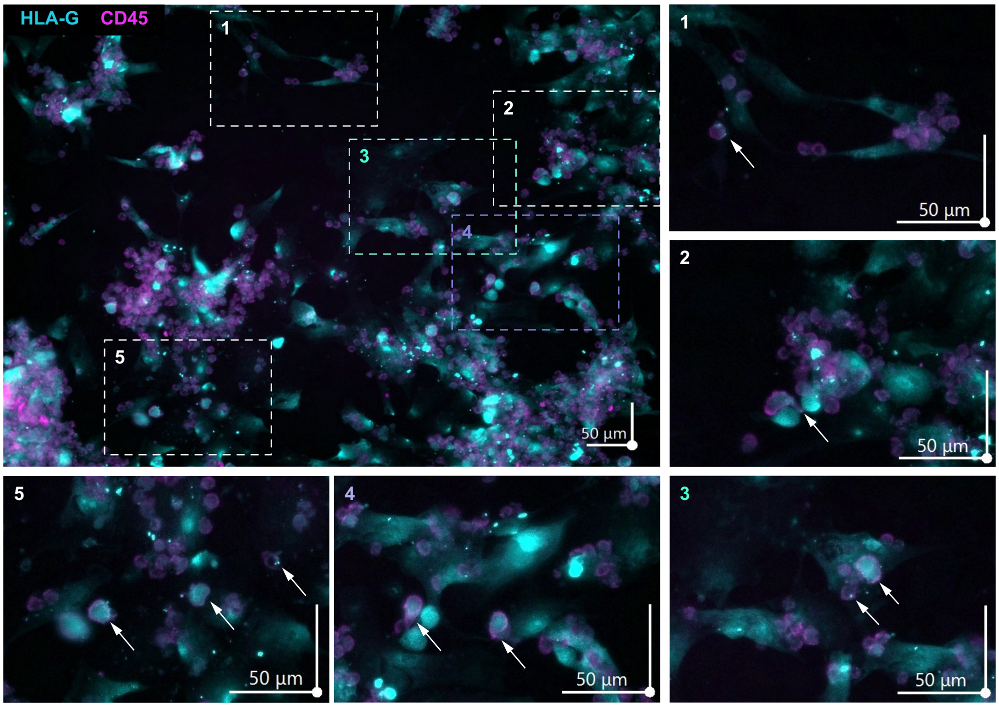

Fig. 7. Membrane-bound HLA-G transferred on NK92 cells showing dynamic immune tolerance pathways of up-LC14A1 cells. Representative immunofluorescence images show HLA-G (cyan) on up-LC14A1 tumor cells and its redistribution to interacting NK92 cells identified by CD45 (magenta). Overview images indicate NK92 accumulation at HLA-G-expressing tumor surfaces; dashed boxes mark regions shown at higher magnification (panels 1–5). Arrows highlight tumor–NK contact sites and HLA-G signals on NK92 membranes after interaction, consistent with contact-dependent transfer. These findings support the formation of HLA-G-mediated immune regulatory interfaces compatible with local tolerance. Scale bars: 50 µm.

direct contact zones between NK92 cells and up-LC14A1 cells are highlighted. At the interfaces, HLA-G signal was frequently concentrated at the contact sites and, in several instances, appeared at the surface of adjacent CD45^{+} NK92 cells (white arrows). These observations suggest that HLA-G is actively engaged at the immune synapse and redistributed during these target–effector interactions.

Discussion

Chronic HBV infection is a major global driver of HCC, accounting for up to 50% of cases and representing a key factor in inflammation-associated hepatocarcinogenesis.[1–3,5,30] In this setting, the non-classical MHC class I molecule HLA-G has emerged as a potent immune checkpoint that suppresses cytotoxic lymphocyte activity through inhibitory receptors ILT2 and ILT4 on NK cells, T cells, and myeloid cells.[6,7,9] By attenuating cytotoxicity, cytokine release, and immune synapse formation, HLA-G promotes immune tolerance within the tumor microenvironment.[6,7,9,31] In chronic HBV infection, miR-152 downregulation increases HLA-G expression, impairing NK-cell responses and linking viral persistence to HLA-G-mediated immune escape.[10]

HLA-G can be present either in mHLA-G or sHLA-G form.[7] In the clinical setting, elevated sHLA-G levels predict poor overall survival in patients with virus-induced HCCs, alcohol-associated liver disease (ALD), and metabolic dysfunction-associated steatotic liver disease/steatohepatitis (MASLD/MASH) entities.[32] Moreover, serum sHLA-G levels are associated with clinical phases of chronic HBV infection and correlate with viral persistence and immune tolerance, supporting the role of HLA-G in shaping the host-virus equilibrium.[6,8,33–35] More recently, combined analyses of HLA-G expression and regulatory polymorphisms, including 3′UTR variants, have linked high sHLA-G levels to adverse clinical outcomes across viral and metabolic HCC entities.[32,36]

In the present study, we established a patient-derived HBV-HCC model (up-LC14A1) that retains key features of the parental tumor. Stratification of parental tumor tissue and serum reveals typical HCC characteristics. Spatially resolved WES of the parental tumor tissue revealed a clonally coherent mutational landscape enriched for antigen presentation and immune-interaction genes. Notably, the malignant tissue carried a regulatory *HLA-G* 3′UTR-2 haplotype, which is reportedly associated with HBV susceptibility and immune-tolerogenic processes,[11,20–22] and therefore appears as driver of immune modulation in this patient. In this study, we showed that up-LC14A1 cells capture a stable, patient-specific immunotolerogenic configuration rather than a stochastic adaptation. Importantly, HLA-G expression is known to be influenced by both genetic variants and inflammatory cues, highlighting the interplay between clonally encoded predisposition and inducible immune regulation.[6,7]

Phenotypically, up-LC14A1 cells present hepatobiliary lineage markers, including CK19, AFP, and HNF4A, confirming their origin from HBV-associated liver cancer. They also express HLA-G and CD73, pointing to an immune-evasive and progenitor-like phenotype. CD73 is a key component of the adenosine pathway, generating extracellular adenosine that suppresses NK-cell activity and promotes tumor–immune escape.[7,37] Expression of CD73 in HCC has been associated with stem-like properties, therapy resistance,

and immunosuppression, supporting the relevance of this phenotype for disease progression and treatment resistance.[7] By orthotopic transplantation, up-LC14A1 cells displayed gradual integration into the murine liver and non-necrotic tumor growth, in contrast to the rapid, often necrotic expansion of commonly used immortalized HCC cell lines. This slower progression resulted in the formation of chimeric livers and prolonged the survival of transplanted animals, indicating a more gradual tumor expansion within this experimental setting.

Transcriptomic profiling placed up-LC14A1 cells between HBV-producing (HepG2H1.3) and non-producing (Hep3B, HUH7) HCC lines, consistent with a hybrid, virus-associated tumor state. Functionally, the model showed dynamic immune tolerance: under NK92 pressure, HLA-G was rapidly induced and enriched at tumor–immune contact sites. Its presence at effector-cell interfaces aligns with reported trogocytosis-mediated transfer of HLA-G from tumor to NK92 cells, which can convert cytotoxic lymphocytes into regulatory cells and promote immune escape.[38–40] This spatial redistribution supports contact-dependent suppression within the tumor–immune synapse.[7] We further detected *HLA-G*-containing DNA fragments released from viable tumor cells under immune pressure, suggesting a role in tumor–immune communication.[29]

Silencing HLA-G restored NK92 cell cytotoxicity and triggered compensatory PD-L1 upregulation, indicating that HLA-G is part of the hierarchical checkpoint network.[41] Interconnected immune checkpoint pathways have been described across multiple tumor types, in which inhibition of one suppressive axis results in the activation of alternative pathways to maintain immune escape.[41,42] Interferon-γ (IFN-γ) signaling from activated NK or T cells is a plausible mechanism for PD-L1 induction in this setting, as IFN-γ is a canonical driver of PD-L1 expression on tumor cells and contributes to adaptive immune resistance.[43] These observations support a model in which HLA-G functions as a central but dynamic node within an adaptive immune regulatory circuit rather than as a static inhibitory molecule.[44]

In summary, our data place HLA-G within a clonally encoded yet dynamically regulated immune tolerance program in HBV-associated HCC. The up-LC14A1 model provides a platform to dissect how genomic background, viral context, and immune pressure jointly shape immune escape and therapy resistance—a key challenge for emerging HCC immunotherapies.[1,3]

A limitation is the use of upcyte® technology, which confers proliferative capacity while preserving primary-like features but may alter regulatory pathways.[15,16] Although we confirm parental characteristics to a relevant extent, future studies using patient-derived NK and T cells are required to validate and extend HLA-G-mediated tolerance mechanisms.

Overall, our findings support a role of HLA-G in immune evasion through both genetically encoded predisposition and inducible regulation under immune pressure. The up-LC14A1 model provides an experimental platform to investigate how genomic background, viral context, and immune pressure contribute to immune escape in HBV-associated HCC.

Affiliations

[1]*Department of Medicine, University Medical Center Hamburg-Eppendorf, Hamburg, Germany;* [2]*Faculty of Health Sciences Brandenburg, Brandenburg Medical School Theodor Fontane, Brandenburg, Germany;* [3]*Department of Gastroenterology, Diabetology and Hepatology, University Hospital Brandenburg, Brandenburg Medical School Theodor Fontane, Brandenburg, Germany;* [4]*Center for Translational Medicine, Brandenburg Medical School Theodor Fontane, Brandenburg, Germany;* [5]*Brandenburg University of Technology Cottbus-Senftenberg, Institute of Biotechnology, Senftenberg, Germany;* [6]*German Center for Infection Research, Hamburg-Lübeck-Borstel Partner Site, Germany;* [7]*Department of General, Visceral and Thoracic Surgery, University Medical Center Hamburg-Eppendorf, Hamburg, Germany;* [8]*Institute of Clinical Genetics, Faculty of Health Sciences Brandenburg, Brandenburg Medical School Theodor Fontane, Brandenburg, Germany*

Abbreviations

2D, two-dimensional; 3D, three-dimensional; AAT, alpha-1 antitrypsin; AltAF, alternate allele fraction; cfDNA, cell-free DNA; DEG, differentially expressed gene; GFP, green fluorescent protein; HCC, hepatocellular carcinoma; HLA, human leukocyte antigen; ICI, immune checkpoint inhibitor; IPA, Ingenuity Pathway Analysis; LC14/(p-LC14), (parental) liver cancer cells 14; LeGO, LeGO vector system; LUC, luciferase; MHC, major histocompatibility complex; mHLA-G, membrane-bound HLA-G; NK cells, natural killer cells; PD-1, programmed cell death protein 1; PD-L1, programmed death-ligand 1; PHH, primary human hepatocytes; RNA-seq, RNA sequencing; sHLA-G, soluble HLA-G; SNV, single-nucleotide variant; t-SNE, t-distributed stochastic neighbor embedding; TKI, tyrosine kinase inhibitor; up-LC14A1, upcyte-modified LC14A1; VAF, variant allele frequency; WES, whole-exome sequencing.

Financial support

The German Research Foundation (DFG) funded the study with a grant to JK (KA 5390/2-1) and to MD (CRC 1700; 530990199). All funding sources supporting the work are acknowledged, and authors have nothing to disclose.

Conflicts of interest

The authors have no conflicts of interest to declare. All co-authors have seen and agree with the contents of the manuscript.

Please refer to the accompanying ICMJE disclosure forms for further details.

Authors' contributions

JK initiated and supervised the research study; JK, LS, SK, PVL, and MD designed the experiments; AH and KS supervised the collection of human samples; JK, LS, GM, NH, MV, SS, and TV conducted experiments and acquired data; JK, LS, SS, PVL, WD analyzed data; JK, WD, JHK, SK, SL, and MD wrote the manuscript. All authors had accessed to the study data, discussed the data, and corrected the manuscript. All authors reviewed and approved the final manuscript.

Data availability

The datasets generated and analyzed during this study, including transcriptomic data and functional assay results, are available from the corresponding author upon reasonable request. The up-LC14A1 cell line can be made available to academic researchers for non-commercial use under a material transfer agreement. Additional methodological details are provided in the supplementary materials, and custom protocols are available upon request.

Acknowledgements

We thank Tobias Gosau and Ursula Mueller for their excellent work. We thank Claudia Dettmer, Eileen Maly, and Karina Börner for their excellent technical assistance. We thank Kalpana Dhanuskodi and Githa Ravikumar for processing the WES. We thank Kristoffer Riecken for providing the LeGO vector, which was used to transduce up-LC14A1 cells stably with GFP and LUC. We thank Ulrike Protzer for providing HepG2H1.3 cells for the study.

Supplementary data

Supplementary data to this article can be found online at https://doi.org/10.1016/j.jhepr.2026.101891.

References

Author names in bold designate shared co-first authorship

[1] European Association for the Study of the Liver. EASL Clinical Practice Guidelines on the management of hepatocellular carcinoma. J Hepatol 2025;82:315–374.
[2] Zarlashat Y, Abbas S, Ghaffar A. Hepatocellular carcinoma: beyond the border of advanced stage therapy. Cancers 2024;16:2034.
[3] Desert R, Gianonne F, Saviano A, et al. Improving immunotherapy for the treatment of hepatocellular carcinoma: learning from patients and preclinical models. Gut Liver 2025;2:8.
[4] Llovet JM, Ricci S, Mazzaferro V, et al. Sorafenib in advanced hepatocellular carcinoma. N Engl J Med 2008;359:378–390.
[5] Singal AG, Kanwal F, Llovet JM. Global trends in hepatocellular carcinoma epidemiology: implications for screening, prevention and therapy. Nat Rev Clin Oncol 2023;20:864–884.
[6] Amiot L, Vu N, Samson M. Biology of the immunomodulatory molecule HLA-G in human liver diseases. J Hepatol 2015;62:1430–1437.
[7] **Wang S**, **Wang J**, Xia Y, et al. Harnessing the potential of HLA-G in cancer therapy: advances, challenges, and prospects. J Transl Med 2024;22:130.
[8] Kluckova K, Durmanova V, Bucova M. Soluble HLA-G, its diagnostic and prognostic value and potential target molecule for future therapy in cancer. Bratisl Lek Listy 2021;122:60–617.
[9] Krijgsman D, Roelands J, Hendrickx W, et al. HLA-G: a new immune checkpoint in cancer? Int J Mol Sci 2020;21:4528.
[10] Bian X, Si Y, Zhang M, et al. Down-expression of miR-152 lead to impaired anti-tumor effect of NK via upregulation of HLA-G. Tumour Biol 2016;37:3749–3756.
[11] Okumura T, Joshita S, Yamazaki T, et al. HLA-G susceptibility to hepatitis B infection and related hepatocellular carcinoma in the Japanese population. Hum Immunol 2023;84:401–407.
[12] The human protein Atlas. https://www.proteinatlas.org/. [Accessed 18 May 2026].
[13] Kah J, Staffeldt L, Volz T, et al. Classification of the LC4 primarily-like cell line—recapitulating a CDK4 overexpressing immune evasive HIV-HCV-induced HCC. Viruses 2025;17:653.
[14] Staffeldt L, Mattert G, Riecken K, et al. Generating patient-derived HCC cell lines suitable for predictive in vitro and in vivo drug screening by orthotopic transplantation. Cells 2023;13:82.
[15] Herzog N, Hansen M, Miethbauer S, et al. Primary-like human hepatocytes genetically engineered to obtain proliferation competence display hepatic differentiation characteristics in monolayer and organotypical spheroid cultures. Cell Biol Int 2016;40:341–353.
[16] **Burkard A**, **Dähn C**, Heinz S, et al. Generation of proliferating human hepatocytes using Upcyte® technology: characterisation and applications in induction and cytotoxicity assays. Xenobiotica 2012;42:939–956.
[17] **Wuestenberg A**, **Kah J**, Singethan K, et al. Matrix conditions and KLF2-dependent induction of heme oxygenase-1 modulate inhibition of HCV replication by fluvastatin. PLoS One 2014;9:e96533.
[18] Hösel M, Quasdorff M, Ringelhan M, et al. Hepatitis B virus activates signal transducer and activator of transcription 3 supporting hepatocyte survival and virus replication. Cell Mol Gastroenterol Hepatol 2017;4:339–363.
[19] Allweiss L, Volz T, Lutgehetmann M, et al. Immune cell responses are not required to induce substantial hepatitis B virus antigen decline during pegylated interferon-alpha administration. J Hepatol 2014;60:500–507.
[20] Coelho AV, Moura RR, Crovella S, et al. HLA-G genetic variants and hepatocellular carcinoma: a meta-analysis. Genet Mol Res 2016;15. gmr.15038263.
[21] Adolf IC, Almars A, Dharsee N, et al. HLA-G and single nucleotide polymorphism (SNP) associations with cancer in African populations: implications in personal medicine. Genes Dis 2022;9:1220–1233.
[22] Castelli EC, Mendes-Junior CT, Deghaide NH, et al. The genetic structure of 3'untranslated region of the HLA-G gene: polymorphisms and haplotypes. Genes Immun 2010;11:134–141.
[23] Amodio G, Gregori S. HLA-G genotype/expression/disease association studies: success, hurdles, and perspectives. Front Immunol 2020;11:1178.
[24] Yie SM, Yang H, Ye SR, et al. Expression of human leukocyte antigen G (HLA-G) correlates with poor prognosis in gastric carcinoma. Ann Surg Oncol 2007;14:2721–2729.
[25] Tang Z, Kang B, Li C, et al. GEPIA2: an enhanced web server for large-scale expression profiling and interactive analysis. Nucleic Acids Res 2019;47:W556–W560.
[26] Ringelhan M, Schuehle S, van de Klundert M, et al. HBV-related HCC development in mice is STAT3 dependent and indicates an oncogenic effect of HBx. JHEP Rep 2024;6:101128.
[27] Castelli EC, Veiga-Castelli LC, Yaghi L, et al. Transcriptional and post-transcriptional regulations of the HLA-G gene. J Immunol Res 2014;2014:734068.
[28] Zhang J, Xu M. Apoptotic DNA fragmentation and tissue homeostasis. Trends Cell Biol 2002;12:84–89.
[29] Tsui WHA, Jiang P, Lo YMD. Cell-free DNA fragmentomics in cancer. Cancer Cell 2025;43:1792–1814.
[30] Abdelhamed W, El-Kassas M. Hepatitis B virus as a risk factor for hepatocellular carcinoma: there is still much work to do. Liver Res 2024;8:83–90.
[31] Liu L, Wang L, Zhao L, et al. The role of HLA-G in tumor escape: manipulating the phenotype and function of immune cells. Front Oncol 2020;10:597468.
[32] Mocci S, Perra A, Littera R, et al. Human leukocyte antigen-G in hepatocellular carcinoma driven by chronic viral hepatitis or steatotic liver disease. Sci Rep 2025;15:13331.
[33] **Park Y**, **Park Y**, Lim HS, et al. Soluble human leukocyte antigen-G expression in hepatitis B virus infection and hepatocellular carcinoma. Tissue Antigens 2012;79:97–103.
[34] Han Q, Li N, Zhu Q, et al. Association of serum soluble human leukocyte antigen-G levels with chronic hepatitis B virus infection. Clin Exp Med 2014;14:35–43.
[35] Jasinski-Bergner S, Schmiedel D, Mandelboim O, et al. Role of HLA-G in viral infections. Front Immunol 2022;13:826074.
[36] Donadi EA, Castelli EC, Arnaiz-Villena A, et al. Implications of the polymorphism of HLA-G on its function, regulation, evolution and disease association. Cell Mol Life Sci 2011;68:369–395.
[37] **Allard D**, **Cousineau I**, Ma EH, et al. The CD73 immune checkpoint promotes tumor cell metabolic fitness. eLife 2023;12:e84508.
[38] Kim J, Park S, Kim J, et al. Trogocytosis-mediated immune evasion in the tumor microenvironment. Exp Mol Med 2025;57:1–12.
[39] Caumartin J, Favier B, Daouya M, et al. Trogocytosis-based generation of suppressive NK cells. EMBO J 2007;26:1423–1433.
[40] LeMaoult J, Caumartin J, Daouya M, et al. Immune regulation by pretenders: cell-to-cell transfers of HLA-G make effector T cells act as regulatory cells. Blood 2007;109:2040–2048.
[41] Lin X, Kang K, Chen P, et al. Regulatory mechanisms of PD-1/PD-L1 in cancers. Mol Cancer 2024;23:108.
[42] Własiuk P, Putowski M, Giannopoulos K. PD1/PD1L pathway, HLA-G and T regulatory cells as new markers of immunosuppression in cancers. Postepy Hig Med Dosw 2016;70:1044–1058.
[43] Garcia-Diaz A, Shin DS, Moreno BH, et al. Interferon receptor signaling pathways regulating PD-L1 and PD-L2 expression. Cell Rep 2017;19:1189–1201.
[44] Beers C, Ilaria RL, Luke JJ. Unlocking the promise of innate biology through the HLA-G/ILT2/ILT4 pathway. J Immunother Cancer 2026;14:e013313.

Keywords: Viral hepatocarcinogenesis; Tumor–immune interaction; Immune tolerance; Patient-derived cell line; Orthotopic xenograft; Translational cancer model.
Received 19 August 2025; received in revised form 27 April 2026; accepted 29 April 2026; Available online 8 May 2026

Journal of Hepatology, Volume *8*

Supplemental information

Targeting a tolerogenic HLA-G genotype to tackle immune evasion and adaptive resistance in HBV-driven HCC

Janine Kah, Lisa Staffeldt, Svenja Stefanski, Natalie Herzog, Gregor Mattert, Tassilo Volz, Maximilian Voß, Kornelius Schulze, Asmus Heumann, Werner Dammermann, Sarah Kammerer, Jan-Heiner Küpper, Pablo Villavicencio-Lorini, Maura Dandri, and Stefan Lüth

Targeting a tolerogenic HLA-G genotype to tackle immune evasion and adaptive resistance in HBV-driven HCC

Janine Kah, Lisa Staffeldt, Svenja Stefanski, Natalie Herzog, Gregor Mattert, Tassilo Volz, Maximilian Voß, Kornelius Schulze, Asmus Heumann, Werner Dammermann, Sarah Kammerer, Jan-Heiner Küpper, Pablo Villavicencio-Lorini, Maura Dandri, Stefan Lüth

Table of contents

Supplementary materials and methods……………………………………………………2

Supplementary figures……………………………………………………………………..13

Supplementary tables………………………………………………………………………23

Supplementary references…………………………………………………………………27

1) Supplementary methods

Sample collection, processing and ethics. Human tissue and blood samples were collected, isolated, preserved, and ethically approved as described previously (1, 2). Clinical data was provided in **table S1**. HLA-A typing was performed for patient- and healthy donor-derived tissue and blood cells as described previously (1). The studies were approved by the Ethical Review Committee of the Ärztekammer Hamburg (PV-3578). Handling of human material was performed in accordance with national guidelines and the 1975 Declaration of Helsinki. All animal experiments were conducted in accordance with the European Communities Council Directive (86/EEC) and were approved by the City of Hamburg, Germany (N056/2020). Generation, breeding, and housing of the mice, as well as surgery, were conducted as described previously (2).

up-LC14 generation and long-term storage. Patient-derived HCC cells were isolated as described previously (2) and used to generate proliferation-competent cultures. Cells were genetically modified to obtain a prolonged lifespan as described previously for primary hepatocytes (3, 4). Briefly, cells were resuspended in HCC medium consisting of Dulbecco's Modified Eagle Medium (DMEM; Bio & Sell, 90537 Feucht, Germany) supplemented with 10% fetal calf serum (FCS; Bio & Sell, 90537 Feucht, Germany), 2 mM L-glutamine (Bio & Sell, Germany), and 1% Pen/Strep (Biowest, 49340 Nuaillé, France), centrifuged at 60 × g for 5 min, and resuspended in fresh HCC medium. Per well, 0.24 × 10^6 cells were seeded in collagen-coated 24-well plates (Sarstedt AG & Co. KG, 51588 Nümbrecht, Germany). The following day, cells were transduced with viral particles containing proliferation-inducing genes in HCC medium without Pen/Strep. Recombinant lentiviruses were generated using the ViraPower™ Lentiviral Expression System (Thermo

Fisher Scientific, Waltham, MA 02451, USA) according to the manufacturer's protocol. Virus-containing supernatants were concentrated 20-fold using a filtration system (Vivaspin® 20; Sartorius) and applied directly for infection. Medium was changed every 2–3 days. After ~6 weeks, proliferating liver cancer cells were transferred into 6-well plates (Sarstedt AG & Co. KG, 51588 Nümbrecht, Germany) for expansion in optimized HCC medium consisting of DMEM/Ham's F12 (Bio & Sell, 90537 Feucht, Germany; 1:1) supplemented with 10% FCS, 2 mM L-glutamine, and 1% Pen/Strep. For cryopreservation, ≥1 × 10^6 cells/mL were frozen in 70% DMEM/Ham's F12 (Bio & Sell, 90537 Feucht, Germany), 20% FCS, and 10% DMSO (Sigma, St. Louis, MO 63118, USA) using a Corning™ CoolCell™ freezing container (Thermo Fisher Scientific, Waltham, MA 02451, USA) prior to storage in liquid nitrogen.

HCC cell cultivation, genetic modification and spheroid formation. After thawing, up-LC14 cells were passaged upon reaching 70–80% confluence in Advanced DMEM/F-12 containing L-glutamine and glucose supplemented with 1% P/S and 10% Gibco fetal bovine serum (FBS; all from Thermo Fisher Scientific, Waltham, MA 02451, USA). Stable integration of the LeGO-iG2-Puro+-Luc2 construct by lentiviral transduction was performed as described previously (2). Immortalized HUH-7, Hep3B, and HepG2-H1.3 cells were maintained as described previously (5) in DMEM containing L-glutamine and glucose supplemented with 1% P/S and 10% Gibco FBS (Thermo Fisher Scientific, Waltham, MA 02451, USA). Cells were detached using Detachin™ Cell Detachment Solution (AMSBIO, Abingdon, UK) and cultured at 37 °C and 5% CO_2.

For 2D experiments, HCC and up-LC14 cell lines were seeded in 96- or 24-well plates at 10,000 or 50,000 cells/well using the corresponding passage medium and allowed to

adhere for 24 h prior to treatment. For 3D spheroid formation, 10,000–30,000 cells/well of luciferase-transduced up-LC14 cells were seeded on BIOFLOAT™ cell culture plates in Advanced DMEM/F-12 containing L-glutamine and glucose supplemented with 1% P/S and 10% Gibco FBS (Thermo Fisher Scientific, Waltham, MA 02451, USA) and maintained for 3 weeks to follow spheroid formation. The LeGO-iG2-Puro+-Luc2 construct (3rd generation HIV1-derived self-inactivating vector) enabled visualization. During spheroid formation, images were acquired automatically using the BX-780 microscope (Keyence, Osaka, 533-0033 Japan). HLA-G knock-down was performed using the human pre-designed siRNA Sets (15062) directed against the Gene ID: 3135 (cat#: HY-RS06217, MCE, NJ 08852, USA) following the manufacturer´s instructions.

NK92 culture and *in-vitro* treatment. For *in-vitro* experiments, enriched NK92 cells were thawed and cultured in MEM α medium (Gibco, Thermo Fisher, Massachusetts 02142, USA) supplemented with 12.5% horse serum (Gibco, Thermo Fisher, Massachusetts 02142, USA), 12.5% fetal bovine serum (Thermo Fisher Scientific, Massachusetts 02142, USA), 500 IU/mL rhIL-2 (Sartorius, Göttingen, Germany) and human IL-2 IS (Miltenyi Biotec, Bergisch Gladbach, Germany). After sufficient expansion, NK92 cells were counted, washed by centrifugation at 350 g, and transferred to target cells at effector-to-target ratios of 1:1, 1:5, and 1:10. Ratios have been selected based on preliminary titration experiments to ensure robust and reproducible detection of NK92 cell–mediated killing. Co-cultures were maintained for 72 hours to capture cumulative cytotoxic effects and functional adaptation of NK92 cells beyond the initial contact phase. Shorter incubation periods (e.g., 24 hours) primarily reflect early activation and transient killing events, whereas 72 hours enables assessment of sustained effector activity. At indicated time points, supernatants and/or NK92 cells were collected for downstream analyses. Viability

was monitored by xCELLigence analysis (see “Doubling time assay”). Supernatants were used for cfDNA isolation. NK92 cells and target cells were analyzed using TaqMan single assays (**table S2**) following oligonucleotide isolation or by flow cytometry (**table S3**).

Doubling time assay (xCELLigence). Cell doubling was determined using the xCELLigence RTCA SP device (ACEA Biosciences, 92121 San Diego, USA). up-LC14 cells were seeded at 10,000 cells/well in an E-Plate 16 (ACEA Biosciences) and cultured for 72 h in up-LC14 culture medium. Doubling time was calculated from n = 5 technical replicates using xCELLigence RTCA Software v2.0 during the exponential growth phase.

Isolation of oligonucleotides. For RNA isolation from cultured cells, HCC cell lines were seeded into 24-well plates at 50,000 cells/well and harvested at ≥80% confluence. For RNA isolation after immune cell transfer, HCC cell lines were seeded as described above and treated with the indicated immune cells for 24 h; four wells per condition were pooled in RNA lysis buffer and stored at −80 °C until extraction. For isolation of RNA and DNA from chimeric or human liver samples, representative tumorigenic tissue was selected and 10–30 mg processed as described previously (1). RNA was isolated using the RNeasy Micro™ (cell lines) and RNeasy Mini™ (tissue) RNA purification kits (Qiagen, 40724 Hilden, Germany) according to the manufacturer’s instructions (6).

TaqMan-based gene expression analysis. Gene expression was quantified by two-step PCR. cDNA synthesis was performed using the MMLV Reverse Transcriptase™ 1st-Strand cDNA Synthesis Kit (Lucigen, Middleton, Wisconsin 53562, USA) according to the manufacturer’s instructions. Human-specific primer/probe sets from the TaqMan Gene Expression Assay System (Life Technologies, 92008 Carlsbad, California, USA; Table S1) were used (**table S2**). Samples were analyzed on the QuantStudio 7™ Real-Time PCR

System (Life Technologies, 92008 Carlsbad, California, USA) using the Fast protocol for 96-well plates (95 °C for 20 s; 40 cycles of 95 °C for 15 s and 60 °C for 20 s; cooling at 20 °C for 2 min). TaqMan Fast Advanced Master Mix (5×) (Life Technologies, 92008 Carlsbad, California, USA) was used according to the manufacturer's instructions. Data were analyzed using Design & Analysis Software v2.8.0 (Thermo Fisher Scientific, Massachusetts, USA). Gene expression was normalized to human housekeeping genes ribosomal protein L0 (RPL0), RPL30, and GAPDH.

Whole-exome sequencing and variant analysis. Genomic DNA was isolated from spatially separated tumor regions as described previously (6). DNA quantity and integrity were assessed by Qubit fluorometric quantification and fragment analysis (Agilent TapeStation). For each sample, 50 ng genomic DNA was used for whole-exome library preparation with hybrid-capture enrichment (Twist Precision Exome DX kit), followed by amplification and quality control. Paired-end sequencing (2 × 150 bp) was performed on an Illumina platform to obtain a mean on-target coverage ≥100fold. Demultiplexing was performed using Illumina software, and data were exported in bcl2/fastq format.

Secondary analysis was performed using IVD-CE Varvis® software v2.7.3, including adapter trimming and quality filtering prior to alignment to GRCh38/hg38. Alignments were sorted, duplicates marked, and base quality recalibration and indel realignment performed; base calling reached Q30 > 90%. SNVs and small indels were identified using a somatic variant-calling workflow. Variant call files were filtered for quality parameters (including read depth, mapping quality, and strand bias), retaining variants with ≥10 supporting reads and AltAF ≥0.05. Analyses were restricted to coding and flanking intronic regions, 5′/3′UTRs within the captured exome, and spiked-in mitochondrial regions.

Tertiary analysis was performed to predict HIGH or MODERATE functional impact. Varvis® output included genomic coordinates, quality/coverage metrics, functional annotations, and database annotations (dbSNP, ClinVar, OMIM). Variant classification followed ACMG/AMP guidelines.

Downstream analyses were performed in R (v4.x) using RStudio. VCF files were imported and converted to tabular format for filtering and annotation-based analyses. Variants were filtered to retain putative functional events (non-synonymous, predicted HIGH or MODERATE impact, VAF ≥0.05, ≥10 supporting reads). Gene-level variant counts were calculated for tumor center and margin, and top variant-enriched genes were identified by total variant number per gene. Visualizations were generated using ggplot2, dplyr, and tidyr. VAF distributions were plotted as density plots. Variant overlaps were visualized using Venn diagrams (VennDiagram or ggVennDiagram). Annotation-based summaries (dbSNP, ClinVar) were calculated from annotated tables and visualized as stacked bar charts. Locus-specific variant distributions for immune-relevant genes were extracted and plotted in ggplot2.

RNA sequencing and differential gene expression analysis. Total RNA was extracted from primary hepatocytes, the up-LC14A1 primary-like cell line, and immortalized HCC lines (HepG2H1.3, Huh7, Hep3B) as described previously (1). RNA quality and quantity were assessed using a NanoDrop spectrophotometer (Thermo Fisher Scientific, 76185 Karlsruhe, Germany) and an Agilent Bioanalyzer 2100 (Agilent Technologies, 76337 Waldbronn, Germany) with High Sensitivity DNA Chips (Cat. 5067-4626). Samples with RIN > 7.0 were used. Libraries were prepared using the TruSeq Stranded mRNA Kit (Illumina, 81669 Munich, Germany) according to the manufacturer’s instructions (mRNA

enrichment, fragmentation, cDNA synthesis, adapter ligation, PCR amplification). Libraries were quantified using a Qubit fluorometer (Thermo Fisher Scientific, 76185 Karlsruhe, Germany) with the Qubit 1X dsDNA HS Assay Kit (Q33230) and assessed for size distribution using the Agilent Bioanalyzer 2100 (Agilent Technologies, 76337 Waldbronn, Germany). Sequencing was performed on an Illumina NextSeq 1000/2000 platform generating 200 bp paired-end reads. Technical replicates ($n = 2$) were included per sample. Reads were trimmed using Trimmomatic, aligned to GRCh38 using STAR, and quantified using featureCounts. Differential gene expression was analyzed using DESeq2; significance thresholds were adjusted $p < 0.05$ and $|log2FC| > 2$. DEGs were visualized using volcano plots, z-score-based hierarchical heatmaps, and match z-score plots using IPA, GraphPad, and R-based tools [19–21]. For t-SNE visualization, raw count matrices of up-LC14A1, HepG2-H1.3, Hep3B, and HUH-7 were processed in RStudio using DESeq2. Counts were variance-stabilized (VST), mapped from Ensembl IDs to gene symbols, and summarized at gene level. Canonical pathway genes were obtained from the MSigDB C2:CP collection, while toxicity- and stress-related genes were selected from MSigDB via keyword filtering (xenobiotic response, oxidative stress, hypoxia, DNA damage, inflammatory signaling). Expression matrices of selected pathway genes were z-score normalized per gene and subjected to t-SNE using the Rtsne package (perplexity = 15). A shared embedding was generated at gene level, and cell line–specific pathway activity was visualized by calculating gene-wise expression differences for each cell line versus the remaining lines, z-transforming these values, and overlaying them onto the shared t-SNE coordinates. Analyses and visualizations were performed in R using DESeq2, Rtsne, msigdbr, and ggplot2.

Immunofluorescence staining. For *in-vitro* histological characterization of up-LC14 cells, 10,000 cells were seeded into 96-well plates and fixed at ~80% confluence with 4% PFA for 10 min after washing with 1× PBS. Cryosections (5 µm) were prepared from mouse-derived tissues representing tumorigenic and non-tumorigenic areas, stored at −80 °C, and fixed with 4% PFA for 10 min prior to staining. After fixation, mouse tissue sections and up-LC14 cells were stained by immunofluorescence using primary antibodies (**table S5**) with overnight incubation. Signals were detected using Alexa 488- (green) or Alexa 555- (red) labeled secondary antibodies (Invitrogen, Darmstadt, Germany) after 1 h incubation. Nuclei were stained with Hoechst 33258 (Invitrogen, Eugene, OR 97402, USA) and mounting was performed using mounting medium (Dako Deutschland GmbH, 22769 Hamburg, Germany). Imaging was performed using fluorescence microscopy (BZ-9000 and BX-780, Keyence, Osaka, 533-0033 Japan). Tissue images were acquired manually using consistent exposure times for comparable material/antibody settings. up-LC14 cell images were acquired automatically using identical magnification and exposure for the same antibodies. Bright-field images were acquired at matching magnification to phase contrast.

Flow cytometry. Flow cytometry analyses of human- and mouse-derived HCC cells were performed as previously described (2); antibodies are listed in **table S4**. NK92 cells were analyzed at indicated time points after treatment using a SymphonyA3™ flow cytometer (Becton Dickinson, New Jersey 07417, USA) and stained using antibodies listed in Table S2. Antibodies were incubated for 30 min at room temperature protected from light. Prior to staining, cells were incubated with Human BD Fc Block™ (Becton Dickinson, New Jersey 07417, USA). Gates were defined using fluorescence minus one (FMO) controls.

ELISA-based protein measurements. Human sHLA-G levels in human serum and cell culture supernatants were measured using the Human Major Histocompatibility Complex Class I G ELISA Kit (Thermo Fisher, Waltham, MA 02451, USA) according to the manufacturer's instructions; samples were diluted 1:1–1:3. Human alpha-1 antitrypsin (AAT) levels in murine serum were quantified using the Human alpha 1 Anti-trypsin ELISA Kit (abcam, Cambridge, MA 02139, USA) according to the manufacturer's instructions; murine serum was diluted 1:2500. Human perforin levels in cell culture supernatants were measured using ELISA Flex: Human Perforin (HRP) (Mabtech AB, Nacka Strand, 13152, Sweden) according to the manufacturer's instructions. Assay Diluent (5×; Cat. 421203) and TMB Substrate Set (Cat. 421101) were purchased from BioLegend. Samples were diluted 1:100.

HBV DNA quantification. HBV DNA was extracted from serum and cell culture supernatants using the QIAamp MinElute™ Virus Spin Kit (Qiagen, Hilden, Germany). Quantification was performed by TaqMan PCR using an HBV-specific probe (**table S2**). Cloned HBV DNA references were amplified in parallel to generate a standard curve.

cfDNA isolation and fragment analysis. For cfDNA isolation, cell culture supernatants were centrifuged for 10 min at 1000 x g and stored at −20 °C. cfDNA was isolated using the MagMAX™ cfDNA Isolation Kit (Thermo Fisher, Waltham, MA 02451, USA) according to the manufacturer's instructions. Samples were diluted 1:2 with water before denaturation with protein kinase K and sodium dodecyl sulphate (SDS) for 20 min at 60 °C. Samples were incubated with MagMAX™ Cell Free DNA Lysis/Binding Solution and MagMAX™ Cell Free DNA Magnetic Beads for 20 min, chilled on ice for 5 min, and cfDNA was bead-bound. Bound fragments were washed with MagMAX™ Cell Free DNA Wash

Solution and 80% ethanol. After centrifugation, supernatants were removed and cfDNA was eluted using MagMAX™ Cell Free DNA Elution Solution into fresh microcentrifuge tubes. For ALU-based fragment analysis, cfDNA qPCR reactions were performed in 14 µl containing 1 µl template, 7.5 µl PowerUp™ SYBR™ Green Master Mix (Thermo Fisher, Waltham, MA 02451, USA), 4.5 µl RNase-free water, and 1 µl primer mix (**table S6**). Short and long ALU fragments (ALU-115 and ALU-247 (7)) were amplified using the QuantStudio™ 7 Pro Real-Time PCR System (Thermo Fisher Scientific, Massachusetts, USA) with cycling conditions of 95 °C for 2 min, followed by 40 cycles of 95 °C for 15 s and 60 °C for 1 min, and cooling at 20 °C for 2 min. Data were analyzed using Design & Analysis Software v2.8.0 (Thermo Fisher Scientific, Massachusetts, USA). For locus-specific analysis of HLA-G, the TaqMan HLA-G assay Hs03045108_m1 (amplicon ~109 bp; designed for transcript detection across the exon 5–6 boundary) was applied using the same workflow.

Statistics and sample size. Graph design and statistical analyses were performed using GraphPad Prism v10 (GraphPad Software, Inc., La Jolla, CA, USA). For cell viability measurements, n = 5 technical replicates were used per group and analyzed by two-way ANOVA. RNA-based DEG and WES analyses included n = 2 technical replicates from HUH-7, Hep3B, HepG2H1.3, up-LC14A1 cell lines, patient material and healthy hepatocytes. HBV DNA titers were measured in n = 3 technical replicates. For the cytometric analysis shown in Fig. 3B, cells from n = 3 chimeric mice were pooled after isolation and measured in n = 2 technical replicates; patient-derived isolated liver cancer cells and cell lines were analyzed accordingly. For AAT endpoint measurement, serum was collected from n = 3 (HUH7), n = 4 (Hep3B), n = 4 (HepG2.1.3), and n = 14 (up-LC14A1) mice. For sHLA-G detection, sample sizes are provided in the corresponding

figure legends. Perforin was measured from n = 2 technical replicates per condition. For cfDNA isolation, n = 4 technical replicates were used and analyzed using one-way ANOVA with multiple comparisons. For gene expression analyses of cell culture samples, n = 3 technical replicates were used. Delta MFI was calculated from n = 2 technical replicates at the indicated time points. For comparisons involving more than two conditions, one-way ANOVA was applied; two-condition comparisons used Student's t-test. Statistical outputs are shown where applicable and significance is indicated as *$p < 0.05$; **$p \leq 0.01$; ***$p \leq 0.001$; ****$p \leq 0.0001$.

2) Supplementary figures

Figure S1

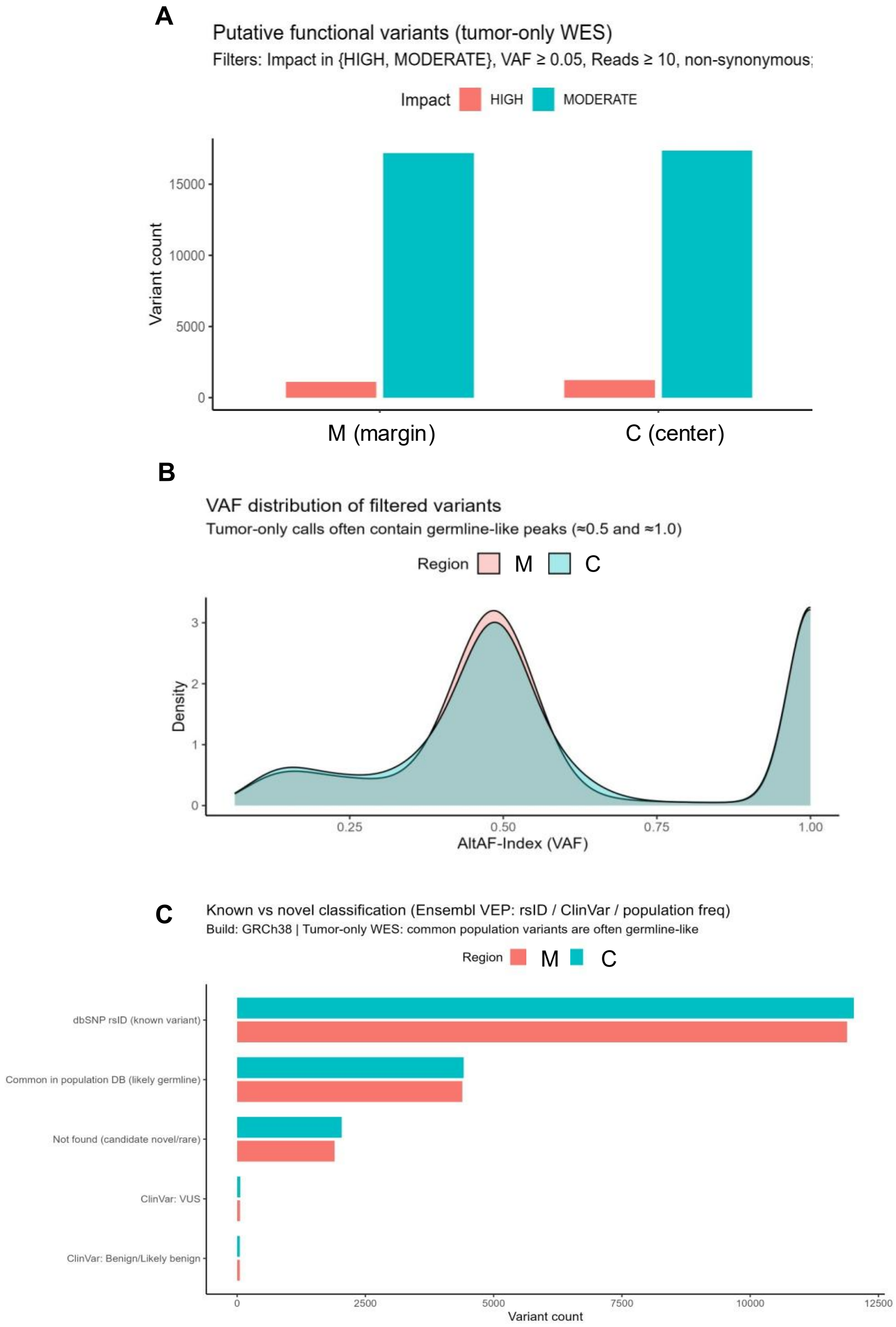

Fig. S1. Summary of patients WES-based variant characteristics. (**A**) Number of putative functional variants detected by whole-exome sequencing. Variants were filtered for predicted impact (HIGH or MODERATE), variant allele frequency (VAF) ≥0.05, read

depth ≥10, and non-synonymous annotation. Bar charts show total number of variants identified in tumor margin (M) and tumor center (C), stratified by predicted impact. (**B**) Distribution of variant allele frequencies of filtered variants. Density plots show VAF distributions for tumor margin (M) and tumor center (C). Tumor-only variant calling can include variants with germline-like VAF peaks at approximately 0.5 and 1.0. (**C**) Classification of variants based on annotation and population frequency. Variants were categorized using Ensembl Variant Effect Predictor annotations, dbSNP identifiers, ClinVar annotation, and population frequency databases (GRCh38 build). Bars show counts for tumor margin (M) and tumor center (C) across the indicated categories, including known variants, variants common in population databases, variants not found in reference databases, and ClinVar annotations.

Figure S2

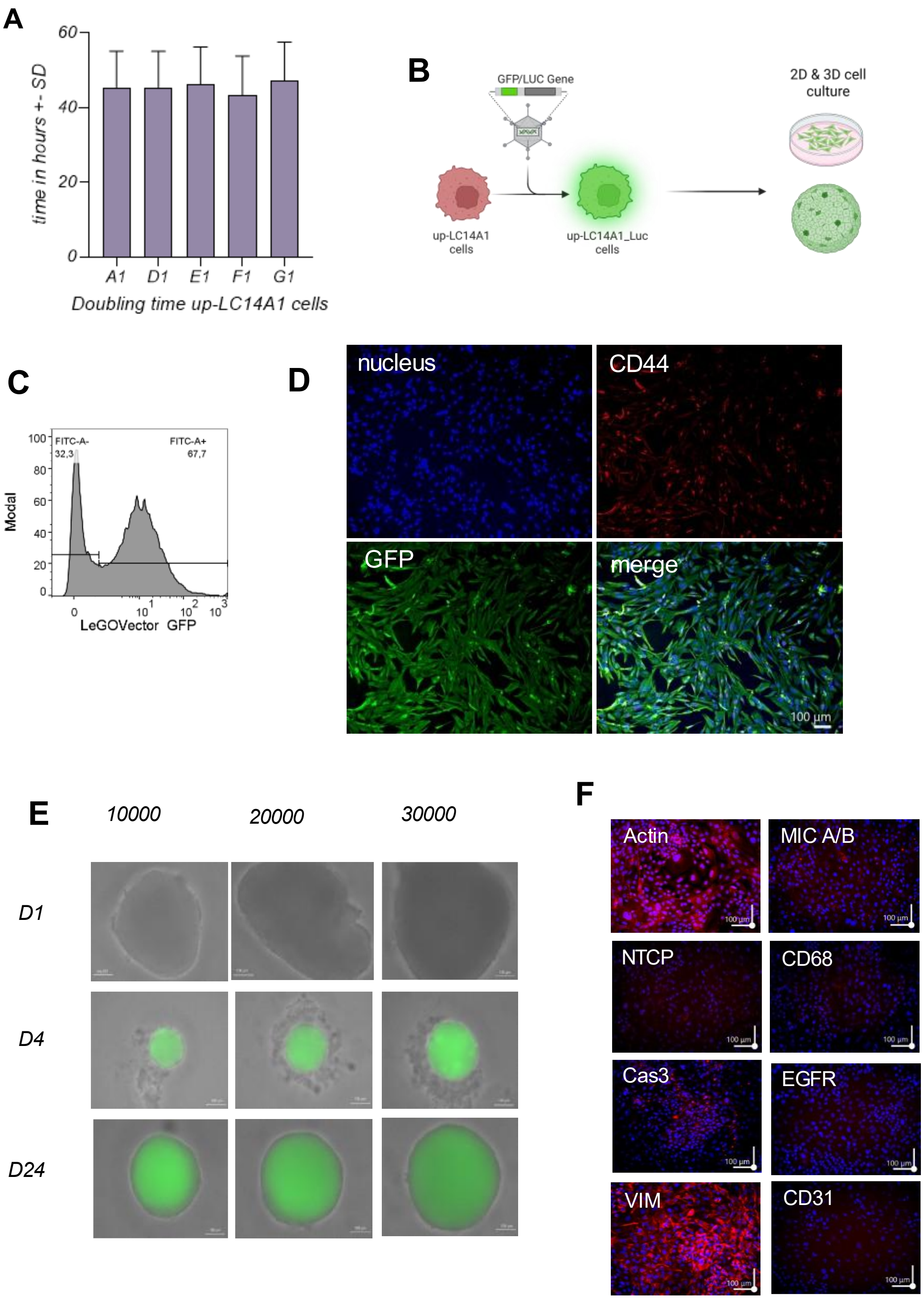

Fig. S2. Phenotypic characteristics, lentiviral modification by LeGoVector, spheroid forming capacities and Protein expression level of up-LC14A1 cells. (A) Impendence measurement based doubling time analysis of up-LC14A1 cell replicates (A1, D1, E1, F1,

G1). Bars represent mean ± SD. (**B**) Illustration of the LeGoVektor-based modification using up-LC14A1 cells to generate up-LC14A1luc cells (MOI10 and 100). Up-LC14A1luc cells were analyzed using flow cytometry (**C**), immunofluorescence staining (**D**), and spheroid forming (**E**). (**C**) Flow cytometric analysis 7days after of LegoVector (GFP/luc2) transduction. Histogram shows over 60% GFP-positive cells. (**D**) Representative fluorescence microscopy of reporter-labeled up-LC14A1 cells in 2D culture showing GFP expression together with CD44 staining and nuclear counterstaining. Scale bars, 100 µm. (**E**) Representative images of spheroid formation by up-LC14A1_luc cells. Spheroids were generated at the indicated seeding densities and imaged at the indicated time points. GFP signal marks reporter-expressing cells. Scale bars, 100µm. (**F**) Representative merged captures of immunofluorescence staining of up-LC14A1 cells grown in 2D culture. Cells were stained for the indicated markers, including structural, epithelial, immune-related, and signaling proteins. Nuclei were stained with Hoechst. Scale bars, 100 µm.

Figure S3

A

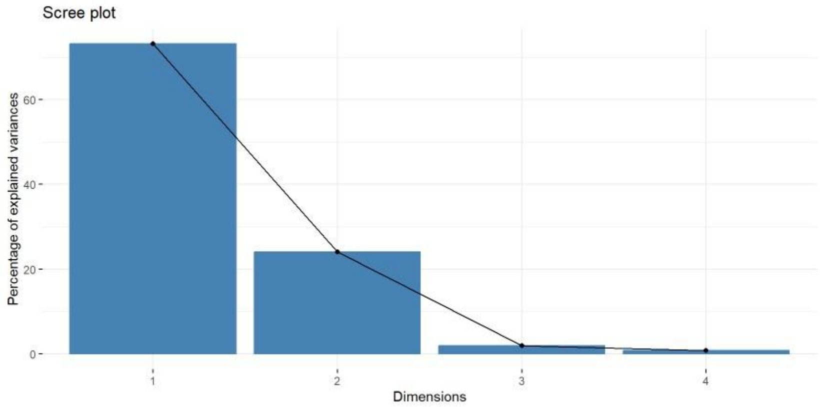

B

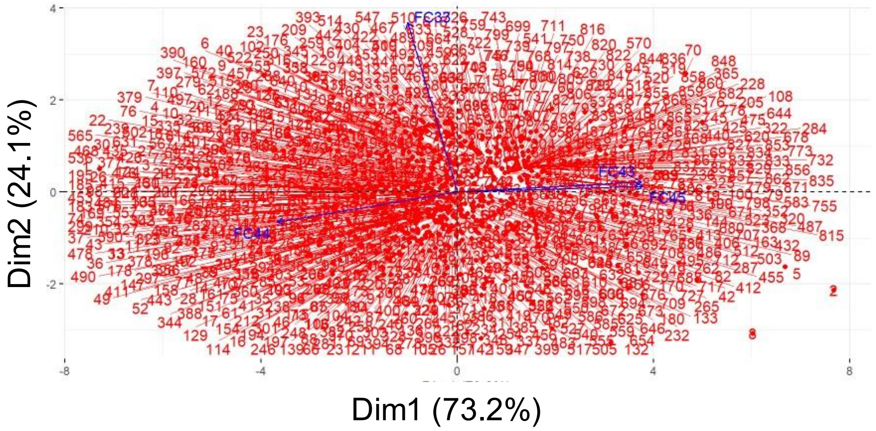

C

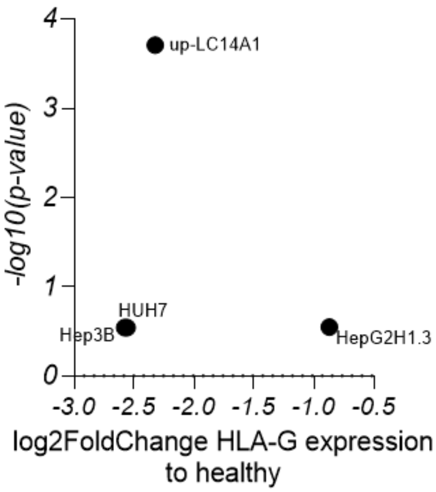

G

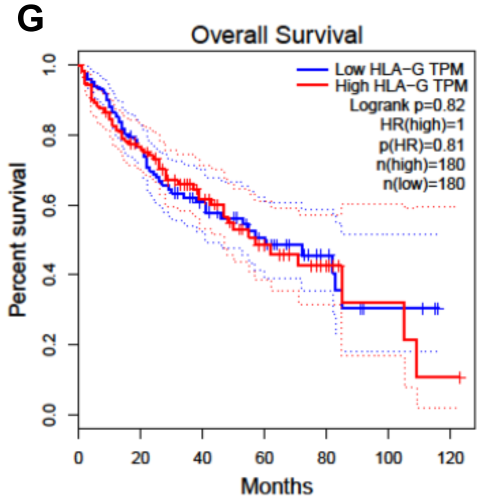

Fig. S3 Principal Component Analysis (PCA) of up-LC14A1 cells and HLA-G expression. (**A**) Scree plot showing the percentage of variance explained by each principal component. The first component (Dim1) explains the majority of the variance (73.2%), followed by Dim2 (24.1%). (**B**) PCA plot of the first two principal components (Dim1 and Dim2) for HCC cell lines. Each point represents an individual sample, with the dimensions Dim1 (73.2%) and Dim2 (24.1%) labeled on the axes. The plot highlights the distribution and clustering of the samples based on their gene expression profiles. FC33 = up-LC14A1 vs PHH; FC43 = Hep3B vs PHH; FC44 = HepG2H1.3 vs PHH; FC45 = HUH7 vs PHH). (**C**) Vulcano plot showing data extracted from DEG data sets of the indicated cell lines for HLA-G. (**D**) Overall survival data, corresponding to HLA-G expression level, extracted from GEPIA (https://gepia3.bioinfoliu.com/genecard/#HLA-G).

Figure S4

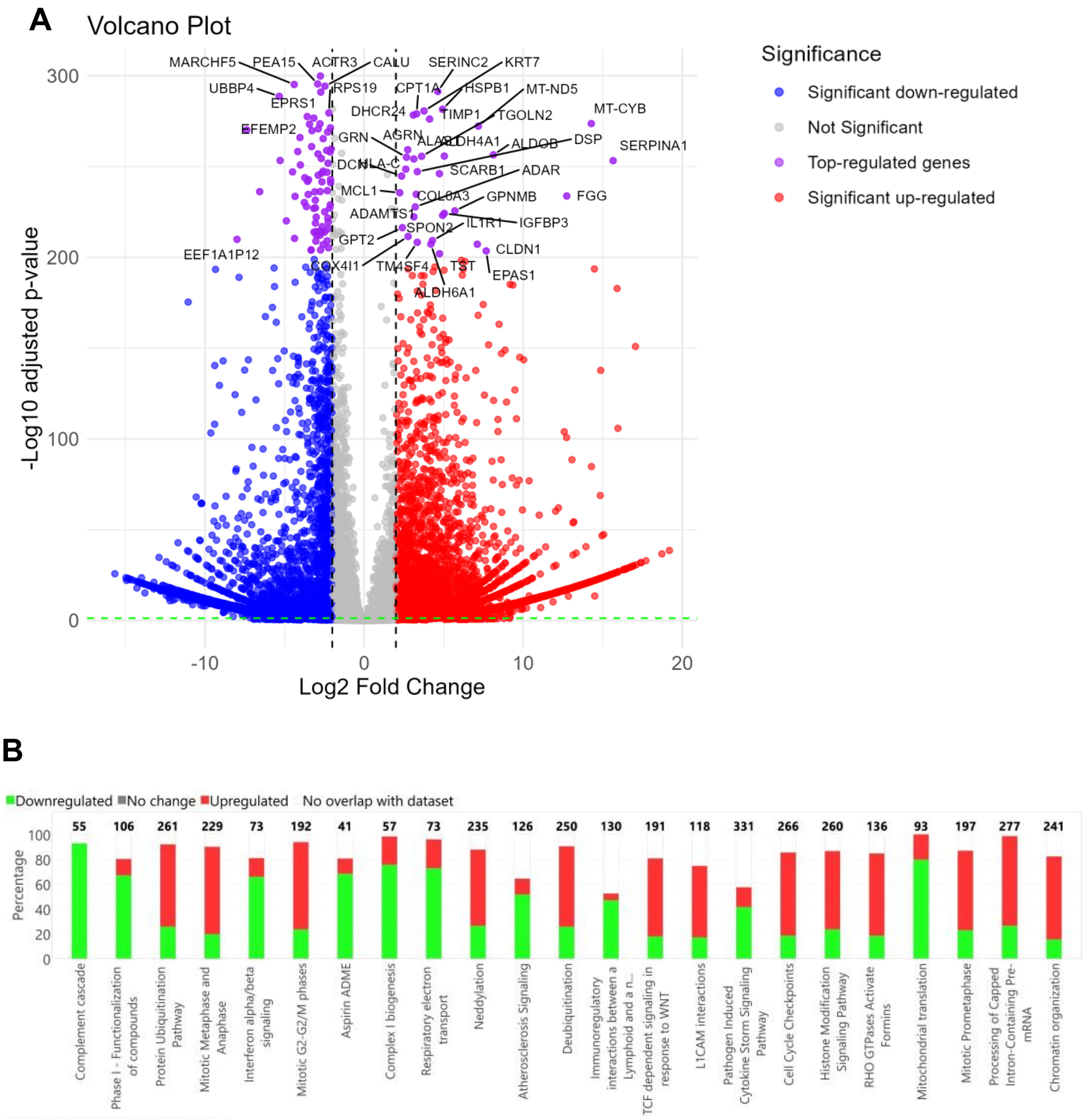

Fig. S4. Differential gene expression and pathway analysis of up-LC14A1 cells compared with primary human hepatocytes. (**A**) Volcano plot of the differential gene expression (DEG) analysis comparing up-LC14A1 cells with primary human hepatocytes (PHH). Genes are plotted according to log2 fold change and −log10 adjusted p value. Significantly upregulated genes are shown in red, significantly downregulated genes in blue, and non-significant genes in grey. Selected highly regulated genes are annotated.
(**B**) Expression profile of genes involved in selected canonical pathways. Bar plots generated using Ingenuity Pathway Analysis (IPA) show the proportion of genes within each pathway that were downregulated (green) or upregulated (red) in up-LC14A1 cells

compared with PHH. The number of genes from the dataset contributing to each pathway is indicated above the bars. PHH, primary human hepatocytes.

Figure S5

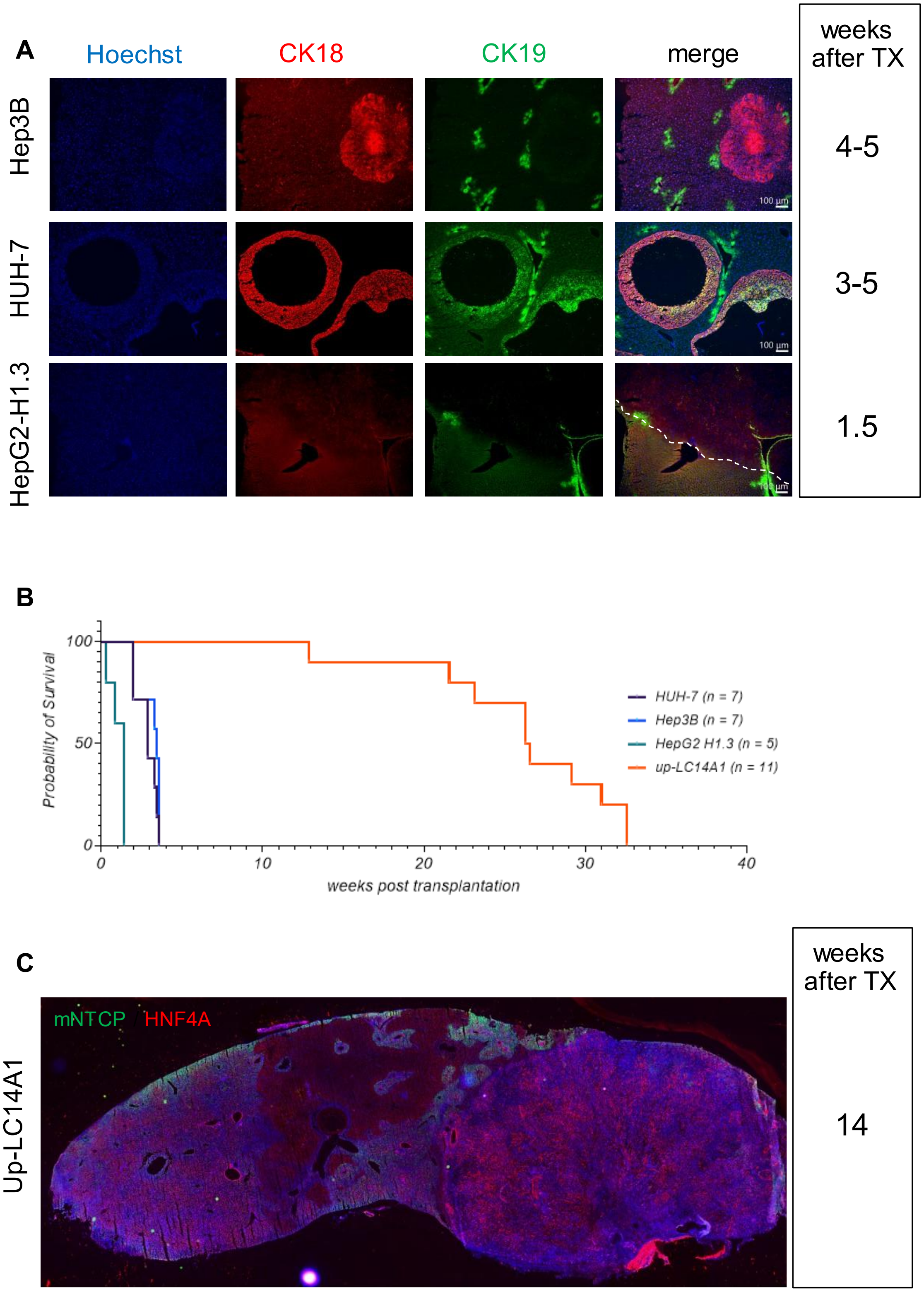

Fig. S5 Comparison of immortalized HCC cell lines and up-LC14A1 cells *in-vivo*
(**A**) Immunofluorescence staining of Hep3B, HUH-7, and HepG2.1.3 cells showing expression of cytokeratin (CK18 in red, CK19 in green). Nuclei were stained with Hoechst (blue). Representative images of epithelial marker expression across different cell lines are shown. Scale bar, 100 µm. (**B**) Kaplan-Meier Survival analysis of xenografted mice using the indicated HCC cell lines. Transplantation was performed with the standardized protocol, as described previously (2). (**C**) Representative immunofluorescence staining of up-LC14A1 xenograft sections showing co-expression of mNTCP (green) and HNF4A (red), indicating hepatocyte-like differentiation in the xenograft. Nuclei were stained with Hoechst (blue). On the right side of the figure, the weeks after transplantation and before scarification of the mice which were used for cryosection-based immunofluorescence staining, are mentioned.

Figure S6

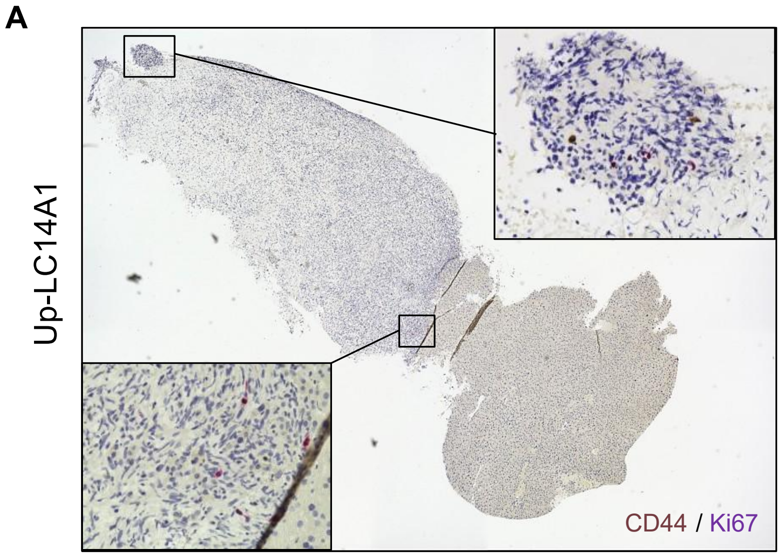

Fig. S6 Characterization up-LC14A1 cells on ki67 and CD44 expression after 12 weeks of progression in-vivo. Immunohistochemistry of up-LC14A1 xenograft sections showing co-expression of CD44 (left inset; brown) and Ki67 (right inset; red). The data demonstrate moderate proliferative activity and stem cell-like features in up-LC14A1 cells in-vivo. Scale bar, 500 µm (main image), 100 µm (insets).

Figure S7

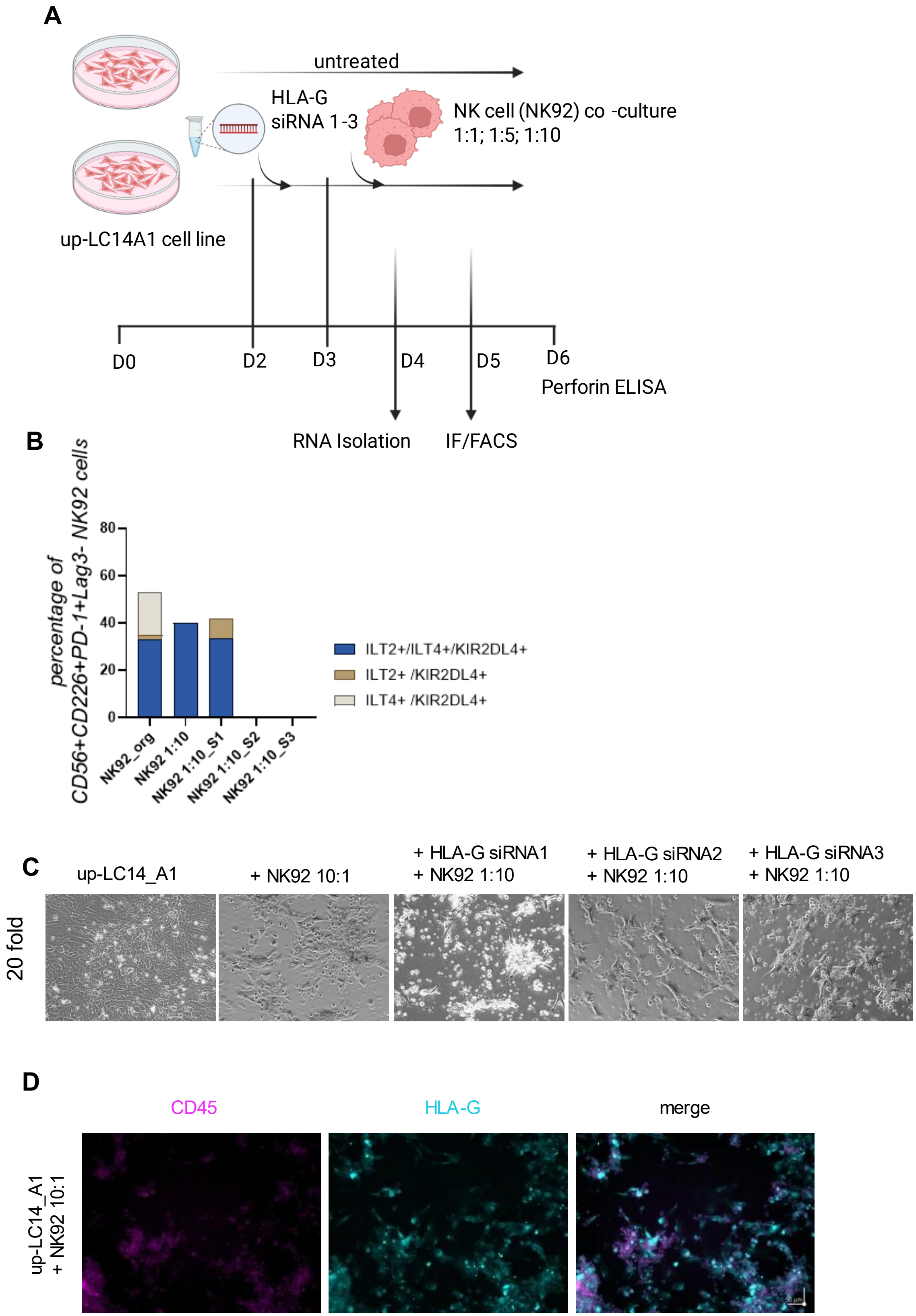

A
untreated
HLA-G
siRNA 1-3
NK cell (NK92) co -culture
1:1; 1:5; 1:10
up-LC14A1 cell line
D0
D2
D3
D4
D5
D6
Perforin ELISA
RNA Isolation
IF/FACS
B
percentage of
CD56+CD226+PD-1+Lag3- NK92 cells
80
60
40
20
0
NK92_org
NK92 1:10
NK92 1:10_S1
NK92 1:10_S2
NK92 1:10_S3
ILT2+/ILT4+/KIR2DL4+
ILT2+ /KIR2DL4+
ILT4+ /KIR2DL4+
C
up-LC14_A1
+ NK92 10:1
+ HLA-G siRNA1
+ NK92 1:10
+ HLA-G siRNA2
+ NK92 1:10
+ HLA-G siRNA3
+ NK92 1:10
20 fold
D
CD45
HLA-G
merge
up-LC14_A1
+ NK92 10:1

Fig. S7 HLA-G siRNA based knockdown experiments, KIR2DL4 expression and microscopy following treatment. (**A**) Visualisation of treatment settings, strategy of sample generation as well as acquisition for gene and protein expression after co-culture conditions. (**B**) Percentage of NK92 subpopulations expressing KIR2DL4 for the indicated treatment conditions. (**C**) 20fold phase contrast bright field cell culture captures, 48 hours after NK92 co-culture corresponding to immunofluorescence staining shown in Fig. 6 and 7. (**D**) Single channel captures of HLA-G and CD45, as well as the merged capture shown in Fig. 7.

3) Supplementary tables

Table S1:

a) N13HCC cohort clinical data

patient number	sex	age	clinical classification	entity	intratumoral HBV RNA/DNA	sHLA-G detection material
2	f	62	DD HCC	Steatohepatitis	NEG	no
3	m	64	HCC G0	ETOH	NEG	no
4	m	70	HCC	viral-induced (HCV-HIV)	NEG	yes
6	f	83	HCC	AIH/PBC	POS	yes
7	m	55	HCC G1	ETOH	NEG	yes
8	m	58	HCC G1	viral-induced (HCV-HBV)	NEG	yes
9	f	69	HCC G1	MASH	POS	yes
10	f	55	HCC	ETOH	NEG	no
11	m	58	HCC G1	viral-induced (HBV)	POS	yes

12	m	69	HCC G1	viral-induced (HBV/HEV)	POS	no
13	m	81	HCC G2	MASH	POS	yes
14	m	31	HCC G3	viral-induced (HBV)	POS	yes
15	m	82	HCC G1	Steatohepatitis	POS	yes

b) early-stage biopsy clinical data

patient number	sex	age in years / month	clinical classification	sHLA-G detection material
124-biopsy	m	65	viral hepatitis	yes
171 – biopsy	f	73	Autoimmune Hepatitis	yes
196 - biopsy	m	51	Liver cirrhosis	yes
193 - biopsy	f	50	Alcoholic fibrosis and sclerosis	yes

Table S2: Taqman assay for real-time PCR

gene name	Assay-ID	dye
RPL0	Hs00420895_gH	FAM-MGB
RPL30	Hs00265497_m1	VIC-MGB
GAPDH	Hs99999905_M1	FAM-MGB
HMGCoAR	Hs00168352_m1	FAM-MGB
HBV S	Pa03453405_s1	FAM-MGB
CD44	Hs01075864_m1	FAM-MGB
HNF4A	Hs00230853_m1	VIC-MGB
STAT3	Hs00374280_m1	VIC-MGB
HLA-G	Hs03045108_m1	VIC-MGB
CLRN3	Hs00153677_m1	FAM-MGB
AADAC	Hs00153677_m1	FAM-MGB

ALB	Hs00609411_m1	VIC-MGB
MIK67	Hs04260396_g1	FAM-MGB
TP53	HS01034249_m1	FAM-MGB
IFNg	Hs00989291_m1	FAM-MGB
NLRP3	Hs00918082_m1	VIC-MGB
CD8	Hs00233520_m1	FAM-MGB
CD45	Hs04189704_m1	FAM-MGB
CD24	Hs02379687_s1	FAM-MGB
Glut-1	Hs00892681_m1	FAM-MGB

Table S3: Antibodies for processed Flow cytometry of NK cells

antigen	fluorochrome	company
CD3	BUV395	BD Horizon
CD14	FITC	BD Pharmingen
CD45	BV480	BD Horizon
CD56	PE	BD Pharmingen
CD16	BV650	BD Horizon
CD19	BV786	BD Horizon
CD62L, L-Selectin	BUV496	BD OptiBuild
CD178, Fas-Ligand	BUV615	BD Optibuild
CD226, DNAM1	BUV737	BD Optibuild
CD335, NKp46	BV510	BD Horizon
CD162, PGSL-1	BV650	BD Optibuild
CD336, NKp44	RB780	BD Horizon
CD337, NKp30	R718	BD Horizon
CD253, TRAIL	BV421	BD Horizon
CD314, NKGD2	PerCP-Cy5.5	BD Pharmingen
CD279, PD-1	BV711	BD OptiBuild
CD11a, LFA-1 a-chain	BUV496	BD Optibuild
CD49d, VLA-4 a-chain, Integrin a	BUV615	BD Horizon
CD107a	BV510	BD Horizon
CD184, CXCR4	BV650	BD OptiBuild
TIGIT	RB780	BD Horizon
CD183, CXCR3	R718	BD Horizon
CD15s	BV421	BD Horizon
CD366, TIM-3	PerCP-Cy5.5	BD Pharmingen
CD197, CCR7	BV711	BD Horizon
ILT2, CD85j	BUV615	BD Optibuild
ILT4, CD85d	BV421	Miltenyi Bioscience
KIR2DL4, CD158d	PE-Cy5	Thermo Fischer
CD223, Lag-3	BV605	BD Horizon

Table S4: Antibodies for processed Flow cytometry for HCC cells- ex-vivo and in-vitro

antigen	fluorochrome	company
CD105	PE-Vio® 770	Miltenyi Biotec B.V. & Co. KG, D
CD13	APC	Miltenyi Biotec B.V. & Co. KG, D
CD133/1	PE-Vio 615	Miltenyi Biotec B.V. & Co. KG, D
CD24	PE-Vio 770	Miltenyi Biotec B.V. & Co. KG, D
CD279 (PD1)	PE	Miltenyi Biotec B.V. & Co. KG, D
CD3	PE-Vio® 770	Miltenyi Biotec B.V. & Co. KG, D
CD326	PE-Vio615	Miltenyi Biotec B.V. & Co. KG, D
CD326	VioBlue	Miltenyi Biotec B.V. & Co. KG, D
CD34	PE-Vio® 770	Miltenyi Biotec B.V. & Co. KG, D
CD366	APC	Miltenyi Biotec B.V. & Co. KG, D
CD44	APC	Miltenyi Biotec B.V. & Co. KG, D
CD45	APC-Vio® 770	Miltenyi Biotec B.V. & Co. KG, D
CD73	PE	Miltenyi Biotec B.V. & Co. KG, D
CD73	PE-Vio® 615	Miltenyi Biotec B.V. & Co. KG, D
CD90	VioBlue	Miltenyi Biotec B.V. & Co. KG, D
CD90	PE	Miltenyi Biotec B.V. & Co. KG, D
HLA-G	PE	Miltenyi Biotec B.V. & Co. KG, D

Table S5: antibodies for processed IF and IHC staining

Antigen	Company	Catalog number
Vimentin	Santa Cruz	SC-37317
CD31	Invitrogene	PA5-16301
CK18	ExBIO	11-107-C100
PD-L1	GeneTex	GTx57193
CD68	Dako	M0876
Calnexin	ThermoFischer	MA5-32332
CD44	ThermoFischer	PA5-114983
CK19	Avivasysbio	OABB00294/100ug
NTCP	Sigma	HPA042727
HNF4alpha	ThermoFischer	PA5-18363
Ki67	Origene	TA801156
Rb pAb Actin	Abcam	ab5694
EGFR	santa Cruz	sc-120
CK7	Dako Aglient	M7018
HBsAG	Dako Aglient	B0560
AFP	ThermoFischer	TA501788
Caspase3	Invitrogene	710431
AADAC	proteintech	26634-I-AP
MICA/B	eBioscience	5013153
HLA-G	ThermoFischer	PA5-98143

CD45-FITC	ThermoFischer	MHCD4520
HLA-ABC	Origene	SM1222LE

Table S6: PCR Primer pairs for cf-DNA measurement

Primer		Sequence (5'->3')
ALU_F_1	Forward primer	CCTGAGGTCAGGAGTTCGAG
ALU_R_1	Reverse primer	CCCGAGTAGCTGGGATTACA
ALU_F_2	Forward primer	GTGGCTCACGCCTGTAATC
ALU_R_2	Reverse primer	CAGGCTGGAGTGCAGTGG
MTCO_F_1	Forward primer	TAAACTTCAACCAACACCGT
MTCO_R_1	Reverse primer	TAGACTTCTGGGTGGCCAAAGA
MTCO_F_2	Forward primer	GACCTGATGCACTGAGGTTT
MTCO_R_2	Reverse primer	GTTTACGAGGCTTCTTCTG

Supplementary references

1. Kah J, Staffeldt L, Volz T, Schulze K, Heumann A, Rövenstrunk G, et al. Classification of the LC4 Primarily-like Cell Line—Recapitulating a CDK4 Overexpressing Immune Evasive HIV-HCV-Induced HCC. Viruses [Internet]. 2025; 17(5).
2. Staffeldt L, Mattert G, Riecken K, Rövenstrunk G, Volkmar A, Heumann A, et al. Generating Patient-Derived HCC Cell Lines Suitable for Predictive In Vitro and In Vivo Drug Screening by Orthotopic Transplantation. Cells. 2023;13(1).
3. Herzog N, Hansen M, Miethbauer S, Schmidtke KU, Anderer U, Lupp A, et al. Primary-like human hepatocytes genetically engineered to obtain proliferation competence display hepatic differentiation characteristics in monolayer and organotypical spheroid cultures. Cell Biol Int. 2016;40(3):341–53.
4. Burkard A, Dähn C, Heinz S, Zutavern A, Sonntag-Buck V, Maltman D, et al. Generation of proliferating human hepatocytes using Upcyte® technology: characterisation and applications in induction and cytotoxicity assays. Xenobiotica. 2012;42(10):939–56.
5. Wuestenberg A, Kah J, Singethan K, Sirma H, Keller AD, Rosal SR, et al. Matrix conditions and KLF2-dependent induction of heme oxygenase-1 modulate inhibition of HCV replication by fluvastatin. PLoS One. 2014;9(5):e96533.

6. Allweiss L, Volz T, Lutgehetmann M, Giersch K, Bornscheuer T, Lohse AW, et al. Immune cell responses are not required to induce substantial hepatitis B virus antigen decline during pegylated interferon-alpha administration. J Hepatol. 2014;60(3):500–7.
7. Linke C, von Hänisch T, Schröder J, Dammermann W, Deckert PM, Reinwald M, et al. Heterogeneous Formation of DNA Double-Strand Breaks and Cell-Free DNA in Leukemia T-Cell Line and Human Peripheral Blood Mononuclear Cells in Response to Topoisomerase II Inhibitors. Cancers (Basel). 2024;16(22).
